# Supplementary figures and images for: Decreased expression of Ly-1 antibody reactive clone (Lyar) triggers enhanced adipogenesis of bone marrow mesenchymal stromal cells in aged bone marrow
Source: PLoS One. 2026 May 27;21(5):e0349780. doi: 10.1371/journal.pone.0349780 (PMC13215539; doi:10.1371/journal.pone.0349780)

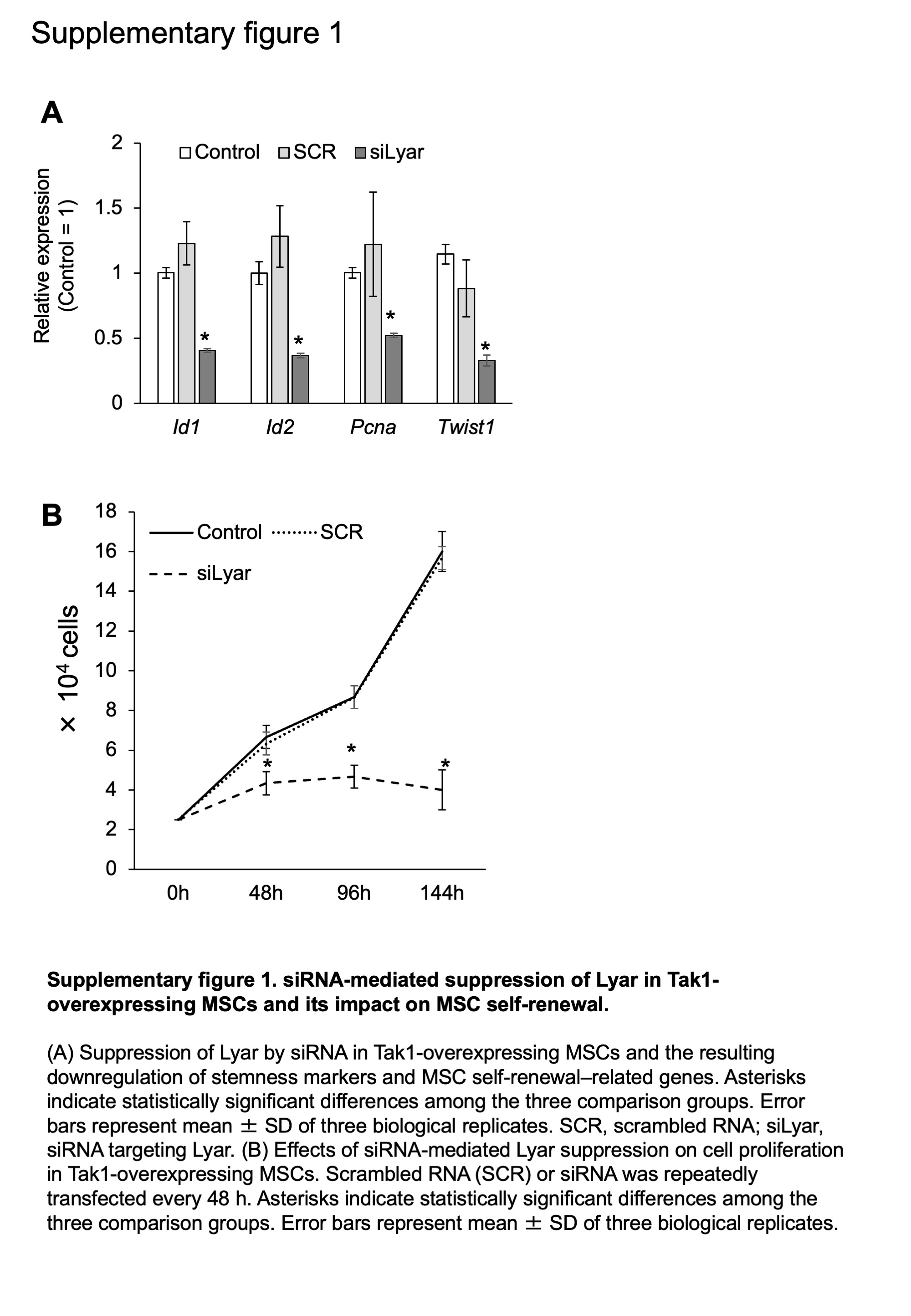

Supplement: S1 Fig — (JPEG) [file pone.0349780.s001.jpeg]

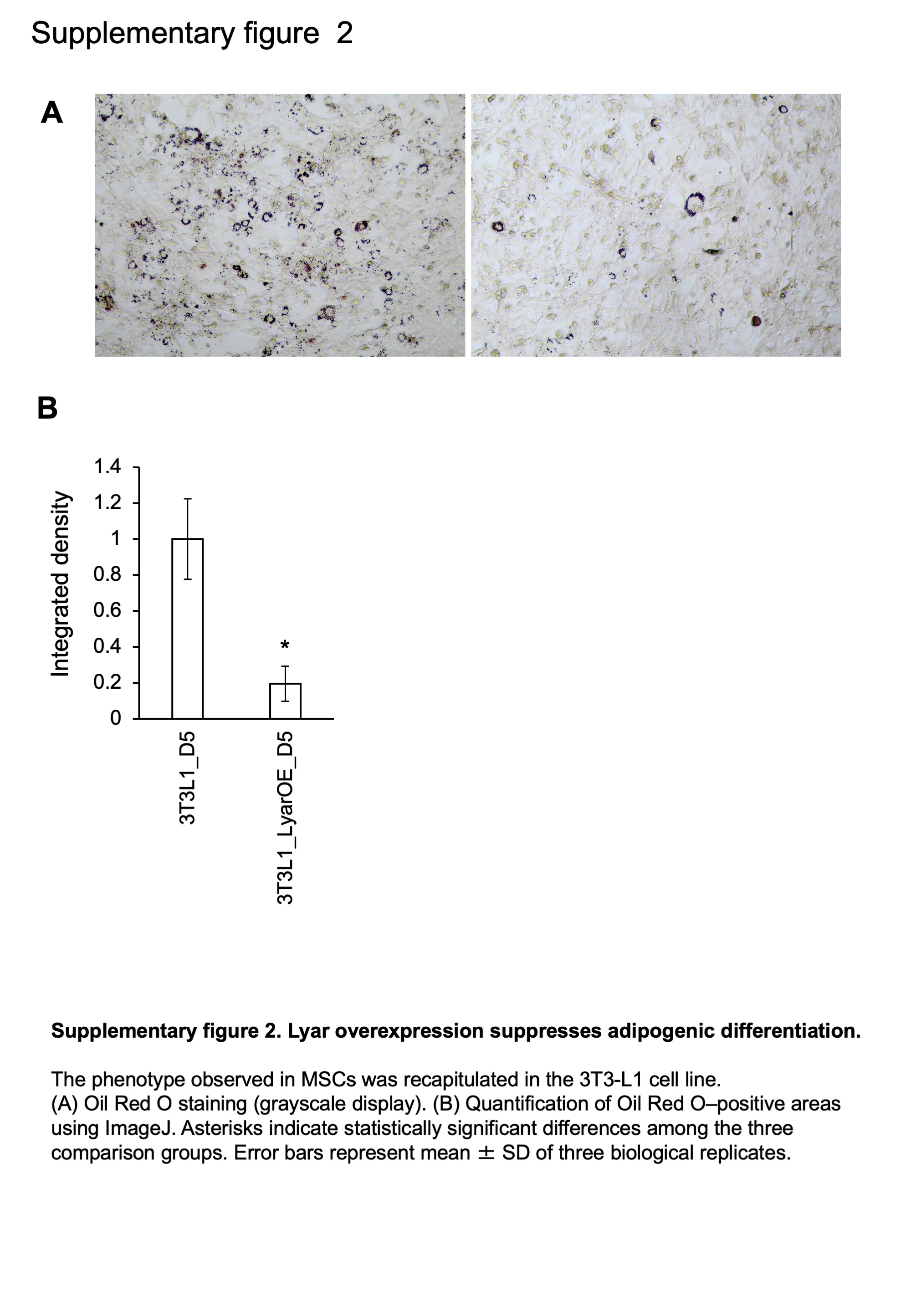

Supplement: S2 Fig — (JPEG) [file pone.0349780.s002.jpeg]
