# Supplementary material for: Decreased expression of Ly-1 antibody reactive clone (Lyar) triggers enhanced adipogenesis of bone marrow mesenchymal stromal cells in aged bone marrow
Source: PLoS One. 2026 May 27;21(5):e0349780. doi: 10.1371/journal.pone.0349780 (PMC13215539; doi:10.1371/journal.pone.0349780)

Supplementary information

Uncropped Western blot images corresponding to the main figures.

Figure1-B

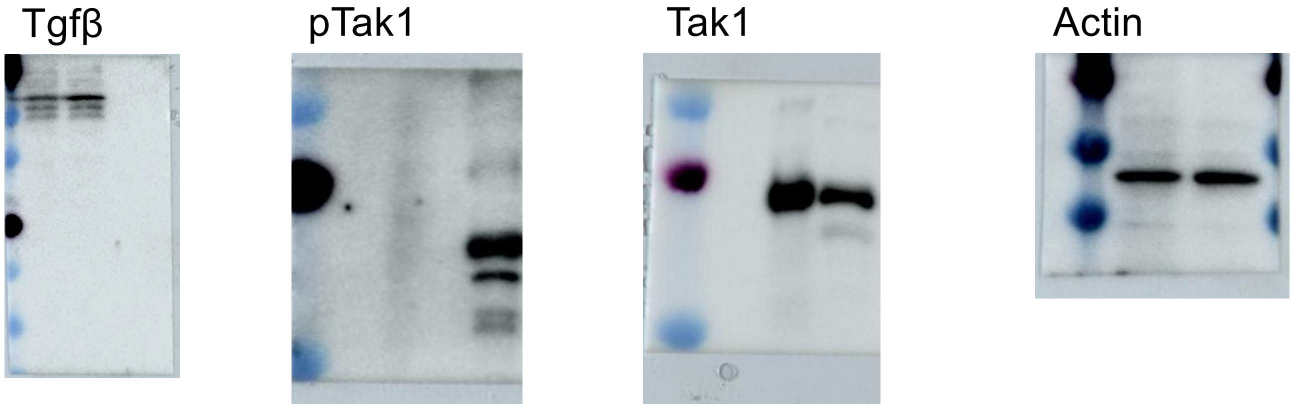

Figure1-D

Actin Pparγ Cebpα Fabp4

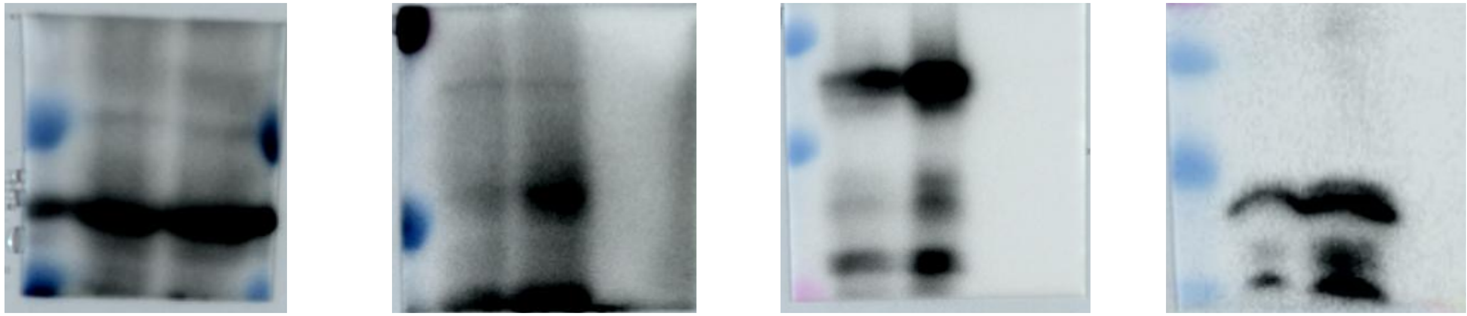

Figure2-A

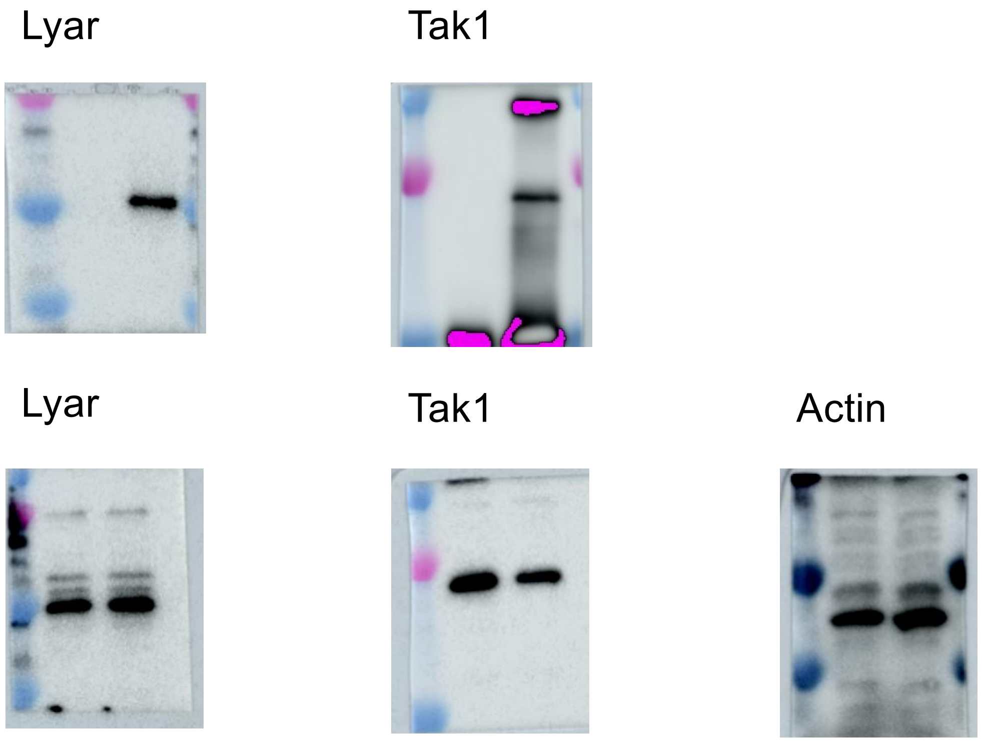

Figure 2-C

Actin

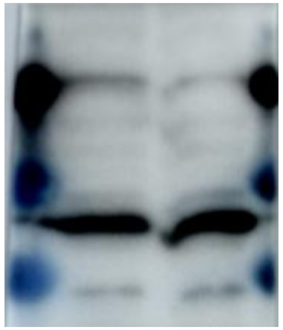

Lyar

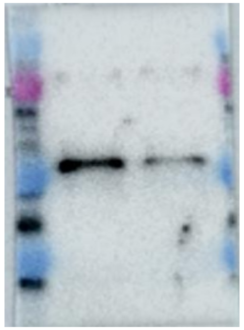

Figure3-C

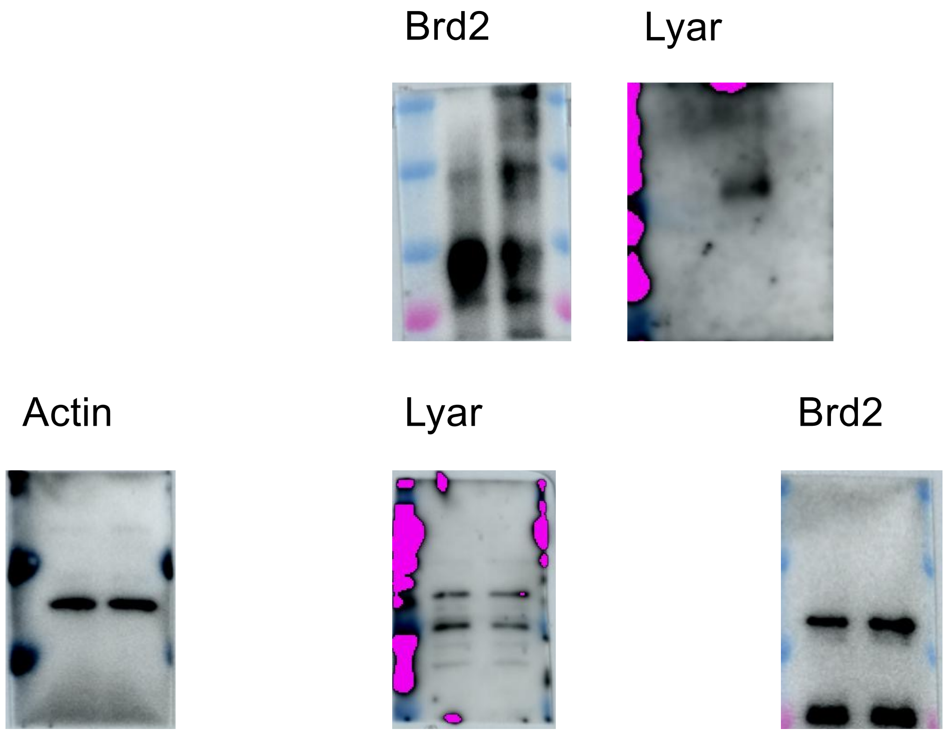

Figure4-B

Lyar

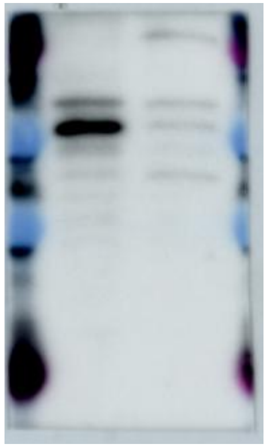

Actin

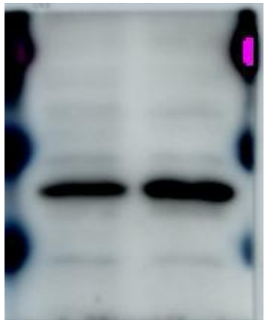

Figure4-F

Akt

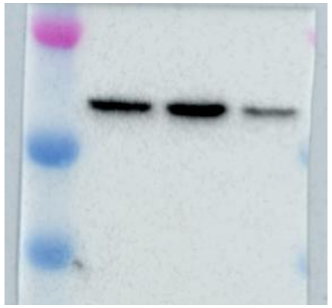

Lyar

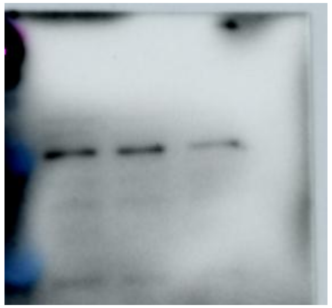

Actin

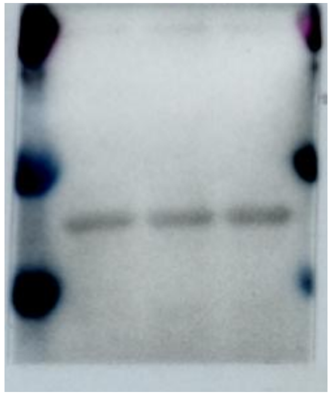

Supplement: S3 Fig — (PDF) [file pone.0349780.s003.pdf]
