# Supplementary material for: Decreased expression of Ly-1 antibody reactive clone (Lyar) triggers enhanced adipogenesis of bone marrow mesenchymal stromal cells in aged bone marrow
Source: PLoS One. 2026 May 27;21(5):e0349780. doi: 10.1371/journal.pone.0349780 (PMC13215539; doi:10.1371/journal.pone.0349780)
Supplement: S1 Table — (PDF) [file pone.0349780.s004.pdf]

Supplementary Table 1. Primer sequences used in qRT-PCR analysis.

Gene	Forward	Reverse
Lyar	ACAGCAGCAGGGAAACGGAAG	AGGCTGCTCTGGTAACTTCATC
Cebpα	AAGCCAAGAAGTCGGTGGAC	TCTGTTGCGTCTCCACGTTGC
Pparγ	AGCTGTCATTATTCTCAGTGG	TGTCTTGGATGTCCTCGATGG
Fabp4	ACCTGGAAGCTTGTCTCCAGTG	TTACGCTGATGATCATGTTGG
Runx2	GTTCAACGATCTGAGATTTGTG	GGATTTGTGAAGACTGTTATGG
Fabp5	TGATGGAAAGCCACGGCTTTG	TTCCTAAGAGCCAGTCCTACTC
Ucp2	TGTCAAACAGTTCTACACCAAG	ACCTTTACCACATCTGTAGGCTG
Gapdh	TCGTGGAGTCTACTGGTGTC	TCGTGGTTCACACCCATCAC
