# Supplementary material for: Decreased expression of Ly-1 antibody reactive clone (Lyar) triggers enhanced adipogenesis of bone marrow mesenchymal stromal cells in aged bone marrow
Source: PLoS One. 2026 May 27;21(5):e0349780. doi: 10.1371/journal.pone.0349780 (PMC13215539; doi:10.1371/journal.pone.0349780)
Supplement: S2 Table — (PDF) [file pone.0349780.s005.pdf]

Supplementary Table 2. Antibodies used in FACS and WB analyses in the present study.

Application	Antibody	Company	Cat No (Clone No)	Dilution	Condition
WB	Rabbit monoclonal anti-TAK1 antibody	CST	5206 (D94D7)	1 / 1,000 in Immuno-enhancer	4°C/Overnight
	Rabbit polyclonal anti-FABP4 antibody	CST	2120	1 / 1,000 in Immuno-enhancer	4°C/Overnight
	Rabbit monoclonal anti-C/EBPα antibody	CST	8178 (D56F10)	1 / 1,000 in Immuno-enhancer	4°C/Overnight
	Mouse monoclonal anti-PPARγ antibody	CST	95128 (D8I3Y)	1 / 1,000 in Immuno-enhancer	4°C/Overnight
	Rabbit polyclonal anti-TGF-β antibody	CST	3711	1 / 1,000 in Immuno-enhancer	4°C/Overnight
	Rabbit monoclonal anti-Brd2 antibody	CST	5848 (D89B4)	1 / 1,000 in Immuno-enhancer	4°C/Overnight
	Rabbit monoclonal anti-Phospho-TAK1(Thr184, Thr187) antibody	ThermoFisher Scientific	MA5-15073	1 / 1,000 in Immuno-enhancer	4°C/Overnight
	Goat polyclonal anti-Actin antibody	Santa Cruz Biotechnology	SC-1616 (I-19)	1 / 1,000 in Immuno-enhancer	4°C/Overnight
	Rabbit Polyclonal anti-Lyar antibody	ThermoFisher Scientific	PA5-44683	1 / 1,000 in Immuno-enhancer	4°C/Overnight
	Mouse monoclonal anti-Flag antibody	eBioscience	F3165	1 / 1,000 in Immuno-enhancer	4°C/Overnight
	Mouse monoclonal anti-HA antibody	Santa Cruz Biotechnology	SC-7392	1 / 1,000 in Immuno-enhancer	4°C/Overnight
	Rabbit monoclonal anti-TAK1 antibody	CST	5206 (D94D7)	1 / 1,000 in Immuno-enhancer	4°C/Overnight
	Rabbit polyclonal anti-FABP4 antibody	CST	2120	1 / 1,000 in Immuno-enhancer	4°C/Overnight
	Rabbit monoclonal anti-C/EBPα antibody	CST	8178 (D56F10)	1 / 1,000 in Immuno-enhancer	4°C/Overnight
	Mouse monoclonal anti-PPARγ antibody	CST	95128 (D8I3Y)	1 / 1,000 in Immuno-enhancer	4°C/Overnight
	Rabbit polyclonal anti-TGF-β antibody	CST	3711	1 / 1,000 in Immuno-enhancer	4°C/Overnight
FACS	CD45-FITC	Biolegend	103108(30-F11)	1 μg / 10^7 cells	RT / 30min
	TER119-FITC	Biolegend	116206	1 μg / 10^7 cells	RT / 30min
	PDGFRα-PE	BD Biosciences	562776(APA5)	1 μg / 10^7 cells	RT / 30min
	Ly-6A/E(Sca1)-APC	eBioscience	17-5981-83 (D7)	1 μg / 10^7 cells	RT / 30min
