# Supplementary material for: Decreased expression of Ly-1 antibody reactive clone (Lyar) triggers enhanced adipogenesis of bone marrow mesenchymal stromal cells in aged bone marrow
Source: PLoS One. 2026 May 27;21(5):e0349780. doi: 10.1371/journal.pone.0349780 (PMC13215539; doi:10.1371/journal.pone.0349780)
Supplement: S3 Table — (PDF) [file pone.0349780.s006.pdf]

Gene name	Identified Peptide Count	IgG	TAK1(aHA)	aHA/IgG
Spopl	5	1.70E+03	2.25E+06	1322.716158
Ccdc84	5	2.87E+04	9.13E+06	318.5601281
Fra10ac1	5	5.37E+03	1.39E+06	257.8692194
Phf11	11	4.01E+05	9.18E+07	228.6822503
Atp9b	5	9.44E+04	1.89E+07	199.7746309
Mta3	19	1.43E+06	1.30E+08	91.10390825
Fxr2	17	4.84E+06	3.49E+08	72.25669958
Magoh	8	5.38E+06	3.81E+08	70.93975467
Magohb	8	5.38E+06	3.81E+08	70.93975467
Map3k7(TAK1)	9	2.99E+05	1.60E+07	53.59215015
Atad5	5	2.88E+04	1.53E+06	52.9845115
Bclaf1	26	4.48E+06	2.17E+08	48.47855571
Ccdc9	12	1.43E+06	6.58E+07	46.01742689
Zc3h13	24	9.19E+05	4.04E+07	43.97271273
Gatad2b	14	1.07E+06	4.54E+07	42.53120158
Atxn2l	22	4.37E+06	1.83E+08	41.78894688
Phka2	7	1.03E+05	4.29E+06	41.64111756
Sema3c	20	8.75E+05	3.63E+07	41.45237003
Snrnp48	6	9.46E+04	3.89E+06	41.08408659
Mbd3	10	7.36E+05	2.81E+07	38.23636214
Smu1	15	3.76E+06	1.42E+08	37.64973678
Thoc3	6	4.96E+05	1.74E+07	35.04887153
Maff	5	5.19E+05	1.81E+07	34.81975334
Clk1	9	1.45E+05	5.01E+06	34.54904843
Rsrc1	5	8.45E+04	2.91E+06	34.46201375
Tra2b	8	6.64E+05	2.26E+07	33.98588612
Chd5	14	3.42E+04	1.14E+06	33.40690011
Grwd1	6	1.65E+05	5.39E+06	32.59107731
Fam120b	12	3.11E+05	9.96E+06	32.05523791
Nfil3	5	4.68E+04	1.44E+06	30.8188677
Rpp38	5	5.02E+05	1.55E+07	30.81163652
Cenpp	6	1.76E+05	5.34E+06	30.3739031
Nle1	10	7.12E+05	2.14E+07	30.1095339
Crnkl1	21	1.86E+06	5.26E+07	28.22903469
Thoc5	13	5.18E+05	1.44E+07	27.85479558

Ccdc130	6	2.35E+05	6.43E+06	27.36501522
Thoc1	14	9.96E+05	2.69E+07	26.96475934
Bud13	9	1.07E+06	2.89E+07	26.93928504
Celf1	5	9.52E+04	2.49E+06	26.13448527
Pabpn1	5	9.24E+05	2.39E+07	25.8341303
Nup160	19	3.04E+06	7.82E+07	25.74244571
Hdac1	14	3.64E+06	9.23E+07	25.34596101
Snrnp40	12	4.63E+06	1.17E+08	25.31888689
Rrp1	7	3.57E+05	9.01E+06	25.2759558
Thoc6	10	6.14E+05	1.54E+07	25.11156938
Sf3a2	6	1.33E+06	3.34E+07	25.0558693
Srpk1	16	1.61E+06	4.04E+07	25.04050321
Brpf1	8	1.61E+05	4.04E+06	25.03749816
Wdr74	12	6.81E+05	1.69E+07	24.79299866
Pdcd7	11	4.69E+05	1.15E+07	24.58629501
Nkrf	18	1.01E+06	2.42E+07	23.96984497
Hdac2	13	1.67E+06	3.83E+07	22.89523316
Krr1	9	1.97E+06	4.51E+07	22.88720825
Tra2a	6	1.90E+06	4.34E+07	22.88499936
Fxr1	25	9.84E+06	2.25E+08	22.85256568
Tarbp2	5	1.47E+05	3.34E+06	22.71876932
Eftud2	36	1.78E+07	4.02E+08	22.52617567
Hnrnpdl	6	7.84E+05	1.77E+07	22.52349754
Bcas2	10	1.34E+06	3.01E+07	22.39185576
Ppie	8	3.45E+05	7.73E+06	22.37801351
Ptcd3	5	8.27E+04	1.80E+06	21.74012617
Ccdc9b	13	8.12E+05	1.76E+07	21.65203718
Sarnp	6	9.44E+06	2.04E+08	21.56489409
Pak1ip1	7	3.23E+05	6.89E+06	21.37722171
Cdc40	17	2.95E+06	6.27E+07	21.23028258
Sf3a3	15	1.83E+06	3.84E+07	21.01555288
Pgam5	13	1.02E+07	2.12E+08	20.87723435
Tab2	12	4.57E+05	9.51E+06	20.81765664
Xab2	19	1.56E+06	3.23E+07	20.7864334
Gtf2h2	6	1.32E+05	2.71E+06	20.57695635
Toe1	14	1.22E+06	2.51E+07	20.56664243

Pwp1	11	2.40E+06	4.93E+07	20.51521444
Ik	10	1.72E+06	3.51E+07	20.47255927
Rpf1	8	4.91E+05	9.88E+06	20.1043217
Casc3	10	6.43E+05	1.29E+07	20.05091499
Pwp2	12	9.69E+05	1.94E+07	19.96992699
Nktr	10	3.17E+05	6.31E+06	19.90381644
Thoc2	23	1.93E+06	3.84E+07	19.87215356
Stau2	14	8.45E+05	1.66E+07	19.66780344
Phkb	10	5.80E+05	1.13E+07	19.55013075
Hnrnpc	8	4.43E+07	8.60E+08	19.42760256
Pnn	15	2.20E+06	4.27E+07	19.41587405
Acin1	13	1.55E+06	3.01E+07	19.35047474
Prpf8	73	2.80E+07	5.42E+08	19.33805445
Thoc7	7	3.12E+05	6.00E+06	19.23746067
Cenpo	6	4.24E+05	8.15E+06	19.20666768
Clk3	13	1.02E+06	1.95E+07	19.12655276
Hnrnpa2b1	19	2.46E+07	4.64E+08	18.81155081
Colec12	18	1.18E+06	2.21E+07	18.75405493
Mak16	6	1.48E+06	2.77E+07	18.73234014
Prpf19	10	9.41E+06	1.73E+08	18.41022043
Oasl2	13	1.93E+06	3.55E+07	18.36507873
Utp6	13	5.55E+05	1.02E+07	18.35735517
Trpv2	13	4.52E+06	8.24E+07	18.23481959
Sart1	20	1.51E+06	2.76E+07	18.21557654
Pop4	8	6.94E+05	1.26E+07	18.21135168
Mta2	16	3.44E+06	6.21E+07	18.03946892
Srsf6	8	2.30E+06	4.08E+07	17.7376131
Dgkz	9	7.24E+04	1.28E+06	17.68067777
Exosc2	6	3.88E+05	6.75E+06	17.41847838
Cenpu	6	3.36E+05	5.84E+06	17.39882626
Snu13	6	1.17E+07	2.04E+08	17.36792694
Zmat5	6	2.89E+05	5.02E+06	17.334342
Thrap3	24	1.77E+07	3.05E+08	17.297968
Brix1	10	6.09E+06	1.05E+08	17.23831076
Gmcl1	10	1.08E+06	1.82E+07	16.79436489
Snw1	15	3.46E+06	5.81E+07	16.78720897

Kat6b	10	1.61E+05	2.67E+06	16.59061084
Pdcd11	59	1.24E+07	2.04E+08	16.52963465
Bud31	8	2.97E+06	4.91E+07	16.50980507
Nop58	23	2.96E+07	4.85E+08	16.39412777
Nol11	10	7.95E+05	1.29E+07	16.17044671
Fam98a	10	1.80E+06	2.91E+07	16.13145592
Rsl1d1	11	9.00E+06	1.45E+08	16.07458069
Noc2l	11	2.55E+06	3.98E+07	15.64515052
Dnmt1	8	2.33E+06	3.61E+07	15.49883442
Dnajb6	5	1.52E+05	2.35E+06	15.45027742
Nsd1	10	2.30E+05	3.55E+06	15.41768909
Pum3	20	4.18E+06	6.36E+07	15.23034218
Ess2	8	1.94E+05	2.94E+06	15.15434932
Naa15	22	1.47E+06	2.22E+07	15.12299478
Eed	6	4.25E+05	6.40E+06	15.06439911
Arid1b	8	9.25E+04	1.39E+06	15.02910637
Utp15	9	6.73E+05	1.01E+07	14.9949767
Apobec3	14	1.16E+06	1.73E+07	14.9675591
Kat6a	15	6.74E+05	1.00E+07	14.86721046
Sf3b1	43	2.53E+07	3.76E+08	14.85537496
Sap18	7	7.61E+05	1.13E+07	14.83775387
Nr4a1	5	1.43E+05	2.12E+06	14.8183587
Ncbp1	16	1.87E+06	2.77E+07	14.77408184
Ptprs	5	2.68E+05	3.96E+06	14.74756327
Spats2l	10	2.54E+05	3.74E+06	14.68319527
Lyar	12	2.86E+06	4.19E+07	14.66123822
N4bp2l2	7	2.35E+05	3.43E+06	14.62265024
Cinp	5	1.99E+05	2.91E+06	14.61931455
Dkc1	21	7.06E+06	1.03E+08	14.58547256
Ebf3	9	1.12E+05	1.63E+06	14.54389888
Cdc5l	24	4.78E+06	6.96E+07	14.53934148
Ddx41	27	2.75E+06	4.00E+07	14.52317724
Pcbp2	9	1.55E+07	2.24E+08	14.47887116
Git1	8	3.84E+05	5.56E+06	14.47510487
Igf2bp1	9	1.28E+06	1.84E+07	14.32350018
Wdr3	25	2.08E+06	2.92E+07	14.01549387

Prkab2	5	9.67E+04	1.35E+06	13.96853123
Grn	6	2.68E+06	1.76E+06	0.65429028
