# Supplementary material for: Decreased expression of Ly-1 antibody reactive clone (Lyar) triggers enhanced adipogenesis of bone marrow mesenchymal stromal cells in aged bone marrow
Source: PLoS One. 2026 May 27;21(5):e0349780. doi: 10.1371/journal.pone.0349780 (PMC13215539; doi:10.1371/journal.pone.0349780)
Supplement: S4 Table — (PDF) [file pone.0349780.s007.pdf]

Yellow highlighting indicates the molecules of interest in this study

Red text indicates markers of aging and cellular senescence

Down regulated in Aged BMMSC			
Gene Symbol	Aged	Young	Young/Aged
Rcc2	0.01	50.27	5027
Eif2s1	0.06	20.88	348
Pnn	0.1	34.71	347.1
Nrp2	0.19	36	189.473684
Ipo5	0.08	14.26	178.25
Srp9	0.16	25.38	158.625
Igf2r	0.36	56.78	157.722222
Uso1	0.09	14.14	157.111111
Gsr	0.19	29.74	156.526316
Tln2	0.16	21.7	135.625
Nup98	0.24	28.87	120.291667
Sec61g	0.14	16.06	114.714286
Oga	0.94	104.56	111.234043
Eif3b	0.25	25.36	101.44
Scyl2	0.35	34.73	99.2285714
Ptpn6	0.16	12.18	76.125
Trpm4	0.63	47.13	74.8095238
Mak16	1.97	129.37	65.6700508
Tmem214	1.1	71.09	64.6272727
Adpgk	0.97	54.29	55.9690722
Wdr77	0.8	43.92	54.9
F8a1	1.44	75.23	52.2430556
Atp5pf	2.04	103.23	50.6029412
Bola2	1.79	89.31	49.8938547
Cdipt	0.54	22.85	42.3148148
Nup88	0.58	21.18	36.5172414
Brd4	0.95	34.55	36.3684211
Lsm12	2.07	75.19	36.3236715
Pfdn2	1.57	56.99	36.2993631
Gsn	0.82	27.94	34.0731707
Rpl22l1	0.28	9.29	33.1785714
Cdc42bpb	2.54	84.25	33.1692913
Rasa3	1.01	32.51	32.1881188
Setd3	2.12	66.86	31.5377358
Tpm3	0.8	23.9	29.875
Des	2.03	56.54	27.8522167
mKIAA1101	1.44	39.95	27.7430556
Gns	1.74	47.69	27.408046
Ralb	1.58	41.29	26.1329114
Actr2	0.57	14.69	25.7719298
Frmd8	0.29	7.42	25.5862069
Api5	1.46	35.46	24.2876712
Pitpnb	0.67	16.13	24.0746269
Snrpd3	4.78	111.6	23.3472803

Up regulated in Aged BMMSC			
Gene Symbol	Aged	Young	Young/Aged
Cat	48.82	24.4	0.49979517
Myh9	7.59	3.79	0.49934124
Adss1	36.75	18.35	0.49931973
Cisd2	39.98	19.96	0.49924962
Ppp2r1b	19.81	9.88	0.49873801
Ranbp9	11.94	5.95	0.49832496
Rps26	18.29	9.11	0.49808639
Itpr3	26.76	13.31	0.49738416
Bag2	16.71	8.31	0.497307
Tagln	39.76	19.75	0.49673038
Hmcn1	35.9	17.82	0.49637883
Dhx37	51	25.29	0.49588235
P3h1	42.83	21.23	0.4956806
Rbm15	18.53	9.18	0.49541284
Trio	17.08	8.45	0.49473068
Irgm1	32.35	15.98	0.49397218
Eif4g2	59.68	29.47	0.49380027
Eny2	134.27	66.26	0.49348328
Gars1	38	18.71	0.49236842
Fermt3	72.5	35.66	0.49186207
Capza2	32.95	16.2	0.49165402
Myl6	11.9	5.85	0.49159664
Rdh11	18.61	9.13	0.49059645
Dpep1	38.53	18.9	0.49052686
Rab8a	88.36	43.32	0.49026709
Palm	48.03	23.54	0.49011035
Acadm	29.31	14.36	0.48993518
Ap1b1	28.88	14.14	0.48961219
Tlr13	90.24	44.18	0.48958333
Sf1	79.45	38.84	0.48886092
Ptma	53.76	26.27	0.48865327
Srp54	23.37	11.41	0.48823278
Atp5f1c	36.17	17.65	0.48797346
Ywhaz	19.54	9.53	0.4877175
Sgsh	74.47	36.21	0.48623607
S100a11	39.98	19.4	0.48524262
Creg1	87.03	42.23	0.48523498
Hpf1	31.22	15.14	0.48494555
Arl8b	6.59	3.19	0.48406677
G3bp2	53.65	25.97	0.48406337
Macf1	61.41	29.72	0.48396027
Slc25a12	17.84	8.62	0.48318386
Atad1	104.96	50.49	0.4810404
Ctsa	28.98	13.89	0.47929607

Ufl1	0.47	10.83	23.0425532
Trip12	3.05	69.45	22.7704918
Epb41l3	0.97	21.99	22.6701031
Gfer	1.18	26.43	22.3983051
Mdp1	1.08	24.06	22.2777778
Hint1	1.36	29.61	21.7720588
Lsm7	2.5	54.08	21.632
Afg3l2	0.69	14.81	21.4637681
Capg	1.15	24.68	21.4608696
Nup153	3.13	60.8	19.4249201
Scfd1	1.2	22.8	19
Polr1c	3.92	73.14	18.6581633
Fkbp10	0.3	5.53	18.4333333
Slc25a5	1.48	27.05	18.277027
Pmm2	1.15	20.81	18.0956522
Rpl11	1.15	20.24	17.6
N6amt1	0.45	7.81	17.3555556
Clic4	1.87	31.93	17.0748663
Ndufs7	0.74	12.61	17.0405405
Fam98b	6.83	116.25	17.0204978
Ube2d2a	3.21	54.42	16.953271
Erap1	2.47	40.9	16.5587045
Tufm	1.53	25.22	16.4836601
Psma1	1.45	23.69	16.337931
Tln1	1.8	29.12	16.1777778
Actr1a	3.72	57.96	15.5806452
Atp6v1h	2.64	40.76	15.4393939
Arfgap2	0.89	13.51	15.1797753
Prkca	2.21	33.52	15.1674208
Aldh2	0.71	10.63	14.971831
Ubl7	0.67	9.78	14.5970149
Gcsh	1.5	21.86	14.5733333
Eif3g	0.6	8.69	14.4833333
Pgm3	7.92	113.28	14.3030303
Tnks1bp1	0.72	10.26	14.25
Hnrnpll	3.61	51.24	14.1939058
C1qbp	2.73	38.71	14.1794872
Dkc1	2.27	31.22	13.753304
Cct4	0.15	2.06	13.7333333
Atp5f1d	8.88	118.29	13.3209459
Atp5po	2.73	36.25	13.2783883
Eif2b5	4.34	57.61	13.2741935
Tax1bp3	0.89	11.7	13.1460674
Dhrs4	2.14	27.22	12.7196262
Emb	4.16	52.9	12.7163462
Bzw1	4.47	56.69	12.6823266
Aco2	2.32	27.66	11.9224138
Prkcsh	3.49	41.6	11.9197708
Serpinb6a	34.52	16.53	0.47885284
Ltbp2	30.1	14.4	0.47840532
Cd72	20.85	9.96	0.47769784
Cops3	93.95	44.84	0.47727515
Myl9	42	20.03	0.47690476
Rps23	47.12	22.46	0.47665535
Eps8l2	45.78	21.81	0.47640891
Ptx3	90	42.64	0.47377778
Rab34	54.67	25.88	0.47338577
Pum1	64.06	30.32	0.47330628
Stxbp3	30.95	14.64	0.473021
Rpl27a	6.41	3.03	0.47269891
H2ax	8.76	4.14	0.47260274
Iqgap1	9.27	4.38	0.47249191
Clybl	40.63	19.17	0.47181885
Rap2c	22	10.38	0.47181818
Eno1	14.54	6.86	0.47180193
Itgb1	24.62	11.61	0.47156783
Cops4	33.25	15.66	0.47097744
Sec23b	63.64	29.92	0.47014456
Ido1	104.79	49.23	0.46979674
Ehd2	30.32	14.24	0.46965699
Plcg1	46.1	21.58	0.4681128
Commd3	10.15	4.75	0.4679803
Atp6v1d	22.86	10.69	0.46762905
Tmx2	28.63	13.38	0.46734195
Ttc39b	53.73	25.06	0.4664061
Fmnl1	76.6	35.67	0.4656658
Pitrm1	32.03	14.89	0.46487668
Tmem43	51.74	24.04	0.46463085
Actb	18.04	8.38	0.46452328
Prkcb	49.33	22.9	0.46422056
Usp14	16.31	7.57	0.46413243
Vcl	16.29	7.55	0.46347452
App	44.02	20.38	0.46297138
Apoo	6.42	2.96	0.46105919
Col2a1	50.97	23.5	0.46105552
Samhd1	38.59	17.79	0.46100026
Map1a	62.87	28.9	0.4596787
Glb1	29.03	13.3	0.45814674
Nop58	52.2	23.86	0.45708812
Mtr	94.93	43.36	0.45675761
Srsf5	59.52	27.18	0.45665323
Itga6	120.98	55.13	0.45569516
Ctsh	93.86	42.67	0.45461325
Tpm4	17.57	7.97	0.45361411
Srsf7	50.36	22.81	0.45293884
Smpdl3a	87.66	39.68	0.452658

Galnt2	2.8	33.18	11.85
Yipf3	3.96	46.73	11.8005051
Washc5	1.4	16.37	11.6928571
Kbtbd3	2.09	23.27	11.1339713
Card19	1.81	19.95	11.0220994
Clip1	4.97	54.32	10.9295775
Hmgn2	12.3	132.79	10.795935
Sacm1l	5.38	57.27	10.6449814
Ythdc1	7.7	81.79	10.6220779
Gosr1	1.93	20.49	10.6165803
Plbd2	1.16	12.29	10.5948276
Mrpl41	9.29	97.77	10.5242196
Smarca5	4.7	49.39	10.5085106
Msto1	2.79	29.06	10.4157706
Sparc	0.69	6.99	10.1304348
Prpf4	6.81	68.87	10.113069
Hirip3	6.31	63.75	10.1030111
Arhgef2	6.22	62.64	10.0707395
Srsf10	3.35	33.73	10.0686567
Uqcrq	2.2	22.01	10.0045455
Ddi2	4.25	42.42	9.98117647
Rpl29	1.57	15.63	9.95541401
Cand1	0.36	3.57	9.91666667
Cybb	3.89	38.48	9.89203085
Txndc5	2.76	27.14	9.83333333
Ccn1	2.65	25.92	9.78113208
Nelfa	9.81	94.43	9.62589195
Tecr	3.41	32.8	9.61876833
Vps53	6.28	59.86	9.53184713
Snrpa	0.97	9.15	9.43298969
Tmed5	11.1	102.22	9.20900901
Nup205	2.88	26.49	9.19791667
Lgals3bp	6.56	60.27	9.1875
Vasn	2.57	23.56	9.16731518
Smad2	0.27	2.47	9.14814815
Ywhab	2.41	21.66	8.98755187
Magt1	3.39	29.94	8.83185841
Tbl2	9.87	87.07	8.82168186
Kras	5.83	51.11	8.76672384
Lrpap1	4.19	36.56	8.72553699
Hcfc1	9.07	79.06	8.71664829
Aldh1l2	2.86	24.76	8.65734266
Rad21	3.59	30.84	8.59052925
Tial1	5.18	44.29	8.55019305
Rbms1	9.14	77.67	8.49781182
Ggcx	3.53	29.59	8.38243626
Ftl1	7.75	64.55	8.32903226
Noc4l	6.24	51.69	8.28365385

Themis2	100.62	45.51	0.45229577
Csrp2	36.65	16.51	0.45047749
Acin1	34.14	15.36	0.44991213
Rnaseh2a	7.07	3.18	0.44978784
Mthfd1l	24.64	11.08	0.44967532
Ccdc43	25.74	11.55	0.44871795
aco1	46.78	20.98	0.44848226
Steap2	51.21	22.92	0.44756883
Uqcrc2	60.61	27.12	0.44745092
Npepps	43.74	19.56	0.44718793
Hexb	75.21	33.59	0.44661614
Gnai2	32.35	14.44	0.44636785
Tbxas1	47.35	21.12	0.44604013
Ap3b1	44.2	19.65	0.44457014
H4c1	20.07	8.91	0.44394619
Tmod3	31.71	14.07	0.44370861
Gemin5	69.1	30.64	0.44341534
Tha1	69.25	30.65	0.44259928
Blvrb	36.24	16.03	0.44232892
Hmgcl	67.61	29.87	0.44179855
Bcas2	40.28	17.73	0.44016882
Prkar2a	5.43	2.39	0.44014733
H1-4	69.84	30.72	0.43986254
Atp6v1b2	26.19	11.52	0.43986254
Mroh6	71.73	31.54	0.43970445
Ivd	35.32	15.49	0.43856172
Bin1	119.81	52.37	0.43710876
Numa1	17.67	7.72	0.4368987
Abi1	46.69	20.37	0.43628186
F13a1	65.41	28.5	0.43571319
Rpl30	52.31	22.79	0.43567196
Copb1	19.61	8.53	0.43498215
H2bw2	102.29	44.49	0.43493988
Afap1	19.13	8.27	0.43230528
Pon3	31.65	13.66	0.43159558
Kidins220	47.76	20.56	0.43048576
Entpd1	71.51	30.78	0.43042931
Pds5a	42.77	18.4	0.43020809
Acads	16.38	7.04	0.42979243
Pgrmc1	22.69	9.74	0.42926399
Srprb	44.2	18.97	0.42918552
Eml4	23	9.87	0.42913043
Itgav	30.26	12.96	0.42828817
Rpl13	38.95	16.66	0.42772786
Tomm6	61.37	26.15	0.42610396
Fkbp9	16.08	6.85	0.42599502
Isoc2a	68.78	29.28	0.42570515
Vdac1	56.35	23.97	0.42537711

Vcp	1.22	10.08	8.26229508
Tbc1d5	4.63	38.22	8.25485961
Ap1s1	1.65	13.59	8.23636364
Riox1	3.38	27.64	8.17751479
Myo1d	1.73	14.14	8.1734104
Cyc1	6.12	48.99	8.00490196
Prrc2c	7.55	59.29	7.85298013
Acat1	6.32	49.52	7.83544304
Fth1	5.8	45.3	7.81034483
Fscn1	7.33	56.42	7.69713506
Cmas	2.48	18.97	7.64919355
Map1s	2.44	18.51	7.58606557
Lrpprc	4.9	37.17	7.58571429
Cyp20a1	5.15	39	7.57281553
Atp5pd	2.93	22.15	7.55972696
Fkbp8	2.21	16.63	7.52488688
Hmgb2	1.74	12.97	7.454022989
Col5a2	4.11	30.55	7.43309002
Sucla2	6.63	48.84	7.36651584
Rnf213	4.77	34.43	7.21802935
Cad	3.93	28.32	7.20610687
Cstf2	0.84	6.05	7.20238095
Rpa3	9.84	70.39	7.15345528
Vim	2.55	18.15	7.11764706
Thbs2	11.44	81.1	7.08916084
Gclc	10.51	73.49	6.9923882
Abcf3	2.56	17.85	6.97265625
Zc2hc1a	3.19	22.19	6.95611285
Minpp1	14.97	103.35	6.90380762
Pkm	2.77	18.8	6.78700361
Ampd2	4.7	31.44	6.6893617
Hnrnpf	3.28	21.89	6.67378049
Larp4b	6.53	43.23	6.6202144
Rbmxl1	8.03	52.93	6.59153176
Bcap31	4.77	31.14	6.52830189
Nup188	1.45	9.43	6.50344828
Prkra	9	58.29	6.47666667
Eftud2	3.59	23.05	6.42061281
Mbnl1	12.86	82.43	6.40979782
Slc25a22	5.95	37.34	6.27563025
Mrfap1	5.75	36.03	6.26608696
Cox5b	2.95	18.24	6.18305085
Znf516	7.87	48.64	6.18043202
Dnajb6	4.14	25.49	6.15700483
Mindy1	2.06	12.53	6.08252427
Pdia3	1.74	10.49	6.02873563
Slc7a8	8.92	53.69	6.0190583
Reep5	9.1	54.67	6.00769231

Hba-a1	21.79	9.26	0.42496558
Rhog	82.77	35.12	0.42430832
Ak2	31.89	13.51	0.42364378
Picalm	15.77	6.67	0.42295498
Phax	33.11	14	0.42283298
Ran	18.08	7.63	0.42201327
Kars1	54.14	22.84	0.42186923
Plcd1	109.35	46.12	0.42176497
Rbsn	34.77	14.57	0.4190394
Dera	123.28	51.63	0.41880273
gag	66.1	27.68	0.41875946
Atp1b3	86.74	36.31	0.41860733
Cavin2	35.33	14.78	0.41834135
Pcif1	51.57	21.57	0.41826643
Ipo7	14.04	5.87	0.41809117
Msr1	43	17.95	0.41744186
0610010K14Rik	31.69	13.22	0.4171663
Pfdn5	71.57	29.78	0.41609613
Lgals9	24.58	10.22	0.41578519
Nup62	124.06	51.57	0.41568596
Pdcd6ip	20.15	8.36	0.41488834
Rgs10	130.59	54.01	0.4135845
Ifi30	67.13	27.76	0.41352599
Umps	44.66	18.43	0.41267353
Cdkn2aip	18.6	7.66	0.41182796
Ndufb7	25.79	10.62	0.41178751
Strn	48.35	19.9	0.41158221
Snrpb	51.87	21.32	0.41102757
Dpm1	40.66	16.71	0.41096901
Tnpo1	19.61	8.05	0.41050484
Fasn	13.72	5.63	0.41034985
Tyms	96.16	39.44	0.41014975
Rbm3	54.55	22.2	0.40696609
Srp14	45.15	18.35	0.40642303
Tubb6	8.49	3.44	0.40518257
Rbbp9	38.7	15.68	0.40516796
Cavin3	15.88	6.43	0.40491184
Rnmt	13.36	5.4	0.40419162
Mtch2	11.62	4.69	0.40361446
Cops5	93.51	37.72	0.40337932
Dclk1	28.96	11.67	0.40296961
Snrpa1	54.13	21.8	0.40273416
Aebp1	60.5	24.3	0.40165289
Hadha	18.99	7.59	0.39968404
Coro1c	28.1	11.23	0.39964413
Ykt6	71.69	28.64	0.39949784
Msrb3	98.2	39.18	0.39898167
Nae1	45.42	18.09	0.39828269

Klc1	5.74	34.27	5.97038328
Cfap210	14.71	87.64	5.9578518
Eps8	2.98	17.74	5.95302013
Sh3bgrl3	17.95	106.61	5.93927577
Gspt1	12.13	71.66	5.90766694
Eif2a	5.61	32.88	5.86096257
H6pd	10.36	60.65	5.8542471
Trappc1	1.07	6.25	5.8411215
Metap2	1.59	9.28	5.83647799
P4hb	1.59	9.27	5.83018868
Snrnp200	7.57	44.11	5.82694848
Acp5	17.53	101.8	5.80718768
Naa38	3.71	21.46	5.78436658
Slc25a3	5.07	29.22	5.76331361
Arpc5	15.24	87.75	5.75787402
Ddost	2.23	12.8	5.73991031
Clec12a	12.09	68.49	5.66501241
Arf5	9.98	56.21	5.63226453
Cpd	10.01	56.23	5.61738262
Rcn2	3.39	18.97	5.59587021
Shmt2	4.51	25.07	5.55875831
Ddx17	1.85	10.22	5.52432432
Psmb4	9.85	54.31	5.51370558
Cyb5b	3.61	19.69	5.45429363
Ilf2	6.07	33.02	5.4398682
Nup133	5.96	32.38	5.43288591
Rfc5	5.86	31.77	5.42150171
Rpl5	2.77	14.89	5.37545126
Ap2a1	4.49	23.96	5.3363029
Prdm1	11.03	58.85	5.33544878
Tacc3	7.08	37.55	5.30367232
Tmx1	8.4	44.37	5.28214286
Ddx47	10.58	55.63	5.25803403
Rpl27	3.88	20.25	5.21907216
Cog2	9.22	48.02	5.20824295
Gla	5.64	28.98	5.13829787
Atp5mg	2.73	13.88	5.08424908
Yipf5	3.85	19.51	5.06753247
Nptn	8.62	43.37	5.03132251
Anxa4	12.8	64.09	5.00703125
Cyth3	16.5	82.53	5.00181818
Tap2	21.47	107.29	4.9972054
Rtn3	26.29	131.37	4.99695702
Mtpn	6.67	33.32	4.99550225
Akt1	9.12	45.42	4.980263158
Anp32b	4.43	22.03	4.97291196
Hnrnpu	6.42	31.91	4.97040498
Pcca	0.5	2.48	4.96

Psmd4	2.31	0.92	0.3982684
Ppp2r5e	1.51	0.6	0.39735099
Gmppb	18.6	7.38	0.39677419
Pcbd2	45.23	17.92	0.39619721
Isoc1	106.47	41.9	0.39353809
Myh11	61.98	24.39	0.39351404
Thoc2	16.78	6.6	0.39332539
Commd9	76.59	30.08	0.39274057
Fcho2	63.68	24.95	0.39180276
Lars1	4.78	1.87	0.39121339
Anpep	33.64	13.16	0.39120095
Hspbp1	26.87	10.5	0.39077038
Cd2ap	43.37	16.94	0.39059258
Apeh	43.37	16.94	0.39059258
Cse1l	42.43	16.56	0.39028989
Emc1	48.41	18.89	0.39020863
Actn1	24.97	9.72	0.38926712
Taf15	27.27	10.59	0.38833883
Brk1	37.9	14.67	0.38707124
Emc4	27.3	10.56	0.38681319
Mcm6	69.27	26.75	0.38617006
Trim32	29.29	11.3	0.3857972
Mrps25	58.57	22.55	0.38500939
Sorbs3	38.03	14.62	0.38443334
Rnf17	38.49	14.79	0.38425565
Banf1	127.48	48.88	0.3834327
Gnas	4.88	1.87	0.38319672
Birc6	77.89	29.83	0.38297599
Sfpq	35.96	13.71	0.38125695
Rab5c	27.2	10.31	0.37904412
Myo1b	45.17	17.11	0.37879123
Ddx46	67.84	25.67	0.37839033
Tsen34	42.52	16.06	0.37770461
Rpl39	39.84	15.04	0.37751004
Dnm1	55.06	20.69	0.37577189
Lamp2	58.87	22.12	0.37574316
Ipo13	23.16	8.7	0.37564767
Tma7	42.24	15.85	0.37523674
Abhd12	37.07	13.91	0.37523604
Cttn	21.42	8.02	0.37441643
Plaa	68.32	25.54	0.37382904
Abcg2	24.03	8.98	0.37369954
Eef1g	40.71	15.21	0.37361828
Lemd3	115.24	42.96	0.37278723
Hnrnpa3	56.3	20.96	0.3722913
Ddx54	122.09	45.45	0.37226636
Farsa	23.21	8.64	0.37225334
Bag6	52.42	19.51	0.37218619

Arrb2	14.4	71.4	4.95833333	Arpin	82.26	30.53	0.37114029
Ntmt1	4.98	24.61	4.94176707	Ethe1	31.91	11.83	0.37073018
Uggt1	6.16	30	4.87012987	Ak3	66.59	24.56	0.36882415
Gbf1	3.5	17.03	4.86571429	Lmf2	18.34	6.74	0.36750273
Mtap	14.72	70.49	4.78872283	Nmral1	24.27	8.9	0.36670787
Qki	6.57	31.41	4.78082192	Creb1	41.37	15.16	0.36644912
Rbm39	4.23	20.18	4.77068558	Dnpep	10.9	3.97	0.36422018
Supt6h	10.59	50.36	4.75542965	Ppp3ca	39.77	14.46	0.36359065
Usp9x	2.91	13.7	4.70790378	Ppp1r9b	61.15	22.2	0.3630417
Fundc2	4.55	21.4	4.7032967	Eif4e2	24.6	8.91	0.36219512
ATP8	3.9	18.23	4.67435897	Ctnna1	39.6	14.29	0.36085859
Cd248	5.15	24.07	4.67378641	Cnot7	84.34	30.42	0.36068295
Nmt1	25.18	117.61	4.67077045	Rabep2	36.49	13.15	0.3603727
Atg3	9.32	43.5	4.66738197	Magohb	23.42	8.42	0.35952178
Letm1	6.12	28.24	4.61437908	Prmt5	44.07	15.84	0.35942818
Cep170	5.75	26.52	4.61217391	Dctn4	10.81	3.87	0.35800185
Srp19	7.52	34.66	4.60904255	Ctnnbl1	35.03	12.53	0.35769341
H1-2	2.18	10.03	4.60091743	Rab5b	56.15	19.97	0.3556545
Sh3pxd2b	14.9	68.51	4.59798658	Crip2	62.62	22.26	0.35547748
Sdf4	27.54	126.37	4.5885984	Pelp1	57.72	20.49	0.3549896
Ncam1	6.57	30.12	4.58447489	Sec24c	13.24	4.7	0.35498489
G6pdx	5.69	26.02	4.57293497	Hbbt1	13.54	4.8	0.35450517
Skic2	29.09	132.53	4.55586112	Ipo9	33.48	11.86	0.35424134
Lman2	6.52	29.29	4.49233129	Trim47	87.8	31.05	0.35364465
Aida	18.67	83.52	4.47348688	Nhp2	72.38	25.59	0.3535507
Fhl3	11.29	50.44	4.4676705	Flii	39.83	14.05	0.35274918
Dcaf8	0.66	2.93	4.43939394	Palld	71.42	25.18	0.35256231
Exosc6	6.88	30.35	4.41133721	Scamp2	117.12	41.2	0.35177596
Stk4	16.54	72.76	4.39903265	Blmh	26.33	9.26	0.35169009
Zfpl1	29.05	127.51	4.38932874	Loxl3	85.87	30.14	0.35099569
Ifi209	1.86	8.14	4.37634409	Gnb2	37.49	13.15	0.3507602
Psmd5	1.44	6.3	4.375	Adprh	62.9	22.02	0.35007949
Hook3	7.41	32.24	4.35087719	Pdap1	89.96	31.49	0.35004446
Rps2	6.15	26.66	4.33495935	Ube4b	18.52	6.47	0.34935205
Elp2	16.57	71.59	4.32045866	Akr1b10	83.54	29.15	0.34893464
Aprt	5.92	25.51	4.30912162	Ppp1r7	59.27	20.67	0.34874304
Drg1	3.81	16.33	4.28608924	Stag1	4.6	1.6	0.34782609
Hectd1	21.26	91.06	4.28316087	Rpl13a	27.62	9.6	0.34757422
Ganab	3.09	13.22	4.27831715	H2bc3	141	48.89	0.34673759
Tprkb	10.33	44.14	4.27299129	Pdk1	127.05	44.03	0.346556474
Use1	18.27	77.78	4.25725233	H2bc12	32.89	11.37	0.34569778
Higd1a	2.15	9.12	4.24186047	Xdh	49.29	17.01	0.34510043
Ndufa2	10.43	44.24	4.24161074	Csnk2b-Ly6g5t	21.22	7.32	0.34495759
Lrrc47	1.09	4.59	4.21100917	Tuba4a	34.2	11.79	0.34473684
Gnpnat1	14.8	62.21	4.20337838	Naxd	15.94	5.47	0.34316186
Nup35	5.88	24.46	4.15986395	Pnkp	55.66	19.1	0.34315487
Fbl	8.8	36.6	4.15909091	Rwdd4	22.45	7.7	0.34298441
Wdr26	2.87	11.87	4.1358885	Gpnmb	74.34	25.49	0.34288405

Psmb10	4.86	20	4.11522634	Aldh7a1	50.38	17.2	0.34140532
M6pr	3.95	16.18	4.09620253	Adnp	71.09	24.19	0.34027289
Alg5	5.83	23.81	4.08404803	Srp72	33.98	11.55	0.33990583
Nap1l1	5.01	20.4	4.07185629	Cr1l	37.25	12.66	0.33986577
Lsm14a	5.25	21.2	4.03809524	Snapin	42.41	14.41	0.33977835
Ubr7	11.58	46.72	4.03454231	Lrrc59	17.19	5.83	0.33915067
Immt	8.78	35.42	4.03416856	Hist1h3i	54.24	18.36	0.33849558
Hnrnpa3	5.02	20.22	4.02788845	Psmd3	21.78	7.34	0.33700643
Fh	12.76	51.02	3.9984326	Srrm1	18.25	6.14	0.33643836
Got2	1.87	7.47	3.99465241	Alb	21.38	7.17	0.33536015
Cops7a	1.52	6.07	3.99342105	Mtfr1l	61.16	20.5	0.3351864
H1-1	3.6	14.34	3.98333333	Selenoi	48.94	16.4	0.33510421
Zzef1	2.65	10.55	3.98113208	Tmem50a	36.24	12.13	0.33471302
Farp1	7.02	27.83	3.96438746	Ehhadh	72.2	24.06	0.333241
Dctn2	5.58	21.97	3.93727599	Ly75	30.15	10.04	0.33300166
Grhpr	11.89	46.6	3.91925988	Snx6	77.66	25.82	0.33247489
Mix23	26.97	105.43	3.90915832	Copb2	34.04	11.31	0.33225617
Cert1	4.35	17	3.90804598	Degs1	102.73	34.11	0.33203543
Hmox2	0.8	3.12	3.9	Rala	22.91	7.56	0.32998691
Abracl	5.95	23	3.86554622	Pfdn6	15.98	5.27	0.32978723
Tbcd	27.24	105.2	3.86196769	Cope	26.08	8.58	0.32898773
Inppl1	5.51	21.27	3.86025408	Vrk2	40.61	13.35	0.32873676
Slk	3.71	14.3	3.85444744	Pafah1b3	65.72	21.56	0.32805843
Me2	20.21	77.85	3.85205344	Tfg	16.95	5.55	0.32743363
Dtymk	4.56	17.49	3.83552632	Psmg1	17.26	5.65	0.32734647
Sgta	9.59	36.76	3.83315954	Immt	98.94	32.26	0.3260562
Chpf	13.79	52.73	3.82378535	Gsto1	28.83	9.39	0.32570239
Wars1	6.99	26.72	3.82260372	Stim1	57.05	18.58	0.32567923
Slc38a7	11.66	44.56	3.82161235	Ppic	38.1	12.37	0.32467192
Psmb7	1.84	6.98	3.79347826	Tmsb10	9.92	3.21	0.32358871
Gyg1	29.09	110.11	3.78514954	Otub1	17.42	5.62	0.32261768
Gba1	9.04	34.21	3.78429204	Pdia5	22.91	7.38	0.32213007
Folr2	8.79	33.21	3.778157	Psma7	61.02	19.65	0.32202557
Rbfox2	15.52	58.62	3.77706186	Vps26c	91.38	29.29	0.32052966
NG1	4.39	16.55	3.76993166	Stxbp1	35.35	11.27	0.31881188
Rps27l	12.09	45.49	3.76261373	Eif4b	23.2	7.38	0.31810345
Arcn1	1.59	5.98	3.76100629	Sgpp1	10.73	3.41	0.31780056
Atp5me	6.18	23.24	3.7605178	Diaph2	95.7	30.23	0.31588297
Pus7	6.05	22.72	3.7553719	Pgm1	36.53	11.53	0.31563099
Plec	6.77	25.42	3.75480059	Vps51	18.16	5.72	0.31497797
Tor3a	4.75	17.76	3.73894737	Pak1	54.66	17.2	0.31467252
Rpl23	4.52	16.86	3.7300885	Sod1	15.84	4.98	0.31439394
Nup93	16	59.67	3.729375	Psmg2	12.85	4.03	0.31361868
Yme1l1	7.22	26.66	3.69252078	Zdhhc20	17.92	5.62	0.31361607
Vamp8	12.71	46.63	3.66876475	Dazap1	55.61	17.42	0.31325301
Ppp1ca	6.03	22.09	3.66334992	Spr	59.03	18.45	0.31255294
Lpp	4.74	17.35	3.66033755	Supt4a	19.2	6	0.3125
Ndufa9	34.5	125.85	3.64782609	Lclat1	47.74	14.86	0.31126938

Cst3	4.89	17.83	3.64621677	Rpl37	28.24	8.76	0.3101983
Oas1a	18.93	68.99	3.64447966	Chil3	64.81	20.1	0.31013732
Dnmt1	18.52	67.34	3.63606911	Kif2a	43.41	13.42	0.30914536
Sar1a	6.91	25.08	3.62952243	Xrn2	57.11	17.59	0.3080021
Inf2	23.22	83.53	3.59732989	Grb10	40.17	12.26	0.30520289
Lgals3	4.72	16.92	3.58474576	Gcdh	29.1	8.87	0.304811
Ccn2	17.97	63.97	3.55982193	Bzw2	12.87	3.92	0.3045843
Rprd1b	1.95	6.91	3.54358974	Nenf	20.93	6.35	0.30339226
Ilf3	8.67	30.64	3.53402537	Nsfl1c	33.46	10.13	0.30274955
Phldb2	6.23	21.86	3.50882825	Eif4g1	20.84	6.3	0.30230326
Kiaa2013	31.81	111.53	3.50613015	Wars2	13.72	4.14	0.30174927
Rps19	9.59	33.55	3.49843587	Timm44	31.74	9.57	0.30151229
Kgd4	13.71	47.94	3.49671772	Txn	42.88	12.9	0.30083955
Smc3	22.7	79.26	3.49162996	Cct5	18.87	5.66	0.29994701
Snrpf	6.35	22.12	3.48346457	Jpt1	94.99	28.45	0.29950521
Fabp5	28.33	98.32	3.47052594	P4ha1	37.14	11.12	0.29940765
Ogfod3	5.23	18.15	3.47036329	Prdx6	39.21	11.66	0.29737312
mg684	8.89	30.85	3.47019123	Rbm14	20.55	6.1	0.29683698
Sec24b	13.3	46.01	3.4593985	Matr3	37.9	11.25	0.29683377
Anks1	18.17	62.54	3.44193726	Pgam2	18.78	5.57	0.29659212
Piezo1	9.41	32.28	3.4303932	Prdx2	46.63	13.82	0.29637572
Sptan1	2.34	7.97	3.40598291	Clip2	53.54	15.85	0.29604034
Mrpl11	28.43	96.49	3.39395005	Eef1a1	39.44	11.66	0.29563895
Calr	11.29	38.07	3.37201063	Rap2b	38.42	11.33	0.29489849
Gm9392	6.75	22.7	3.36296296	Tbck	78.89	23.22	0.29433388
Ago2	11.46	38.35	3.34642234	Acox3	67.38	19.81	0.29400416
Bola1	4.95	16.42	3.31717172	Psmb1	41.41	12.16	0.29364888
Ctps2	27.27	89.29	3.2742941	Snx8	79	23.17	0.29329114
Wasf2	15.59	51	3.27132777	Plod2	17.89	5.21	0.29122415
Pf4	29.49	96.46	3.2709393	Glipr2	29.36	8.53	0.29053134
Aip	11.27	36.78	3.2635315	Pcbp1	22	6.32	0.28727273
Mcm3	15.42	50.16	3.25291829	Pak2	33.64	9.66	0.28715815
Nap1l4	7.12	23.1	3.24438202	Cct2	11.19	3.21	0.28686327
Ogdh	6.75	21.89	3.24296296	Psmd2	12.53	3.59	0.28651237
Tkfc	5.13	16.57	3.23001949	Nceh1	105.39	30.14	0.28598539
Smu1	6.39	20.63	3.228482	Atg16l1	41.44	11.79	0.28450772
Sdhd	2.5	8.06	3.224	Ndufa13	50.51	14.35	0.28410216
Clpx	27.53	88.62	3.21903378	Zfp706	22.26	6.31	0.2834681
St13	3.94	12.68	3.21827411	Sorcs2	38.1	10.78	0.28293963
Gapvd1	30.19	96.22	3.18714806	Cep162	15.16	4.28	0.2823219
Anapc4	14.09	44.89	3.18594748	Ppt2	45.73	12.89	0.28187186
Arfgap1	5.03	15.99	3.17892644	Rps29	36.38	10.24	0.28147334
Pdlim5	19.33	61.34	3.17330574	Itih4	49.15	13.83	0.28138352
Suclg1	1.58	5	3.16455696	Lamtor5	20.38	5.73	0.281158
Swi5	12.97	41	3.16114109	Cpne2	58.09	16.29	0.28042692
Cnot9	39.77	125.66	3.15966809	Ikbip	35.34	9.9	0.28013582
Ckap4	1.95	6.16	3.15897436	Ift88	64.31	18.01	0.28004976
Etv6	5.74	18.02	3.13937282	Dpysl3	35.52	9.93	0.27956081

Capza1	5.49	17.15	3.12386157	Hnrnpa0	51.36	14.35	0.27940031
Tiprl	9.8	30.57	3.11938776	Akr7a2	81.66	22.81	0.27932892
Sap18b	15.1	47.09	3.11854305	Cuta	78.08	21.81	0.27932889
Cda	7.83	24.24	3.09578544	Dnajb4	62.91	17.55	0.27896996
Ppt1	5.93	18.18	3.06576728	Glrx5	44.81	12.48	0.27850926
Dnajb1	9.69	29.6	3.05469556	Poglut1	87.39	24.28	0.27783499
Wdr11	0.76	2.32	3.05263158	Lima1	50.95	14.15	0.27772326
Gnb1	7.87	24.02	3.05209657	Kyat3	14.08	3.9	0.27698864
Tpst2	10.64	32.45	3.04981203	Ctsd	11.65	3.21	0.27553648
Pon2	7.64	23.28	3.04712042	H2az2	53.22	14.62	0.27470876
Srsf9	25.79	78.48	3.04303994	Xpnpep1	34.09	9.36	0.27456732
Ccdc80	30.15	91.72	3.04212272	Lmcd1	75.31	20.61	0.27366884
Man1a2	5.57	16.92	3.03770197	Bud31	128.07	34.9	0.27250722
Atp6v1e1	18.87	57.24	3.03338633	Bicdl1	56.06	15.23	0.27167321
Anxa3	17.22	52.07	3.02380952	Itga1	35.06	9.52	0.27153451
Sart3	23.76	71.71	3.01809764	Dynll1	20.95	5.68	0.27112172
Nasp	7.08	21.36	3.01694915	Tmx3	103.95	28.16	0.27089947
Srrt	20.39	61.4	3.01128004	Oxr1	12.04	3.26	0.27076412
Litaf	9.52	28.63	3.00735294	Ndufa12	85.94	23.17	0.2696067
Rpsa	4.35	13.08	3.00689655	Timm50	28.32	7.63	0.2694209
Myl12b	3.72	11.12	2.98924731	Ptgr3	36.96	9.95	0.26920996
Rps9	5.79	17.3	2.98791019	Kdm3b	24	6.46	0.26916667
Stam2	17.36	51.82	2.98502304	Amer1	16.05	4.31	0.26853583
Psma5	2.09	6.23	2.98086124	Dbnl	66.7	17.91	0.26851574
Mrps18b	5	14.88	2.976	Acot13	81.17	21.79	0.26844893
Eif3j1	13.59	40.43	2.9749816	Nipsnap1	21.3	5.71	0.26807512
Scaf4	19.27	57.32	2.97457187	Pacs1	47.23	12.64	0.26762651
Mapk14	20.95	62.05	2.96181384	Mrps7	101.01	27.01	0.26739927
Dcaf13	14.59	43.17	2.95887594	Samm50	32.88	8.79	0.26733577
Rap2a	28.13	82.96	2.94916459	Pex5	27.35	7.31	0.26727605
Crtap	7.85	23.1	2.94267516	Tia1	18.03	4.79	0.26566833
Ap2s1	41.63	122.47	2.94186884	Eea1	8.33	2.21	0.26530612
Suclg2	7.3	21.43	2.93561644	Acsl4	23.59	6.24	0.26451886
Rps13	6.12	17.93	2.92973856	Myl6	24.59	6.48	0.26352176
Nt5dc2	21.15	61.93	2.92813239	Stx5a	92.86	24.47	0.26351497
Rtcb	10.27	30.01	2.92210321	Akr1e1	94.26	24.83	0.26342033
Cyb5r3	7.26	21.11	2.9077135	Gpr39	25.37	6.68	0.26330311
Get3	15.31	44.32	2.89483997	Smg8	35.41	9.3	0.26263767
Cox4i1	14.13	40.85	2.89101203	Ptms	58.89	15.46	0.26252335
Uba2	13.76	39.65	2.8815407	Apip	42.82	11.23	0.26226063
Thbs1	31.7	91	2.87066246	Phf14	60.49	15.86	0.2621921
Zfp326	20.59	59.06	2.86838271	Stt3a	40.15	10.5	0.2615193
Myof	2.39	6.84	2.86192469	Galk2	75.92	19.78	0.26053741
Prpf6	37.87	108.23	2.85793504	Fhl1	131.05	34.14	0.26051126
Tmem63a	4.24	12.04	2.83962264	Ostc	70.48	18.36	0.26049943
Pofut2	12.82	36.35	2.83541342	Sec24a	93.33	24.25	0.25983071
Wipf1	9.19	26	2.82916213	Asph	8.92	2.31	0.25896861
Usp47	8.4	23.7	2.82142857	Lsm8	37.94	9.82	0.25882973

Hgh1	17.46	49.03	2.80813288
Frg1	19.81	55.55	2.80413932
Stat1	13.29	37.21	2.79984951
Arfip1	0.78	2.18	2.79487179
Ubqln2	1.26	3.52	2.79365079
Sumf2	37.72	105.35	2.79294804
Lyar	23.73	66.07	2.784239359
Dhx58	18.56	51.55	2.77747845
Clptm1	8.6	23.84	2.77209302
Gpd2	20.56	56.95	2.76994163
Pdcd6	4.9	13.54	2.76326531
Mecp2	19.06	52.5	2.7544596
Farsb	11.59	31.81	2.74460742
Arhgef40	21.96	60.26	2.74408015
Gvin1	7.09	19.43	2.74047955
Pmvk	38.84	106.23	2.73506694
Cog1	7.14	19.46	2.7254902
Ifi204	9.99	27.2	2.72272272
Thbs1	31.02	84.22	2.71502257
Nme1nme2	2.24	6.06	2.70535714
C1qb	17.88	48.35	2.7041387
Eps15l1	4.82	13.03	2.7033195
Plp2	15.33	41.36	2.69797782
Gpx8	7.4	19.93	2.69324324
Hadhb	14.28	38.42	2.69047619
Hibch	13.38	35.97	2.68834081
Arl6ip5	6.27	16.84	2.68580542
Hnrnpab	16.58	44.5	2.68395657
Acbd5	35.18	94.29	2.68021603
Msn	7.09	18.97	2.67559944
Hk1	8.67	23.19	2.67474048
Rpf2	19.24	51.09	2.65540541
Cspg4	15	39.74	2.64933333
Skap2	12.32	32.56	2.64285714
Itga11	44.96	118.59	2.63767794
Acat2	13.5	35.59	2.6362963
Hnrnpdl	25.48	67.17	2.63618524
Surf4	8.05	21.15	2.62732919
Erc1	10.02	26.29	2.6237525
Cfl2	31.66	83.04	2.62286797
Hpcal1	1.68	4.39	2.61309524
Ugdh	12.11	31.62	2.61106524
Aatf	44.29	115.36	2.60465116
Dus3l	17.72	46.11	2.60214447
Slc37a2	14.78	38.4	2.59810555
H2-D1	13.21	34.22	2.59046177
Cap1	19.63	50.84	2.5899134
Ago1	4.9	12.69	2.58979592

Pfdn1	39.57	10.22	0.25827647
Zeb2	39.13	10.08	0.25760286
Copz2	40.24	10.36	0.25745527
Cdc73	133.7	34.36	0.25699327
Cggbp1	56.43	14.48	0.2566011
Adrm1	23.28	5.96	0.25601375
Sp100	75.32	19.26	0.25570898
Lpcat3	33.17	8.48	0.2556527
Sil1	104.82	26.78	0.25548559
Hspb1	33.03	8.42	0.25491977
Vma21	23.45	5.95	0.25373134
Gcat	82.61	20.96	0.25372231
Pura	44.74	11.26	0.25167635
Myo1g	44.71	11.23	0.25117423
Slc27a4	39.56	9.9	0.25025278
Ddx39a	127.35	31.86	0.25017668
Ccdc47	117.44	29.21	0.24872275
Mov10	61.17	15.17	0.24799738
Pla2g4a	38.58	9.54	0.24727838
Snrpe	31.26	7.7	0.24632118
Ints1	14.19	3.49	0.24594785
Cog6	36.48	8.97	0.24588816
Sec24d	46.89	11.52	0.24568138
Fhl2	17.87	4.39	0.24566312
Myh10	32.54	7.98	0.24523663
Nomo1	17.43	4.26	0.2444062
Lgmn	83.95	20.49	0.24407385
Tagln2	33.09	8.07	0.24388033
Kpna4	49.74	12.08	0.24286289
Ppme1	38.39	9.21	0.23990623
Gorasp2	29.06	6.95	0.23916036
Nup54	46.71	11.16	0.238921
Vps26a	60.27	14.34	0.23792932
Strip1	70.44	16.62	0.23594549
Chst14	23.53	5.5	0.23374416
Dctn5	68.91	16.1	0.23363808
Srsf4	91.54	21.37	0.23344986
Idh2	45.77	10.65	0.23268516
Rheb	26.97	6.25	0.23173897
Sh3gl1	47.79	11.04	0.23101067
Dnajb11	56.69	13.02	0.22967014
Stat3	34.45	7.91	0.229608128
Ermp1	54.68	12.5	0.22860278
Cnpy4	31.06	7.09	0.22826787
Cars1	49.9	11.38	0.22805611
Vbp1	12.09	2.75	0.22746071
Uqcrfs1	53.77	12.19	0.22670634
Dnajc8	43.26	9.8	0.22653722

Gpx7	10.28	26.61	2.5885214
Tmem258	2.34	6.04	2.58119658
Grk2	18.21	46.97	2.579352
Slc39a7	45.16	116.31	2.5755093
Vwa8	13.5	34.46	2.55259259
Ilkap	4.57	11.66	2.55142232
Hsd17b11	37.61	95.46	2.53815475
Gale	45.5	115.17	2.53120879
Ttn	8.55	21.61	2.52748538
Sec13	11.76	29.67	2.52295918
Coro1b	15.28	38.28	2.5052356
Mapre1	7.07	17.69	2.50212164
Ptk7	7.5	18.75	2.5
Txndc9	34.05	85.05	2.49779736
Acp2	13.57	33.83	2.49299926
Specc1l	38.49	95.91	2.49181606
Ppa1	13.63	33.91	2.48789435
Ctcf	23.31	57.92	2.48477048
Ctsz	8.89	21.95	2.46906637
Nrbf2	13.27	32.71	2.46495855
Impa1	20.17	49.65	2.4615766
Aak1	14.44	35.49	2.45775623
Mapk3	19.09	46.87	2.45521215
Cpsf6	8.81	21.56	2.44721907
Ephx1	22.57	55.04	2.43863536
Eif4a1	9.97	24.27	2.43430291
Nup43	8.03	19.49	2.42714819
Ddx39b	4.11	9.93	2.41605839
Sirt3	14.8	35.65	2.40878378
Sppl2a	14.32	34.49	2.40851955
Pcm1	6.34	15.27	2.40851735
Gng12	27.28	65.57	2.40359238
Scamp3	16.57	39.78	2.4007242
Sarnp	14.59	34.96	2.39616175
Sf3b3	24.25	58.07	2.39463918
Mgmt	18.73	44.84	2.39402029
Casp1	23.68	56.69	2.39400338
Etfb	5.12	12.25	2.39257813
env	19.49	46.62	2.3919959
Cpsf4	8.83	21.12	2.39184598
Poldip3	23.81	56.89	2.38933221
Pin1	34.01	81.26	2.38929727
Cdh3	30.22	71.91	2.37954997
Hnrnpl	11.78	28	2.37691002
Acot9	21.64	51.1	2.36136784
Ublcp1	14.81	34.97	2.3612424
Myo18a	12	28.28	2.35666667
Pacsin3	32.16	75.77	2.35603234

Tpd52	112.98	25.52	0.22588069
Uqcrb	28.3	6.38	0.2254417
Echs1	35.97	8.1	0.22518766
Ctsb	36.12	8.13	0.22508306
Sphk2	48.82	10.98	0.22490782
Slmap	73.14	16.42	0.22450096
Arf6	52.9	11.86	0.2241966
Jph2	7.63	1.71	0.22411533
Lyn	27.42	6.12	0.22319475
Etfdh	24.24	5.41	0.22318482
Hnrnpul2	21.07	4.7	0.22306597
Psmc3	4.44	0.99	0.22297297
Wdr1	15.72	3.5	0.22264631
Rab14	80.25	17.78	0.22155763
Fzd1	56.31	12.42	0.22056473
Lsm5	81.87	18.04	0.22034933
S100a10	39.69	8.71	0.21945074
Eci2	25.83	5.66	0.21912505
Ppp1cc	71	15.53	0.21873239
Fnbp4	40.69	8.87	0.21798968
Kank2	33.79	7.35	0.21751998
Alpl	42.19	9.17	0.21735008
Tgfb3	45.1	9.78	0.216851441
Csgalnact1	53.98	11.67	0.21619118
Acad8	60.46	13.05	0.21584519
Thy1	28	5.99	0.21392857
Adgre1	34.11	7.21	0.21137496
Acta2	23.15	4.89	0.2112311
Thbs4	47.51	10.02	0.21090297
Pkn2	17.16	3.61	0.21037296
Ttc28	57.48	12.03	0.20929019
Srgap2	31.79	6.61	0.20792702
Nsdhl	90.26	18.76	0.20784401
Arpc5l	20.38	4.23	0.20755643
Vdac3	121.67	25.03	0.20572039
Hs2st1	38.66	7.95	0.2056389
Gstm1	15.76	3.24	0.20558376
Cmpk2	26.13	5.33	0.2039801
Nploc4	24.49	4.98	0.20334831
Hck	52.77	10.72	0.20314573
Slc25a1	44.48	8.95	0.20121403
Mnt	42.32	8.5	0.20085066
Anxa2	5.31	1.06	0.19962335
Sh3kbp1	23.96	4.78	0.19949917
Emc10	30.24	6.01	0.19874339
Rps25	28.11	5.57	0.19815012
Rasa1	120.2	23.69	0.19708819
Chp1	65.26	12.84	0.19675146

Prmt1	21.23	49.76	2.34385304
Slc30a7	16.21	37.97	2.34238125
Zranb2	24.73	57.66	2.33158108
Rps17	7.38	17.2	2.33062331
Atp6v1a	16.01	37.26	2.32729544
Ormdl2	17.99	41.85	2.32629238
Mars1	29.5	68.56	2.3240678
Ogt	24.74	57.36	2.31851253
Ndufs1	11.91	27.61	2.31821998
Dock8	32.9	76.1	2.31306991
Atp5mj	10.32	23.87	2.3129845
Ubr4	2.37	5.48	2.31223629
Uchl5	15.13	34.96	2.31064111
Hsp90aa1	10.12	23.36	2.3083004
Ddah1	11.22	25.82	2.30124777
Tom1	33.06	75.92	2.29643073
Septin11	2.97	6.82	2.2962963
Plxna1	11.2	25.63	2.28839286
Lmna	11.05	25.23	2.28325792
Aldh16a1	15.98	36.42	2.27909887
Txndc17	17.95	40.71	2.26796657
Map1b	20.08	45.5	2.26593625
Alyref	12.67	28.69	2.2644041
Ugt1a7	27.26	61.58	2.25898753
Cdc5l	24.23	54.69	2.25711927
Tssc4	9.34	20.97	2.24518201
Phldb1	20.49	46	2.24499756
Dhrs7b	13.84	30.99	2.23916185
Papss1	15.27	33.85	2.2167649
Larp1	19.81	43.86	2.21403332
Mcm7	9.94	21.99	2.21227364
Cdkal1	55.9	123.42	2.2078712
Rcn1	11.31	24.97	2.20778073
Pdk3	11.9	26.26	2.20672269
Ube2n	3.97	8.76	2.20654912
Psmb6	4.13	9.1	2.20338983
Golga7	26.77	58.96	2.20246545
Yars1	11.7	25.72	2.1982906
Chmp4b	18.78	41.22	2.19488818
Ttll12	6.43	14.09	2.19129082
Ppig	21.05	46.09	2.18954869
Aldoa	19.75	43.19	2.18683544
Isyna1	13.56	29.64	2.18584071
Rps18	16.6	36.17	2.17891566
Ostf1	19.53	42.55	2.17869944
Sec22b	1.96	4.27	2.17857143
Smad3	6.27	13.6	2.16905901
Slc16a3	6.65	14.39	2.16390977

Usp4	64.42	12.65	0.19636759
Abcd1	65.91	12.89	0.19556972
Comt	30.27	5.88	0.19425173
Acadl	11.99	2.32	0.19349458
Myg1	40.94	7.91	0.19320957
Rars1	23.09	4.46	0.19315721
Dda1	21.4	4.13	0.19299065
Prrc2a	17.78	3.43	0.19291339
Tradd	92.55	17.78	0.19211237
Prpf40a	8.8	1.69	0.19204545
Gak	40.88	7.83	0.1915362
Mvk	112.98	21.57	0.19091875
Lamp1	81.96	15.59	0.19021474
Rps6	55.06	10.46	0.18997457
Ctsc	58.22	11.02	0.18928203
Arap1	91.99	17.38	0.18893358
Hp1bp3	11.14	2.09	0.18761221
Rpl23a	33.54	6.28	0.18723912
Cast	64.18	11.97	0.1865067
Rab11b	2.04	0.38	0.18627451
Thoc3	101.89	18.94	0.18588674
Itsn1	50.05	9.26	0.18501499
Tor1aip2	43.3	8.01	0.18498845
Anxa8	71.25	13.17	0.18484211
Naa25	53.02	9.79	0.1846473
Tmc5	17.15	3.16	0.18425656
Myo1f	80.59	14.74	0.1829011
Cnot1	44.68	8.14	0.18218442
Eif4a3	83.13	15.06	0.18116204
Pgrmc2	29.37	5.32	0.18113721
Tm9sf3	38.53	6.96	0.18063846
Plekho2	24.78	4.43	0.1787732
Tbc1d2b	38.42	6.85	0.17829256
Gdi1	52.56	9.37	0.17827245
Fam124b	28.66	5.09	0.17759944
Pdcd11	102.73	18.16	0.17677407
Ctnnd1	27.91	4.93	0.1766392
Nsf	29.21	5.13	0.17562479
Lig1	61.01	10.64	0.17439764
Nfu1	41.83	7.22	0.17260339
Prep	41.96	7.19	0.17135367
Prkar2b	25.08	4.29	0.17105263
P2rx4	29.94	5.12	0.17100868
Eif5b	23.93	4.08	0.17049728
Man2a1	68.75	11.7	0.17018182
Septin6	43.39	7.36	0.16962434
Macroh2a1	33.2	5.62	0.16927711
Endog	39.41	6.64	0.16848516

Commd8	7.83	16.9	2.15836526
Adss2	7.82	16.81	2.14961637
Esyt1	8.61	18.5	2.14866434
Rps7	22.26	47.79	2.14690027
Cavin1	6.24	13.36	2.14102564
Prelp	27.41	58.63	2.13900036
Hnrnph1	21.43	45.82	2.13812413
Fstl1	8.3	17.7	2.13253012
Mybbp1a	18.82	40.05	2.12805526
P4ha2	14.69	31.21	2.12457454
Cd2bp2	19.68	41.77	2.12245935
Sri	10.97	23.28	2.12215132
Ddt	32.89	69.55	2.11462451
Apex1	22.58	47.61	2.1085031
Unc45a	22.84	48.04	2.1033275
Nudt9	34.34	72.12	2.10017472
Cdk4	18.66	39.05	2.09271168
Ppp1r12a	21.9	45.81	2.09178082
Clasp2	12.52	26.16	2.08945687
Stom	5.94	12.39	2.08585859
Bccip	14.64	30.52	2.08469945
Ube2g1	16.13	33.5	2.07687539
Por	30.84	64.03	2.07619974
Sh3glb2	39.38	81.28	2.06399187
Dync1h1	4.15	8.55	2.06024096
Eif4ebp1	2.16	4.45	2.06018519
Atp6v0d1	6.82	14.04	2.05865103
Aoc3	16.03	32.97	2.05676856
Pnpt1	44.9	92.21	2.05367483
P4ha1	9.64	19.79	2.05290456
Hk3	32.89	67.42	2.04986318
Sf3a1	16.55	33.92	2.04954683
Lamb1	42.87	87.84	2.0489853
Epdr1	17.18	35.19	2.04831199
Sgpl1	8.73	17.87	2.04696449
Ifitm3	18.55	37.97	2.04690027
rps14	17.09	34.98	2.046811
Pdcd2	38.51	78.81	2.04648143
Maoa	5.72	11.68	2.04195804
Trex1	48.85	99.71	2.04114637
Cpne1	11.72	23.89	2.0383959
Vta1	32.73	66.64	2.03605255
Fcgr2b	53.59	108.99	2.03377496
Cd80	30.02	61	2.03197868
Wdr18	14.66	29.72	2.02728513
Kpna3	1.14	2.31	2.02631579
Pdia6	12.26	24.84	2.02610114
CD14	17.01	34.46	2.02586714

Psmb2	71.76	12.09	0.16847826
Igf2bp2	39.18	6.48	0.16539051
Mcm4	92.73	15.26	0.16456379
Xpo1	52.23	8.59	0.16446487
Nup214	98.64	16.22	0.16443633
Nedd8	32.13	5.28	0.1643324
Itgb2	5.89	0.96	0.162988115
H1f0	65.22	10.63	0.16298681
Srsf11	69.93	11.38	0.16273416
Arfgap3	27.08	4.4	0.16248154
Tpm2	34.26	5.55	0.1619965
Synj2	34.67	5.61	0.16181136
Ndufv1	10.57	1.71	0.16177862
Cpsf2	23.08	3.7	0.16031196
Rps10	59.33	9.5	0.16012136
Mpdu1	52.66	8.43	0.16008355
Sbno1	64.64	10.34	0.15996287
Borcs6	20.77	3.32	0.15984593
Tmem167a	11.42	1.82	0.15936953
Sh3bgrl	82.45	13.13	0.15924803
Ddb1	40.09	6.36	0.15864305
Mccc2	17.61	2.79	0.15843271
Cdk2	29.45	4.66	0.158234295
Etfa	27.81	4.38	0.1574973
Pla2g15	27.62	4.34	0.15713251
Necap2	26.15	4.09	0.15640535
Napa	9.57	1.49	0.15569488
Ube2m	27.28	4.22	0.15469208
Ptgis	71.35	10.98	0.15388928
Hs1bp3	32.91	5.05	0.1534488
Capn2	15.52	2.37	0.15270619
Ptpa	31.2	4.74	0.15192308
Slc2a1	31.34	4.74	0.15124442
Psma4	70.77	10.69	0.15105271
Atg4b	46.68	6.99	0.14974293
Lmnb1	13.32	1.99	0.1493994
Slc26a3	49.54	7.37	0.14876867
Ahnak2	16.48	2.44	0.14805825
Rtraf	137.83	20.35	0.14764565
Lap3	25.48	3.76	0.14756672
Nosip	91.26	13.4	0.14683322
Cdh11	52.53	7.65	0.14563107
Pfkp	111.18	16.15	0.14525994
Ano6	25.06	3.63	0.14485235
Map7d1	24.74	3.57	0.14430073
Lgalsl	32.26	4.63	0.14352139
Vps13c	47.63	6.77	0.14213731
Nol3	92.6	13.14	0.14190065

Atg7	41.32	83.55	2.02202323
Rab6a	25.38	51.31	2.02167061
Psmd13	1.48	2.99	2.02027027
Irf2bp2	24.19	48.85	2.01942952
Dlgap4	29.94	60.36	2.01603206

Snx18	39.86	5.64	0.14149523
Rpl34	12.94	1.83	0.14142195
Bcl2l1	11.81	1.67	0.14140559
Ankrd17	11.25	1.59	0.14133333
Rfc3	27.4	3.87	0.14124088
Arhgap1	12.13	1.71	0.14097279
Actg2	67.56	9.44	0.13972765
Ncl	94.71	13.23	0.13968958
Upf2	56.48	7.72	0.13668555
Tsfm	46.42	6.33	0.13636364
Pdlim7	37.42	5.09	0.13602352
Purb	71.05	9.61	0.13525686
Nisch	30.11	4.06	0.13483892
Adk	26.27	3.53	0.13437381
Ddx23	109.21	14.65	0.13414522
Aifm1	15.17	2.03	0.13381674
Ndufa8	28.5	3.81	0.13368421
Chordc1	73.3	9.77	0.13328786
Gtpbp1	57.72	7.69	0.13322938
Tubg1	76.94	10.2	0.13257083
Lnpep	31.24	4.13	0.1322023
Acbd3	67.13	8.8	0.13108893
Pgp	23.03	3.01	0.13069909
Ascc1	47.21	6.16	0.13048083
Rps5	30	3.9	0.13
S100a9	137.44	17.83	0.12972934
Lsm3	71.15	9.21	0.12944483
Cbx3	65.73	8.5	0.1293169
Cdr2l	25.43	3.28	0.12898152
Stx18	52.97	6.77	0.12780819
Ube2v1	41.84	5.32	0.12715105
Rab2a	12.3	1.56	0.12682927
Snw1	37.07	4.7	0.12678716
Hspa1b	28.7	3.63	0.12648084
Mrps27	54.63	6.83	0.12502288
Upf1	27.83	3.46	0.12432627
Timm17b	8.22	1.02	0.12408759
Nudt5	41.34	5.12	0.12385099
Ndufb8	59.83	7.41	0.12385091
Fkbp7	32.85	4.05	0.12328767
Lpcat2	122.82	15.08	0.12278131
Ak4	26.83	3.28	0.12225121
Strn4	105.12	12.85	0.12224125
Ap2a2	53.12	6.48	0.12198795
Lta4h	39.38	4.78	0.12138141
Ywhaq	35.97	4.36	0.12121212
Tomm22	71.86	8.7	0.12106874
Ppp6c	27.17	3.28	0.12072138

Slc35b2	24.4	2.93	0.12008197
Mia3	47.72	5.72	0.11986588
Uba1	6.24	0.74	0.11858974
Psmc5	67.71	8.02	0.11844632
Vkorc1l1	138.41	16.32	0.11791056
Lasp1	23.97	2.81	0.11722987
Rabgef1	30.83	3.59	0.11644502
Imp3	39.48	4.54	0.11499493
Ndufs3	42.25	4.83	0.11431953
Hnrnpc	21.83	2.48	0.11360513
Zc3h11a	17.53	1.99	0.11351968
Rasa4	61.4	6.92	0.11270358
Exosc4	35.47	3.98	0.1122075
Rpl37a	43.23	4.85	0.11219061
Tsn	38.6	4.33	0.11217617
Gmppa	121.38	13.54	0.1115505
Cxcl12	72.08	7.97	0.11057159
Rplp2	9.54	1.05	0.11006289
Pes1	28.33	3.09	0.10907166
Acaa2	39.64	4.32	0.10898083
Bcl10	52.51	5.69	0.10836031
Ndufb9	82.51	8.92	0.10810811
Dlst	5.84	0.63	0.10787671
Glrx3	19.9	2.12	0.10653266
Prdx5	12.6	1.34	0.10634921
Ap2m1	41.7	4.41	0.1057554
Soat1	125	13.1	0.1048
Trip11	13.05	1.36	0.10421456
Svil	39.65	4.09	0.10315259
Flnb	32.93	3.39	0.10294564
Mpp1	40.99	4.19	0.10222005
Pcare	43.74	4.47	0.10219479
Smad1	24.11	2.46	0.102032352
Ccdc115	9.16	0.93	0.10152838
Scarb2	55.39	5.59	0.10092074
Fcer1g	57.32	5.77	0.10066294
C1qa	47.78	4.77	0.09983257
Trpv2	45.51	4.54	0.09975829
Vps28	52.65	5.22	0.0991453
Gatd3	73.9	7.2	0.09742896
Adprs	13.88	1.35	0.09726225
Txnl1	5.56	0.54	0.0971223
Srek1	75.68	7.25	0.0957981
Ptk2	6.8	0.65	0.09558824
Tbcc	31.72	2.98	0.09394704
Zw10	70.19	6.48	0.09232084
Tcirg1	49.06	4.52	0.09213208
Supt5h	18.25	1.68	0.09205479

Timm13	59.87	5.5	0.09186571
Camk2g	29.78	2.73	0.09167226
Scfd2	113.84	10.43	0.09161982
Golga2	29.82	2.69	0.09020791
Bphl	59.12	5.25	0.08880244
Psmb3	35.72	3.17	0.0887458
Mpst	30.1	2.65	0.08803987
Psmc6	40.73	3.58	0.0878959
Prdx4	39.96	3.47	0.08683684
Tmx4	103.37	8.94	0.08648544
Lamtor1	53.85	4.56	0.08467967
Kif3a	19.73	1.67	0.08464268
Tuba1a	27.36	2.28	0.08333333
Uba6	51.85	4.32	0.08331726
Uqcrh	96.3	7.96	0.08265836
Mcrip1	36.07	2.98	0.08261713
Lmnb2	62.27	5.1	0.0819014
Gtf3c4	37.11	2.97	0.08003234
Cdk1	38.21	3.03	0.079298613
Cdkn2c	32.57	2.57	0.07890697
Ssr3	10.93	0.86	0.07868253
Mmut	128.25	10.08	0.07859649
Psmc1	9.81	0.77	0.07849134
Nudt21	35.96	2.82	0.07842047
Esyt2	52.6	4.1	0.07794677
Phb2	2.22	0.17	0.07657658
Akt1s1	40.18	3.05	0.07590841
Aldh6a1	5.99	0.45	0.07512521
Cpox	29.86	2.2	0.07367716
Ufd1	23.79	1.75	0.07356032
Skic8	19.2	1.39	0.07239583
C1galt1c1	29.57	2.11	0.0713561
Dcaf5	30.91	2.19	0.07085086
Nlrx1	114.34	8.1	0.07084135
Glrx	10.92	0.77	0.07051282
Nubp2	120.18	7.72	0.06423698
Hnrnph2	21.65	1.38	0.06374134
Vamp5	18.77	1.19	0.06339904
Stx7	33.24	2.09	0.06287605
Rab18	15.45	0.96	0.06213592
Pcyt1a	39.41	2.44	0.06191322
Fhod1	111.92	6.77	0.06048964
Nucb2	9.11	0.55	0.06037322
Myadm	29.02	1.75	0.06030324
Drg2	7.64	0.46	0.06020942
Galnt1	41.25	2.4	0.05818182
Ebp	47.79	2.73	0.05712492
Trappc3	65.62	3.72	0.05669003

Ifi35	44.29	2.47	0.0557688
Fbxl20	55.51	3.08	0.0554855
Polr2a	36.98	2.02	0.05462412
Timm8a1	45.61	2.46	0.05393554
Acadvl	42.98	2.29	0.0532806
Rabep1	30.49	1.58	0.05182027
Acsl5	45.66	2.36	0.05168638
Agpat1	14.72	0.76	0.05163043
Rab21	128.26	6.6	0.05145798
Enoph1	105.75	5.38	0.0508747
Ero1a	137.61	6.88	0.04999637
Cacul1	76.45	3.81	0.04983649
Gar1	31.93	1.55	0.04854369
Stk3	41.27	1.97	0.04773443
Pdlim1	9.51	0.45	0.04731861
Lox	48.11	2.27	0.04718354
Tmpo	16.45	0.75	0.04559271
Fech	40.52	1.84	0.04540967
Dpf2	4.22	0.19	0.0450237
Oxct1	18.79	0.84	0.04470463
Ripk1	21.51	0.93	0.0432357
Tppp3	21.96	0.93	0.04234973
Ik	10.13	0.42	0.04146101
Cdc37	19.82	0.8	0.04036327
Rpn1	22.04	0.88	0.0399274
Lims1	64.87	2.59	0.03992601
Cops8	27.05	1.05	0.03881701
Celf2	20.48	0.79	0.03857422
Stat2	137.92	5.32	0.03857309
Aga	11.44	0.42	0.03671329
Clns1a	62.37	2.21	0.0354337
Gabpa	58.71	2.06	0.03508772
Fcgr1	41.45	1.45	0.03498191
2900026A02Rik	15.18	0.53	0.03491436
Niban1	30.96	1.01	0.03262274
Rdx	50.16	1.62	0.03229665
Hmgb1	41.13	1.3	0.0316071
Slc25a13	119.11	3.74	0.03139955
Tmbim6	26.86	0.79	0.02941176
Pfdn4	64.07	1.86	0.02903075
mt-Co2	36.43	1.02	0.0279989
Lpcat1	71.89	1.99	0.02768118
Ndufb5	68.88	1.86	0.02700348
Ccdc22	81.55	2.05	0.02513795
Washc1	17.03	0.42	0.02466236
Ccdc102a	69.09	1.7	0.02460559
Rab5a	14.9	0.36	0.02416107
Vps18	97.56	2.34	0.02398524

Rab4b	58.53	1.35	0.02306509
Prpf19	32.26	0.71	0.02200868
Cept1	32.45	0.71	0.02187982
Atp13a1	12.53	0.27	0.02154828
Rac1	20.65	0.4	0.01937046
Setd7	83.2	1.51	0.01814904
Mcu	68.2	1.22	0.01788856
Ccnyl1	10.98	0.19	0.01730419
Pkm	40.72	0.7	0.01719057
Rhob	14.38	0.22	0.01529903
Nudc	52.3	0.79	0.01510516
Prps2	93.13	1.36	0.01460324
Nedd4	11.04	0.16	0.01449275
Zfp36l2	84.17	1.15	0.01366283
Aldh3a2	12.57	0.17	0.01352426
Bpnt2	31.57	0.39	0.0123535
Pxk	49.21	0.6	0.01219264
Notch2	66.96	0.81	0.012096774
Tubb5	34.84	0.39	0.01119403
Decr1	16.8	0.18	0.01071429
Canx	8.68	0.09	0.01036866
Tmem263	23.32	0.22	0.00943396
Tpm1	16.18	0.15	0.0092707
Rnf2	21.21	0.19	0.00895804
Gigyf2	93.12	0.76	0.00816151
FAM120A	16.41	0.1	0.00609385
Bckdhb	35.8	0.2	0.00558659
Gas1	39.54	0.17	0.00429944
Lsm4	38.62	0.14	0.00362506
Erlin2	89.21	0.31	0.00347495
Kif13b	27.9	0.08	0.00286738
Rap1b	7.42	0.02	0.00269542
Pisd	53.64	0.08	0.00149142
Cdc42ep4	119.83	0.15	0.00125177
Prpf31	47.54	0.04	0.0008414
Derl1	41.08	0.02	0.00048685
