# Supplementary material for: Decreased expression of Ly-1 antibody reactive clone (Lyar) triggers enhanced adipogenesis of bone marrow mesenchymal stromal cells in aged bone marrow
Source: PLoS One. 2026 May 27;21(5):e0349780. doi: 10.1371/journal.pone.0349780 (PMC13215539; doi:10.1371/journal.pone.0349780)
Supplement: S5 Table — (PDF) [file pone.0349780.s008.pdf]

#	Uniprot Ac.	Protein name
1994	P01631	Ig kappa chain V-II region 26-10
411	Q08288	Cell growth-regulating nucleolar protein
5191	O35253	Mothers against decapentaplegic homolog 7
4837	Q9CQA1	Trafficking protein particle complex subunit 5
2920	P28231	Gap junction beta-3 protein
5902	Q9Z0U0	Xenotropic and polytropic retrovirus receptor 1
5467	Q8CHG5	Apoptosis-resistant E3 ubiquitin protein ligase 1
2811	Q9QXA5	U6 snRNA-associated Sm-like protein LSm4
5906	Q8BJ63	AP-5 complex subunit mu-1
5437	Q9WVH3	Forkhead box protein O4
2209	Q99M54	Cell division cycle-associated protein 3
3452	Q8VE38	Oxidoreductase NAD-binding domain-containing protein 1
402	P97452	Ribosome biogenesis protein BOP1
5131	P16381	Putative ATP-dependent RNA helicase Pl10
2733	Q9CQW9	Interferon-induced transmembrane protein 3
468	Q8K3W3	Protein CASC3
5572	Q3TDE8	Zinc finger protein 691
4813	Q80VQ1	Leucine-rich repeat-containing protein 1
5984	Q8C033	Rho guanine nucleotide exchange factor 10
3621	Q3U1N2	Sterol regulatory element-binding protein 2
97	Q8VIG1	RE1-silencing transcription factor
4377	Q7TQK4	Exosome complex component RRP40
2993	Q3TFK5	G patch domain-containing protein 4
505	Q8R2N2	U3 small nucleolar RNA-associated protein 4 homolog
2477	Q8R344	Coiled-coil domain-containing protein 12
4704	Q8BWM0	Prostaglandin E synthase 2
538	Q810V0	U3 small nucleolar ribonucleoprotein protein MPP10
49	Q91WM3	U3 small nucleolar RNA-interacting protein 2
2131	Q9JHS9	Spliceosome-associated protein CWC15 homolog
5707	P07759	Serine protease inhibitor A3K
5557	Q9Z2B5	Eukaryotic translation initiation factor 2-alpha kinase 3
842	Q69ZQ2	Pre-mRNA-splicing factor ISY1 homolog
1808	Q9CZX5	PIN2/TERF1-interacting telomerase inhibitor 1
154	Q8R2M2	Deoxynucleotidyltransferase terminal-interacting protein 2
4721	Q9CPV5	Polyamine-modulated factor 1
1277	Q8BHB4	WD repeat-containing protein 3
260	Q6ZQL4	WD repeat-containing protein 43
43	Q8BU03	Periodic tryptophan protein 2 homolog
100	Q8BKS9	Pumilio homolog 3

4178	Q3UHI0	Serine-rich coiled-coil domain-containing protein 2
1078	Q8BGA5	KRR1 small subunit processome component homolog
629	P61327	Protein mago nashi homolog
629.1	P61327	Protein mago nashi homolog
629.2	Q9CQL1	Protein mago nashi homolog 2
101	Q8BJW5	Nucleolar protein 11
189	Q9Z0H1	WD repeat-containing protein 46
556	Q8VCY6	U3 small nucleolar RNA-associated protein 6 homolog
3689	Q499E6	Uncharacterized protein C1orf109 homolog
4701	Q8CHY3	Dymeclin
443	Q9R0U0	Serine/arginine-rich splicing factor 10
781	Q9CWZ3	RNA-binding protein 8A
81	Q921Y2	U3 small nucleolar ribonucleoprotein protein IMP3
432	Q5RJG1	Nucleolar protein 10
736	P56183	Ribosomal RNA processing protein 1 homolog A
766	Q9JHJ0	Tropomodulin-3
3297	Q9JKK7	Tropomodulin-2
1321	Q9JJA4	Ribosome biogenesis protein WDR12
3668	Q8BQR4	KAT8 regulatory NSL complex subunit 2
957	Q9JJT0	RNA 3'-terminal phosphate cyclase-like protein
4777	Q8C2L6	Transmembrane protein 161B
813	Q8JZX3	POC1 centriolar protein homolog A
4827	Q60855	Receptor-interacting serine/threonine-protein kinase 1
4899	Q9D7Y9	Protein SLX4IP
605	Q9CYX7	RRP15-like protein
147	Q8C4J7	Transducin beta-like protein 3
1729	Q8R149	BUD13 homolog
536	Q8K3Y3	Protein lin-28 homolog A
2902	Q3UGF1	WD repeat-containing protein 19
3506	Q9EPV8	Ubiquitin-like protein 5
4948	O35613	Death domain-associated protein 6
4563	Q99PN3	Tripartite motif-containing protein 26
1159	P62996	Transformer-2 protein homolog beta
3942	Q99MX0	Transketolase-like protein 1
585	Q8VHZ7	U3 small nucleolar ribonucleoprotein protein IMP4
4080	Q3U2U7	Methyltransferase-like protein 17, mitochondrial
362	Q91VC3	Eukaryotic initiation factor 4A-III
1206	Q5I043	Ubiquitin carboxyl-terminal hydrolase 28
1528	Q9D0T1	NHP2-like protein 1
2533	Q3TC93	HCLS1-binding protein 3

3136	Q8CIL4	Uncharacterized protein C1orf131 homolog
416	Q91VJ4	Serine/threonine-protein kinase 38
5813	Q9Z1L3	Death effector domain-containing protein
499	O35491	Dual specificity protein kinase CLK2
3117	Q9QYH6	Melanoma-associated antigen D1
3824	Q6PFR5	Transformer-2 protein homolog alpha
200	Q923D4	Splicing factor 3B subunit 5
5108	P98197	Probable phospholipid-transporting ATPase IH
695	Q9CXK8	60S ribosome subunit biogenesis protein NIP7 homolog
1328	Q921S7	39S ribosomal protein L37, mitochondrial
1534	Q9CZG3	COMM domain-containing protein 8
970	Q8BVY0	Ribosomal L1 domain-containing protein 1
1147	Q9D287	Pre-mRNA-splicing factor SPF27
2060	B1AY10	Transcriptional repressor NF-X1
1760	P57784	U2 small nuclear ribonucleoprotein A'
4410	A2AKG8	Focadhesin
1172	O35493	Dual specificity protein kinase CLK4
5767	Q8R3I2	Lysophospholipid acyltransferase 2
5472	Q8BHG2	CXXC motif containing zinc binding protein
812	Q8VCG3	WD repeat-containing protein 74
4090	Q8CFY5	Protoheme IX farnesyltransferase, mitochondrial
1782	Q6PDM2	Serine/arginine-rich splicing factor 1
5181	Q9EPA7	Nicotinamide/nicotinic acid mononucleotide adenylyltransferase
4291	Q9D2C2	Protein SAAL1
2168	Q6P7W2	SH3KBP1-binding protein 1
4390	Q9D0V8	Cyclin-dependent kinase 2-interacting protein
2595	Q8BPK2	Zinc finger CCHC domain-containing protein 3
1112	Q7TSG3	F-box only protein 5
487	Q9D1C9	Ribosomal RNA-processing protein 7 homolog A
1976	Q9ESN5	Centromere protein K
806	Q9DC48	Pre-mRNA-processing factor 17
818	Q5SSI6	U3 small nucleolar RNA-associated protein 18 homolog
5247	Q3U6K5	Spermatogenesis-associated protein 6
1283	Q8C5N3	Pre-mRNA-splicing factor CWC22 homolog
513	Q9WTI7	Unconventional myosin-Ic
57	Q8BTI8	Serine/arginine repetitive matrix protein 2
4141	Q6R891	Neurabin-2
69	Q99104	Unconventional myosin-Va
4895	Q9D1I2	Caspase recruitment domain-containing protein 19
782	Q8C7V3	U3 small nucleolar RNA-associated protein 15 homolog

2255	Q9QZH3	Peptidyl-prolyl cis-trans isomerase E
651	Q922K7	Probable 28S rRNA (cytosine-C(5))-methyltransferase
4227	P56695	Wolframin
4610	Q9JLB2	MAGUK p55 subfamily member 5
178	Q3U821	WD repeat-containing protein 75
520	Q3UKJ7	WD40 repeat-containing protein SMU1
4743	Q8R3W5	tRNA-splicing endonuclease subunit Sen15
1687	Q6PGH1	Protein BUD31 homolog
34	Q9JJ80	Ribosome production factor 2 homolog
2969	Q8C1E7	Transmembrane protein 120A
1417	Q3TKY6	Spliceosome-associated protein CWC27 homolog
162	Q640M1	U3 small nucleolar RNA-associated protein 14 homolog A
367	Q9D8S9	BolA-like protein 1
3563	Q08093	Calponin-2
4440	P07214	SPARC
4713	P62307	Small nuclear ribonucleoprotein F
1355	Q9EQ61	Pescadillo homolog
5325	P63002	TLE family member 5
155	P59708	Splicing factor 3B subunit 6
4640	Q91WD7	Kinesin-like protein KIF18A
4145	Q8BGT5	Alanine aminotransferase 2
4048	Q5IRJ6	Zinc transporter 9
1446	C0HKD8	Microfibrillar-associated protein 1A
1446.1	C0HKD8	Microfibrillar-associated protein 1A
1446.2	C0HKD9	Microfibrillar-associated protein 1B
3975	Q9DAJ4	WD repeat domain-containing protein 83
410	Q8BGS0	Protein MAK16 homolog
519	Q9D516	Coiled-coil domain-containing protein 130
4513	Q8BGE5	Fanconi anemia group M protein homolog
301	Q61382	TNF receptor-associated factor 4
4920	Q60795	Nuclear factor erythroid 2-related factor 2
3902	Q8C3I8	Protein HGH1 homolog
375	Q6NS46	Protein RRP5 homolog
4407	B1AZA5	Transmembrane protein 245
1258	Q8BIZ6	Smad nuclear-interacting protein 1
2048	P63154	Crooked neck-like protein 1
458	Q8CFQ3	RNA helicase aquarius
5533	A2A5K6	Zinc finger protein 335
1314	Q9CZT6	Protein CMSS1
5402	Q3U133	Zinc finger protein 746

5423	Q497H0	AN1-type zinc finger protein 3
4855	Q91YE3	Egl nine homolog 1
476	Q9CSN1	SNW domain-containing protein 1
5739	Q8K353	Cysteine-rich and transmembrane domain-containing protein 1
3388	Q60670	Serine/threonine-protein kinase SIK1
2392	Q9D0B0	Serine/arginine-rich splicing factor 9
364	Q99ME9	Nucleolar GTP-binding protein 1
3933	Q9CQF0	39S ribosomal protein L11, mitochondrial
2614	Q14B70	Highly divergent homeobox
29	Q99KP6	Pre-mRNA-processing factor 19
113	Q61879	Myosin-10
574	Q9D0R4	Probable ATP-dependent RNA helicase DDX56
52	Q6AW69	Cingulin-like protein 1
2698	P11260	LINE-1 retrotransposable element ORF1 protein
85	Q99PV0	Pre-mRNA-processing-splicing factor 8
250	O08810	116 kDa U5 small nuclear ribonucleoprotein component
588	O35691	Pinin
4331	Q9WTK7	Serine/threonine-protein kinase STK11
12	Q8K019	Bcl-2-associated transcription factor 1
2481	Q9Z204	Heterogeneous nuclear ribonucleoproteins C1/C2
181	P46735	Unconventional myosin-Ib
26	Q9ESX5	H/ACA ribonucleoprotein complex subunit DKC1
369	Q91VM5	RNA binding motif protein, X-linked-like-1
66	Q9DCA5	Ribosome biogenesis protein BRX1 homolog
5151	Q80YR6	DNA endonuclease RBBP8
5381	Q99KU0	Vacuole membrane protein 1
4184	Q1EG27	Myosin-IIIb
881	Q62377	U2 small nuclear ribonucleoprotein auxiliary factor 35 kDa subun
2974	Q8VCE2	GPN-loop GTPase 1
669	Q6DFW4	Nucleolar protein 58
590	Q921M3	Splicing factor 3B subunit 3
4601	Q8VCH8	UBX domain-containing protein 4
1014	Q6PE01	U5 small nuclear ribonucleoprotein 40 kDa protein
594	B2RQC2	Ubiquitin carboxyl-terminal hydrolase 42
5397	Q922R5	Serine/threonine-protein phosphatase 4 regulatory subunit 3B
966	Q8C0P7	E3 SUMO-protein ligase ZNF451
2699	Q9D8Y8	Inhibitor of growth protein 5
4170	Q505B7	Protein archease
5076	Q3UFS4	G patch domain-containing protein 11
1940	Q8BHS3	Pre-mRNA-splicing factor RBM22

2944	Q61072	Disintegrin and metalloproteinase domain-containing protein 9
5626	Q68FE6	Rho family-interacting cell polarization regulator 1
151	Q6P4T2	U5 small nuclear ribonucleoprotein 200 kDa helicase
5918	Q3UES3	Poly [ADP-ribose] polymerase tankyrase-2
1648	Q9CZ91	Serum response factor-binding protein 1
755	Q9D6Z1	Nucleolar protein 56
2843	Q9QZH6	Evolutionarily conserved signaling intermediate in Toll pathway,
4484	Q8QZS3	Folliculin
323	E9Q286	Little elongation complex subunit 1
5025	Q8R3L8	Cyclin-dependent kinase 8
2800	Q6ZQ12	Ninein-like protein
5416	Q3U0S6	Ras-interacting protein 1
4588	Q9CQZ1	Heat shock factor-binding protein 1
566	Q9Z1M8	Protein Red
917	Q9CQI7	U2 small nuclear ribonucleoprotein B"
3712	Q9QUK3	Protein CLN8
4578	P58544	BTB/POZ domain-containing protein 1
5078	Q921D4	Mediator of RNA polymerase II transcription subunit 6
253	Q8BHY2	Nucleolar complex protein 4 homolog
3706	Q9D0K1	Peroxisomal membrane protein PEX13
5535	P97431	Interferon regulatory factor 6
3431	Q920D3	Mediator of RNA polymerase II transcription subunit 28
167	Q99NB9	Splicing factor 3B subunit 1
2685	Q9CYR6	Phosphoacetylglucosamine mutase
2828	Q9JJ94	Sjoegren syndrome nuclear autoantigen 1 homolog
2506	Q8BY02	NF-kappa-B-repressing factor
767	Q8C7U1	NEDD4-binding protein 3
1832	Q9EQQ2	Protein YIPF5
93	Q922V4	Pleiotropic regulator 1
5802	Q9QVP4	Myosin regulatory light chain 2, atrial isoform
5251	Q9JIQ3	Diablo homolog, mitochondrial
2680	Q91VP7	Transmembrane protein 101
4727	E9Q8D0	DnaJ homolog subfamily C member 21
1033	Q80Y44	Probable ATP-dependent RNA helicase DDX10
5428	D3Z6H8	S-adenosylmethionine decarboxylase proenzyme 2
5428.1	D3Z6H8	S-adenosylmethionine decarboxylase proenzyme 2
5428.2	P0DMN7	S-adenosylmethionine decarboxylase proenzyme 1
646	E9Q634	Unconventional myosin-Ie
5468	Q8BPE4	Transmembrane protein 177
264	Q9QX47	Protein SON

1090	Q8QZY9	Splicing factor 3B subunit 4
1284	Q8JZX4	Splicing factor 45
4433	Q61048	WW domain-binding protein 4
4790	Q64705	Upstream stimulatory factor 2
2401	Q91VW9	Zinc finger protein with KRAB and SCAN domains 3
156	Q3THE2	Myosin regulatory light chain 12B
4575	Q9JK83	Partitioning defective 6 homolog beta
1264	Q9QYL7	Activator of basal transcription 1
1327	Q69ZH9	Rho GTPase-activating protein 23
4365	Q8C1Z7	Bardet-Biedl syndrome 4 protein homolog
5579	Q3UDR8	Protein YIPF3
5716	Q9D1F4	Proline-rich AKT1 substrate 1
5230	E9Q4N7	AT-rich interactive domain-containing protein 1B
2479	P47753	F-actin-capping protein subunit alpha-1
4511	Q9D7E3	Esterase OVCA2
3919	Q8R0Z5	Mitoferrin-2
1364	P47757	F-actin-capping protein subunit beta
287	Q8R3C6	Probable RNA-binding protein 19
2150	Q9CQV7	Mitochondrial import inner membrane translocase subunit TIM1
4900	Q9DAA6	Exosome complex component CSL4
386	O55135	Eukaryotic translation initiation factor 6
4674	Q9JKV5	Secretory carrier-associated membrane protein 4
1692	Q6PGL7	WASH complex subunit 2
5203	Q8BMI0	F-box only protein 38
3683	Q91XC9	Peroxisomal membrane protein PEX16
4406	Q91YN9	BAG family molecular chaperone regulator 2
5912	Q99LJ0	CTTNBP2 N-terminal-like protein
5389	Q8BX07	Carboxy-terminal domain RNA polymerase II polypeptide A small
4994	Q8BH55	Threonine synthase-like 1
4336	Q8CAS9	Protein mono-ADP-ribosyltransferase PARP9
3882	B2RX88	Centrosome and spindle pole associated protein 1
3345	Q9CZ57	5-methylcytosine rRNA methyltransferase NSUN4
748	Q9D554	Splicing factor 3A subunit 3
2903	P49962	Signal recognition particle 9 kDa protein
5375	Q8JZV7	N-acetylglucosamine-6-phosphate deacetylase
1761	Q3V1V3	ESF1 homolog
3611	Q921I9	Exosome complex component RRP41
5518	Q66T02	Pleckstrin homology domain-containing family G member 5
192	Q9CPS7	RNA-binding protein PNO1
4316	Q8VC31	Coiled-coil domain-containing protein 9

1089	Q60605	Myosin light polypeptide 6
5133	Q61321	Homeobox protein SIX4
3799	Q9CRA8	Exosome complex component RRP46
3339	Q922M5	Cell division cycle-associated 7-like protein
5498	Q8BSM7	Large neutral amino acids transporter small subunit 3
5281	Q9CZ82	Mediator of RNA polymerase II transcription subunit 18
2603	Q91X78	Erlin-1
3793	Q8BW70	Ubiquitin carboxyl-terminal hydrolase 38
2787	Q9JJR5	A-kinase-interacting protein 1
5403	Q61884	Meiosis-specific nuclear structural protein 1
165	Q922P9	Putative oxidoreductase GLYR1
1877	Q8R1U1	Conserved oligomeric Golgi complex subunit 4
5190	Q922M7	Ashwin
2618	Q9CR50	RING finger and CHY zinc finger domain-containing protein 1
4253	Q8BFZ3	Beta-actin-like protein 2
398	Q62093	Serine/arginine-rich splicing factor 2
401	Q9D0W5	Peptidyl-prolyl cis-trans isomerase-like 1
2668	Q9CY66	H/ACA ribonucleoprotein complex subunit 1
1022	Q9DCK4	Zinc finger protein 414
5978	Q80W54	CAAX prenyl protease 1 homolog
3769	Q7TSV4	Phosphoglucomutase-2
239	Q5DTX6	Junctional protein associated with coronary artery disease
5132	Q56A08	G-patch domain and KOW motifs-containing protein
3370	Q9DCD5	Tight junction-associated protein 1
537	Q6A068	Cell division cycle 5-like protein
1146	Q9CRB2	H/ACA ribonucleoprotein complex subunit 2
54	Q9D0I8	mRNA turnover protein 4 homolog
823	Q8BX10	Serine/threonine-protein phosphatase PGAM5, mitochondrial
3183	Q3TWW8	Serine/arginine-rich splicing factor 6
5187	O88597	Beclin-1
3966	Q99ML1	Bcl-2-binding component 3
99	Q8VDD5	Myosin-9
199	Q9WV32	Actin-related protein 2/3 complex subunit 1B
3052	Q8JZY2	COMM domain-containing protein 10
3025	Q9D198	Pre-mRNA-splicing factor SYF2
859	P47754	F-actin-capping protein subunit alpha-2
4675	Q3U5F4	YrdC domain-containing protein, mitochondrial
4536	Q69ZZ6	Transmembrane and coiled-coil domains protein 1
2001	Q7TPE5	Probable RNA polymerase II nuclear localization protein SLC7A6
2338	Q6SKR2	Methyltransferase N6AMT1

940	Q9JIX8	Apoptotic chromatin condensation inducer in the nucleus
4744	O08934	Homeobox protein unc-4 homolog
4744.1	O08934	Homeobox protein unc-4 homolog
4744.2	O09113	Homeobox protein orthopedia
4744.3	O35085	Homeobox protein ARX
4744.4	O35137	Homeobox protein aristaless-like 4
4744.5	O35602	Retinal homeobox protein Rx
4744.6	O35690	Paired mesoderm homeobox protein 2B
4744.7	O70137	Homeobox protein aristaless-like 3
4744.8	P50481	LIM/homeobox protein Lhx3
4744.9	P53776	LIM/homeobox protein Lhx4
4744.1	P63013	Paired mesoderm homeobox protein 1
4744.11	P70390	Short stature homeobox protein 2
4744.12	Q06348	Paired mesoderm homeobox protein 2
4744.13	Q62066	Paired mesoderm homeobox protein 2A
4744.14	Q8BYH0	Dorsal root ganglia homeobox protein
4744.15	Q8C8B0	ALX homeobox protein 1
261	Q9CQS2	H/ACA ribonucleoprotein complex subunit 3
114	Q3UIL6	Pleckstrin homology domain-containing family A member 7
399	Q9DCD2	Pre-mRNA-splicing factor SYF1
3418	Q64105	Sepiapterin reductase
3704	Q9JM51	Prostaglandin E synthase
3086	Q149T7	Protein phosphatase 1J
2050	Q9DB98	Leukocyte receptor cluster member 1 homolog
2478	P83870	PHD finger-like domain-containing protein 5A
3509	Q5HZK1	Centrosomal protein of 44 kDa
5121	Q61193	Ral guanine nucleotide dissociation stimulator-like 2
3629	Q923A2	Protein Spindly
3277	Q64707	U2 small nuclear ribonucleoprotein auxiliary factor 35 kDa subur
3856	Q9CZX2	Centrosomal protein of 89 kDa
433	Q8BL97	Serine/arginine-rich splicing factor 7
4811	Q9CQT7	Desumoylating isopeptidase 1
3876	Q6NZB1	Protein arginine N-methyltransferase 6
1027	O55128	Histone deacetylase complex subunit SAP18
1513	O70279	Splicing factor ESS-2 homolog
2640	Q8CI43	Myosin light chain 6B
276	O70133	ATP-dependent RNA helicase A
4215	Q9QWY8	Arf-GAP with SH3 domain, ANK repeat and PH domain-containir
5851	Q8K385	Ferric-chelate reductase 1
3634	Q3UDW8	Heparan-alpha-glucosaminide N-acetyltransferase

5287	A2ALK8	Tyrosine-protein phosphatase non-receptor type 3
1333	Q6PAC3	DDB1- and CUL4-associated factor 13
2083	Q8CII8	Nesprin-4
5790	P13516	Acyl-CoA desaturase 1
662	Q8R5K4	Nucleolar protein 6
5265	O08638	Myosin-11
3678	Q8BGP6	Solute carrier family 25 member 40
4820	P52332	Tyrosine-protein kinase JAK1
3315	Q61062	Segment polarity protein dishevelled homolog DVL-3
5040	Q91WG5	5'-AMP-activated protein kinase subunit gamma-2
4851	Q3TVW5	Trichoplein keratin filament-binding protein
1590	P17095	High mobility group protein HMG-I/HMG-Y
3607	Q3TUH1	Phosphatidate cytidylyltransferase, mitochondrial
2523	Q921L5	Conserved oligomeric Golgi complex subunit 2
1642	Q99J09	Methylosome protein 50
53	P60710	Actin, cytoplasmic 1
53.1	P60710	Actin, cytoplasmic 1
53.2	P63260	Actin, cytoplasmic 2
4999	E9Q4Z2	Acetyl-CoA carboxylase 2
3324	Q6WKZ8	E3 ubiquitin-protein ligase UBR2
3732	Q8R5A0	N-lysine methyltransferase SMYD2
4782	Q8VBV3	Exosome complex component RRP4
4923	Q922B6	E3 ubiquitin-protein ligase TRAF7
3263	Q9Z176	Serine/threonine-protein phosphatase 2A regulatory subunit B'' s
969	Q8R3F9	Speckle targeted PIP5K1A-regulated poly(A) polymerase
4266	Q8VCX5	Calcium uptake protein 1, mitochondrial
4064	Q8C2K1	Differentially expressed in FDCP 6
60	Q9Z315	U4/U6.U5 tri-snRNP-associated protein 1
2170	Q9WV70	Nucleolar complex protein 2 homolog
3674	Q8VDL4	ADP-dependent glucokinase
4430	O35231	Kinesin-like protein KIFC3
1360	Q499E4	Zinc finger protein DZIP1L
2847	Q9WTK5	Nuclear factor NF-kappa-B p100 subunit
132	O35286	Pre-mRNA-splicing factor ATP-dependent RNA helicase DHX15
1160	Q9DCE5	p21-activated protein kinase-interacting protein 1
5193	Q91W67	Ubiquitin-like protein 7
4446	Q9DCC4	Pyrroline-5-carboxylate reductase 3
4748	Q80Z37	E3 ubiquitin-protein ligase Topors
4693	Q9D2Q3	ATPase PAAT
4564	P61460	GATOR complex protein DEPDC5

5348	Q8K4B2	Interleukin-1 receptor-associated kinase 3
3285	Q8R1X6	Spartin
56	Q8K224	RNA cytidine acetyltransferase
2458	Q8VC30	Triokinase/FMN cyclase
1785	Q9CQU5	ZW10 interactor
3398	Q80UW5	Serine/threonine-protein kinase MRCK gamma
2760	Q9R1K9	Centrin-2
964	Q6URW6	Myosin-14
2650	Q9CRG1	Transmembrane 7 superfamily member 3
4753	Q91ZR1	Ras-related protein Rab-4B
3901	Q8BHA0	INO80 complex subunit C
3855	Q69Z66	Histone H2A deubiquitinase MYSM1
2512	Q8VED8	Mitochondrial fission regulator 2
2321	Q99L90	Microspherule protein 1
1375	Q9JKX4	Protein AATF
4124	Q8R3D1	TBC1 domain family member 13
5746	Q99MU3	Double-stranded RNA-specific adenosine deaminase
4909	Q3UN02	Lysocardiolipin acyltransferase 1
2398	Q9ET26	E3 ubiquitin-protein ligase RNF114
2561	Q9R078	5'-AMP-activated protein kinase subunit beta-1
5738	Q99LM9	Transcriptional adapter 1
4741	Q8VID5	ATP-dependent DNA helicase Q5
2816	Q9CZG9	PDZ domain-containing protein 11
5041	Q9ERV1	Probable E3 ubiquitin-protein ligase makorin-2
1220	Q921N6	Probable ATP-dependent RNA helicase DDX27
3142	Q3US41	Epithelial splicing regulatory protein 1
4682	Q9CR14	E3 ubiquitin-protein ligase FANCL
3211	Q9CS74	Protein ecdysoneless homolog
5233	Q9QZN0	F-box only protein 15
2891	P27048	Small nuclear ribonucleoprotein-associated protein B
3999	Q80TI1	Pleckstrin homology domain-containing family H member 1
2435	Q9QXT5	Epidermal growth factor-like protein 7
2004	Q80UP5	Ankyrin repeat domain-containing protein 13A
500	Q9DBS8	Centrosomal protein POC5
1510	Q8BFX3	BTB/POZ domain-containing protein KCTD3
3451	Q8CGU1	Calcium-binding and coiled-coil domain-containing protein 1
3072	Q61532	Mitogen-activated protein kinase 6
2207	P62317	Small nuclear ribonucleoprotein Sm D2
2023	Q8K2I2	Coiled-coil alpha-helical rod protein 1
208	Q80W68	Kin of IRRE-like protein 1

2601	Q9D5R3	Centrosomal protein of 83 kDa
2469	Q9D3U0	tRNA pseudouridine synthase Pus10
2861	Q8BNU0	Armadillo repeat-containing protein 6
4301	O70281	Protein-tyrosine sulfotransferase 1
2017	A2A3V1	A-kinase anchor protein 17B
3128	Q9CWV0	Mitochondrial assembly of ribosomal large subunit protein 1
5815	Q924A2	Protein capicua homolog
317	O35492	Dual specificity protein kinase CLK3
5017	O55126	Protein NipSnap homolog 2
2660	Q64702	Serine/threonine-protein kinase PLK4
3873	Q920E5	Farnesyl pyrophosphate synthase
2142	O88665	Bromodomain-containing protein 7
221	Q9CQQ4	Gem-associated protein 2
3155	B2RR83	3'-5' RNA helicase YTHDC2
1530	O35130	Ribosomal RNA small subunit methyltransferase NEP1
5871	Q01705	Neurogenic locus notch homolog protein 1
4509	Q8R2R1	Protein O-mannosyl-transferase 1
3306	Q8BZ20	Protein mono-ADP-ribosyltransferase PARP12
1858	Q9CQA5	Mediator of RNA polymerase II transcription subunit 4
391	Q7TSE6	Serine/threonine-protein kinase 38-like
5773	Q8C3W1	Uncharacterized protein C1orf198 homolog
5883	O35083	1-acyl-sn-glycerol-3-phosphate acyltransferase alpha
5724	Q80V94	AP-4 complex subunit epsilon-1
4498	Q8K1J6	CCA tRNA nucleotidyltransferase 1, mitochondrial
3361	P52927	High mobility group protein HMGI-C
3808	P09925	Surfeit locus protein 1
3067	E9Q6J5	Biorientation of chromosomes in cell division protein 1-like 1
2853	Q9D4H9	PHD finger protein 14
1080	Q0VGT2	Zinc finger protein GLI2
3012	P62965	Cellular retinoic acid-binding protein 1
4832	Q91VU0	Protein FAM3C
5245	Q9Z2F6	B-cell lymphoma 3 protein homolog
3077	Q9CWR2	Histone-lysine N-methyltransferase SMYD3
1970	Q99L13	3-hydroxyisobutyrate dehydrogenase, mitochondrial
5424	Q8R1F9	Ribonuclease P protein subunit p40
4736	Q6NZK5	Protein hinderin
1303	Q9CXJ1	Probable glutamate--tRNA ligase, mitochondrial
4737	Q3UZ01	RNA-binding region-containing protein 3
3405	B1AUR6	Protein MMS22-like
229	P53026	60S ribosomal protein L10a

4557	Q8CFV9	Riboflavin kinase
50	P53569	CCAAT/enhancer-binding protein zeta
2319	Q9D8M7	PHD finger protein 10
3526	Q9CY62	E3 ubiquitin-protein ligase RNF181
5893	Q9CPY3	Sororin
214	Q68FH0	Plakophilin-4
4259	Q3U319	E3 ubiquitin-protein ligase BRE1B
4799	Q9D2X5	MAU2 chromatid cohesion factor homolog
4477	Q921X6	DNA-directed RNA polymerase III subunit RPC6
5563	Q8VEF1	Protein Aster-A
4833	Q8K3K7	1-acyl-sn-glycerol-3-phosphate acyltransferase beta
4010	Q9JK81	UPF0160 protein MYG1, mitochondrial
5443	Q7TPV2	E3 ubiquitin-protein ligase DZIP3
5282	Q9D7K2	CST complex subunit TEN1
5099	Q6ZQA0	Neurobeachin-like protein 2
3617	Q9Z2R6	Protein unc-119 homolog A
2880	Q80YV2	Nuclear-interacting partner of ALK
3560	Q9JMA2	Queuine tRNA-ribosyltransferase catalytic subunit 1
4843	Q5SV85	Synergin gamma
1746	Q925E7	Serine/threonine-protein phosphatase 2A 55 kDa regulatory sub
4051	Q9JJZ4	Ubiquitin-conjugating enzyme E2 J1
4238	Q9WTK2	Chromodomain Y-like protein
3421	Q9DCA2	28S ribosomal protein S11, mitochondrial
2568	O08740	DNA-directed RNA polymerase II subunit RPB11
5445	Q8VDZ4	Palmitoyltransferase ZDHHC5
4427	Q8BHI7	Elongation of very long chain fatty acids protein 5
5458	Q9D279	Mitotic interactor and substrate of PLK1
1352	O70481	E3 ubiquitin-protein ligase UBR1
1167	Q8R050	Eukaryotic peptide chain release factor GTP-binding subunit ER
3296	Q9JHI7	Exosome complex component RRP45
3817	Q8BUY8	G-protein coupled receptor-associated sorting protein 2
2475	P21981	Protein-glutamine gamma-glutamyltransferase 2
5957	O88329	Unconventional myosin-Ia
4535	O88351	Inhibitor of nuclear factor kappa-B kinase subunit beta
3598	P51175	Protoporphyrinogen oxidase
3599	Q9ES34	Ubiquitin-protein ligase E3B
3391	Q66JQ7	Kinetochore scaffold 1
2894	P97819	85/88 kDa calcium-independent phospholipase A2
3458	Q8R4Z4	ETS translocation variant 3
5963	Q9D6N1	Carbonic anhydrase 13

2714	Q640P7	TBCC domain-containing protein 1
4587	Q6Y685	Transforming acidic coiled-coil-containing protein 1
3027	Q91YD3	mRNA-decapping enzyme 1A
5807	Q8CIB6	Transmembrane protein 230
504	Q7TND5	Ribosome production factor 1
2211	Q9WVD5	Mitochondrial ornithine transporter 1
3655	Q9WV66	E3 ubiquitin-protein ligase MARCHF7
745	Q99NH2	Partitioning defective 3 homolog
2348	Q148V8	Protein FAM83H
1674	Q99KG5	Lipolysis-stimulated lipoprotein receptor
4902	Q8BW96	Calcium/calmodulin-dependent protein kinase type 1D
3308	Q6A037	NEDD4-binding protein 1
3755	Q8BTI9	Phosphatidylinositol 4,5-bisphosphate 3-kinase catalytic subuni
5163	Q921H9	Cytochrome c oxidase assembly factor 7
2180	Q8BMK0	Centrosomal protein of 85 kDa
1591	Q9WTX2	Interferon-inducible double-stranded RNA-dependent protein kir
5014	Q8CFL8	Zinc finger SWIM domain-containing protein 3
5056	Q9DB34	Charged multivesicular body protein 2a
1715	P62305	Small nuclear ribonucleoprotein E
227	Q91YU8	Suppressor of SWI4 1 homolog
2983	Q5HZJ0	Ribonuclease 3
3272	Q8CD26	Solute carrier family 35 member E1
3951	Q91WF7	Polyphosphoinositide phosphatase
5155	Q8R5A6	TBC1 domain family member 22A
897	Q8BH44	Coronin-2B
4642	Q69Z89	Ras-associating and dilute domain-containing protein
828	Q6Y7W8	GRB10-interacting GYF protein 2
4234	Q9EPJ9	ADP-ribosylation factor GTPase-activating protein 1
2174	Q80X85	28S ribosomal protein S7, mitochondrial
4650	P56581	G/T mismatch-specific thymine DNA glycosylase
4524	Q0VF22	Coiled-coil domain-containing protein 138
3972	Q9R117	Non-receptor tyrosine-protein kinase TYK2
1546	Q9QZ23	NFU1 iron-sulfur cluster scaffold homolog, mitochondrial
5514	Q3U0M1	Trafficking protein particle complex subunit 9
743	Q920L1	Acyl-CoA (8-3)-desaturase
205	P23249	Putative helicase MOV-10
3612	Q922R0	cAMP-dependent protein kinase catalytic subunit PRKX
3373	P35285	Ras-related protein Rab-22A
3521	Q8K2H1	Periphilin-1
4974	Q99J83	Autophagy protein 5

1788	Q9D8C3	Putative bifunctional UDP-N-acetylglucosamine transferase and
4229	P70699	Lysosomal alpha-glucosidase
2973	Q91YE7	RNA-binding protein 5
5737	Q8BKE9	Intraflagellar transport protein 74 homolog
905	Q91VN4	MICOS complex subunit Mic25
2232	Q80UF4	Serologically defined colon cancer antigen 8 homolog
2502	Q8C0L6	Peroxisomal N(1)-acetyl-spermine/spermidine oxidase
1581	Q9DC33	High mobility group protein 20A
1919	Q8R3N6	THO complex subunit 1
1254	Q8BT07	Centrosomal protein of 55 kDa
564	Q64324	Syntaxin-binding protein 2
3877	Q8VBW6	NEDD8-activating enzyme E1 regulatory subunit
3021	Q61474	RNA-binding protein Musashi homolog 1
5465	Q2TPA8	Hydroxysteroid dehydrogenase-like protein 2
5485	Q9D125	28S ribosomal protein S25, mitochondrial
3350	Q8C3S2	Transport and Golgi organization protein 6 homolog
5551	Q9JKL5	Calcineurin B homologous protein 3
2446	Q5U4C3	Splicing factor, arginine/serine-rich 19
4102	Q7TQJ8	Wilms tumor protein 1-interacting protein
4852	Q8BZZ3	NEDD4-like E3 ubiquitin-protein ligase WWP1
5896	P12813	Nuclear receptor subfamily 4 group A member 1
5120	Q6ZQ03	Formin-binding protein 4
5232	Q921U8	Smoothelin
3089	Q59J78	NADH dehydrogenase [ubiquinone] 1 alpha subcomplex assembl
3252	Q8K0H5	Transcription initiation factor TFIID subunit 10
490	O88291	DBIRD complex subunit ZNF326
4706	Q8VBX6	Multiple PDZ domain protein
4156	D3YZP9	Coiled-coil domain-containing protein 6
3485	Q9D1H8	39S ribosomal protein L53, mitochondrial
5102	Q9EQG9	Ceramide transfer protein
1966	Q9JM98	WD repeat-containing protein WRAP73
2718	Q8BX57	PX domain-containing protein kinase-like protein
3750	Q9CRC0	Vitamin K epoxide reductase complex subunit 1
3705	Q9CYZ6	Required for excision 1-B domain-containing protein
1307	P08122	Collagen alpha-2(IV) chain
1614	P62320	Small nuclear ribonucleoprotein Sm D3
696	Q8BTT6	Digestive organ expansion factor homolog
3702	Q99LB0	Deoxynucleotidyltransferase terminal-interacting protein 1
4946	Q9D2R8	28S ribosomal protein S33, mitochondrial
4532	Q8CHS8	Vacuolar protein sorting-associated protein 37A

3238	B2RUR8	OTU domain-containing protein 7B
2268	Q8C5W0	Calmin
3132	Q5Y5T2	Palmitoyltransferase ZDHHC18
2045	P21271	Unconventional myosin-Vb
3000	Q8BIF2	RNA binding protein fox-1 homolog 3
3000.1	Q8BIF2	RNA binding protein fox-1 homolog 3
3000.2	Q8BP71	RNA binding protein fox-1 homolog 2
3000.3	Q9JJ43	RNA binding protein fox-1 homolog 1
1000	Q5SUA5	Unconventional myosin-Ig
3289	P35822	Receptor-type tyrosine-protein phosphatase kappa
3748	Q9DBV3	Probable ATP-dependent RNA helicase DHX34
1792	Q8BGD9	Eukaryotic translation initiation factor 4B
3917	Q14AI0	Sister chromatid cohesion protein DCC1
4526	P19246	Neurofilament heavy polypeptide
4952	Q8K248	4-hydroxyphenylpyruvate dioxygenase-like protein
4677	Q9Z2Q5	39S ribosomal protein L40, mitochondrial
4376	Q3TV70	Nuclear receptor 2C2-associated protein
5662	Q9DCL2	Cytosolic iron-sulfur assembly component 2A
5237	Q6PIU9	Uncharacterized protein FLJ45252 homolog
5223	G5E8Z2	Transcription initiation factor TFIID subunit 4B
4312	Q9ES74	Serine/threonine-protein kinase Nek7
773	Q02248	Catenin beta-1
3864	O54781	SRSF protein kinase 2
5583	Q9WV95	Pleckstrin homology-like domain family A member 3
3552	Q9Z2X8	Kelch-like ECH-associated protein 1
4166	A2A7Y5	Migration and invasion-inhibitory protein
3718	Q9QXV8	Protein sprouty homolog 2
3875	Q9CXY9	GPI-anchor transamidase
1227	Q64331	Unconventional myosin-VI
5930	Q8C4X1	Centromere protein X
2889	Q6PD29	Zinc finger protein 513
4756	Q8C080	Sorting nexin-16
4353	Q91W18	Tudor domain-containing protein 3
5164	Q9EQR6	Fanconi anemia group G protein homolog
5055	Q6PD19	Armadillo-like helical domain-containing protein 3
3178	Q8BIA4	F-box/WD repeat-containing protein 8
3402	Q8C5P7	Testis development-related protein
1980	Q62418	Drebrin-like protein
326	Q8K4Z5	Splicing factor 3A subunit 1
3468	P58390	Small conductance calcium-activated potassium channel protei

4977	Q8C079	Striatin-interacting protein 1
2410	Q9ESJ7	Interleukin-1 receptor-associated kinase 1-binding protein 1
4374	Q9D0F4	NF-kappa-B-activating protein
1743	Q8CGZ0	Calcium homeostasis endoplasmic reticulum protein
2751	Q8K2H2	Deubiquitinase OTUD6B
4426	Q99KK9	Histidine--tRNA ligase, mitochondrial
5401	Q6ZQ89	E3 ubiquitin-protein ligase MARCHF6
5692	Q8BKT8	HAUS augmin-like complex subunit 7
1278	Q6P5D4	Centrosomal protein of 135 kDa
2107	E9QAT4	Protein transport protein Sec16A
5229	Q8R079	Bifunctional apoptosis regulator
3628	Q62073	Mitogen-activated protein kinase kinase kinase 7
2814	E9PYL2	Proline-rich protein 12
5185	Q80WQ6	Inactive rhomboid protein 2
1902	Q8K2V1	Serine/threonine-protein phosphatase 4 regulatory subunit 1
3379	E9Q9D5	Rab-like protein 2A
3030	P58771	Tropomyosin alpha-1 chain
3688	Q3TIU4	2',5'-phosphodiesterase 12
5291	P51944	Cyclin-F
289	L0N7N1	Kinesin-like protein KIF14
5961	Q7TMW6	Cytosolic iron-sulfur assembly component 3
5975	Q6P0X2	Zinc finger protein 511
3280	Q5SXY1	Cytospin-B
1491	Q91VL9	Zinc finger and BTB domain-containing protein 1
2449	O35435	Dihydroorotate dehydrogenase (quinone), mitochondrial
2339	Q9QZD8	Mitochondrial dicarboxylate carrier
3530	Q8VIJ8	GATOR complex protein NPRL3
5598	B1AUE5	Peroxisome biogenesis factor 10
2702	Q3URD3	Sarcolemmal membrane-associated protein
5489	Q8C2P3	tRNA-dihydrouridine(16/17) synthase [NAD(P)(+)]-like
4325	Q9QXE4	Tumor protein p53-inducible nuclear protein 1
4415	Q9DCI3	STARD3 N-terminal-like protein
4975	Q8VC51	Telomerase Cajal body protein 1
3669	Q3TC46	Protein PAT1 homolog 1
4519	Q69Z69	N-acetyltransferase ESCO1
4131	Q8R0S2	IQ motif and SEC7 domain-containing protein 1
5298	P40694	DNA-binding protein SMUBP-2
2357	Q7JJ13	Bromodomain-containing protein 2
5623	Q8VDH1	F-box only protein 21
4939	Q9D1L9	Ragulator complex protein LAMTOR5

5832	Q9DAM5	Mitochondrial thiamine pyrophosphate carrier
3423	Q9CQC9	GTP-binding protein SAR1b
4780	Q9JHP7	Protein O-glucosyltransferase 2
3512	P70196	TNF receptor-associated factor 6
2393	Q91WE6	Threonylcarbamoyladenosine tRNA methylthiotransferase
5415	Q640L3	Cell cycle progression protein 1
1075	P58022	Lysyl oxidase homolog 2
2719	A2A6A1	G patch domain-containing protein 8
1189	Q6P9Q6	FK506-binding protein 15
4199	Q9CRA5	Golgi phosphoprotein 3
4887	Q9JJA9	General receptor for phosphoinositides 1-associated scaffold pr
4332	P23492	Purine nucleoside phosphorylase
3643	Q62419	Endophilin-A2
4256	Q8K2C8	Glycerol-3-phosphate acyltransferase 4
2242	Q80TH2	Erbin
3727	O70157	DNA topoisomerase 3-alpha
2094	Q3U308	Cytoplasmic tRNA 2-thiolation protein 2
734	Q60952	Centrosome-associated protein CEP250
5406	Q9WTZ1	RING-box protein 2
5488	Q9QZR0	E3 ubiquitin-protein ligase RNF25
5096	Q8CB44	GRAM domain-containing protein 4
3374	Q8K1R7	Serine/threonine-protein kinase Nek9
179	Q8K310	Matrin-3
3482	Q8BVD5	MAGUK p55 subfamily member 7
5966	Q8CHG3	GRIP and coiled-coil domain-containing protein 2
4363	Q80YS6	Actin filament-associated protein 1
4856	Q9R1V7	Disintegrin and metalloproteinase domain-containing protein 23
354	Q7M6U3	Inactive serine/threonine-protein kinase TEX14
2932	Q9Z1X2	Phosphatidylserine synthase 2
1049	Q64012	RNA-binding protein Raly
4647	Q921E2	Ras-related protein Rab-31
4609	Q8C176	Transcription initiation factor TFIID subunit 2
379	Q00PI9	Heterogeneous nuclear ribonucleoprotein U-like protein 2
3234	A2AJ15	Endoplasmic reticulum mannosyl-oligosaccharide 1,2-alpha-mar
3103	P46737	Lys-63-specific deubiquitinase BRCC36
5951	Q8BL48	RING finger protein unkempt homolog
4482	Q9EP53	Hamartin
5413	Q8K2X8	General transcription factor IIH subunit 5
1182	Q8R3C0	Mini-chromosome maintenance complex-binding protein
2391	O70579	Peroxisomal membrane protein PMP34

5719	P48967	M-phase inducer phosphatase 3
5931	P52623	Uridine-cytidine kinase 1
1575	O88879	Apoptotic protease-activating factor 1
5932	P97873	Lysyl oxidase homolog 1
580	P97287	Induced myeloid leukemia cell differentiation protein Mcl-1 hom[illegible]
1176	A2A4P0	ATP-dependent RNA helicase DHX8
311	Q05CL8	La-related protein 7
62	Q587J6	LINE-1 type transposase domain-containing protein 1
441	P10493	Nidogen-1
3956	Q3UHH1	Zinc finger SWIM domain-containing protein 8
837	Q6NV83	U2 snRNP-associated SURP motif-containing protein
5013	B1AX39	Zinc finger CCHC domain-containing protein 7
5125	Q9QZ11	Exonuclease 1
3408	P84104	Serine/arginine-rich splicing factor 3
3323	Q9CWF6	Bardet-Biedl syndrome 2 protein homolog
4061	Q6IRU2	Tropomyosin alpha-4 chain
2236	P62960	Y-box-binding protein 1
5410	Q9WUU8	TNFAIP3-interacting protein 1
4740	P23950	mRNA decay activator protein ZFP36L1
4168	Q8BGY4	Kelch-like protein 26
5788	Q45VK7	Cytoplasmic dynein 2 heavy chain 1
5568	Q5ND52	rRNA methyltransferase 3, mitochondrial
5529	Q8BJM5	Zinc transporter 6
3311	P28867	Protein kinase C delta type
3582	Q4VC33	E3 ubiquitin-protein transferase MAEA
570	O09005	Sphingolipid delta(4)-desaturase DES1
1085	Q8C3Y4	Kinetochore-associated protein 1
38	P39447	Tight junction protein ZO-1
3465	O35711	Liprin-beta-2
2250	P09803	Cadherin-1
3247	Q6NZN1	Peroxisome proliferator-activated receptor gamma coactivator-[illegible]
4358	Q6P5G3	MBT domain-containing protein 1
5750	Q08274	Dystrophia myotonica WD repeat-containing protein
1205	P35123	Ubiquitin carboxyl-terminal hydrolase 4
3268	Q922B9	Protein ITPRID2
5210	Q9D975	Sulfiredoxin-1
2425	Q62311	Transcription initiation factor TFIID subunit 6
2953	Q80VY9	ATP-dependent RNA helicase DHX33
3011	Q66GT5	Phosphatidylglycerophosphatase and protein-tyrosine phosphat[illegible]
4965	Q6TXD4	Dynamin-binding protein

45	E9Q309	Centrosome-associated protein 350
5604	Q6NXY9	DNA-directed RNA polymerase III subunit RPC7
4305	Q61578	NADPH:adrenodoxin oxidoreductase, mitochondrial
5513	P97823	Acyl-protein thioesterase 1
4996	Q69ZL1	FYVE, RhoGEF and PH domain-containing protein 6
5264	Q920Q6	RNA-binding protein Musashi homolog 2
4079	Q8R313	Exocyst complex component 6
83	P11276	Fibronectin
1483	Q9DBB4	N-alpha-acetyltransferase 16, NatA auxiliary subunit
3619	Q9JM13	Rab5 GDP/GTP exchange factor
527	Q8CEE0	Centrosomal protein of 57 kDa
1997	Q8BU31	Ras-related protein Rap-2c
3911	Q3UFF7	Lysophospholipase-like protein 1
3293	Q920R0	Alsin
5212	Q91WU5	Arsenite methyltransferase
249	Q61001	Laminin subunit alpha-5
5581	Q8VE99	Coiled-coil domain-containing protein 115
3504	Q9QZ05	eIF-2-alpha kinase GCN2
2413	Q99L00	HAUS augmin-like complex subunit 8
3419	Q7TN75	Retrotransposon-derived protein PEG10
3581	Q3UIW5	RING finger protein 10
1271	Q9WUR9	Adenylate kinase 4, mitochondrial
1124	P68033	Actin, alpha cardiac muscle 1
1124.1	P68033	Actin, alpha cardiac muscle 1
1124.2	P68134	Actin, alpha skeletal muscle
3064	Q8CCK0	Core histone macro-H2A.2
4862	Q0VGT4	Protein ZGRF1
1072	O88441	Metaxin-2
2117	Q2TBE6	Phosphatidylinositol 4-kinase type 2-alpha
5689	Q8K004	Spermatogenesis-associated protein 2
1666	Q80Z25	Oral-facial-digital syndrome 1 protein homolog
4424	Q8K2Y0	ORC ubiquitin ligase 1
5227	Q9CXL3	Uncharacterized protein C7orf50 homolog
4966	Q91V01	Lysophospholipid acyltransferase 5
891	Q6P9L6	Kinesin-like protein KIF15
2177	Q99ML2	Non-receptor tyrosine-protein kinase TNK1
5152	Q5PRE5	Proline and serine-rich protein 1
237	E9Q5G3	Kinesin-like protein KIF23
5116	Q9D5D8	Chromodomain Y-like protein 2
2262	Q8BIL5	Protein Hook homolog 1

5359	P40630	Transcription factor A, mitochondrial
3259	O35648	Centrin-3
4337	Q8VDP2	UPF0428 protein CXorf56 homolog
3005	Q9R0L7	A-kinase anchor protein 8-like
4804	Q9JI10	Serine/threonine-protein kinase 3
2778	Q922W5	Pyrroline-5-carboxylate reductase 1, mitochondrial
3362	Q99MV7	RING finger protein 17
3257	Q8R5C5	Beta-centractin
3131	Q8BTW8	Mitochondrial tRNA methylthiotransferase CDK5RAP1
1474	Q9D0M3	Cytochrome c1, heme protein, mitochondrial
482	Q5U4C1	G-protein coupled receptor-associated sorting protein 1
3645	Q9Z1K5	E3 ubiquitin-protein ligase ARIH1
5482	Q9R0Q4	Mortality factor 4-like protein 2
3931	Q8CGC4	Protein LSM14 homolog B
5558	Q8BHZ4	Zinc finger protein 592
3342	Q7TME2	Sperm-associated antigen 5
1670	Q922D4	Serine/threonine-protein phosphatase 6 regulatory subunit 3
3307	Q3U6U5	Putative GTP-binding protein 6
5221	Q80XC3	USP6 N-terminal-like protein
5106	Q80TR8	DDB1- and CUL4-associated factor 1
5450	Q14AT2	Testis-expressed protein 11
4378	Q8K015	Centromere protein O
4510	P70295	Ancient ubiquitous protein 1
4303	Q8R1N4	NudC domain-containing protein 3
215	Q80X44	Zinc finger and BTB domain-containing protein 24
934	Q64511	DNA topoisomerase 2-beta
2334	Q8CD92	Tetratricopeptide repeat protein 27
746	P02468	Laminin subunit gamma-1
543	A2AUM9	Centrosomal protein of 152 kDa
3722	Q9JL70	Fanconi anemia group A protein homolog
5202	Q6ZPR6	Inhibitor of Bruton tyrosine kinase
2946	Q7TMV3	FAST kinase domain-containing protein 5, mitochondrial
5283	Q8BGQ4	Protein O-mannosyl-transferase 2
1174	Q9JJL8	Serine--tRNA ligase, mitochondrial
4828	Q9D5K4	S100P-binding protein
3317	Q9D6K8	FUN14 domain-containing protein 2
5126	Q9R0X0	Mediator of RNA polymerase II transcription subunit 20
712	Q62187	Transcription termination factor 1
3516	Q9Z2G9	Oxidoreductase HTATIP2
1135	Q9D880	Mitochondrial import inner membrane translocase subunit TIM5(

3441	Q8C263	Spindle and kinetochore-associated protein 3
2705	Q8BZS9	Putative pre-mRNA-splicing factor ATP-dependent RNA helicase
4070	Q8VDT9	39S ribosomal protein L50, mitochondrial
376	Q62203	Splicing factor 3A subunit 2
3641	O54692	Centromere/kinetochore protein zw10 homolog
2825	Q923J1	Transient receptor potential cation channel subfamily M membe
498	P97742	Carnitine O-palmitoyltransferase 1, liver isoform
1468	Q6VGS5	Protein Daple
5301	Q9ERB0	Synaptosomal-associated protein 29
4662	Q8K3G9	DCC-interacting protein 13-beta
2343	Q3UHX9	Putative methyltransferase C9orf114 homolog
4689	O88448	Kinesin light chain 2
3457	O70492	Sorting nexin-3
3741	Q8R0J7	Vacuolar protein sorting-associated protein 37B
3944	Q9R008	Mevalonate kinase
3258	P18654	Ribosomal protein S6 kinase alpha-3
3695	Q9CRD2	ER membrane protein complex subunit 2
1776	O35343	Importin subunit alpha-3
3320	Q8BTY2	Sodium bicarbonate cotransporter 3
4341	Q8K339	DNA/RNA-binding protein KIN17
2844	Q924H5	DNA repair protein RAD51 homolog 3
5861	D3Z7Q2	Small integral membrane protein 20
2092	P62858	40S ribosomal protein S28
753	Q91YE5	Bromodomain adjacent to zinc finger domain protein 2A
5435	Q9CR89	Endoplasmic reticulum-Golgi intermediate compartment protein
3923	Q9WU42	Nuclear receptor corepressor 2
4607	B1AZ99	OTU domain-containing protein 3
3490	Q8R1D1	Zinc finger protein 426
4940	Q6PDH0	Pleckstrin homology-like domain family B member 1
2405	P47708	Rabphilin-3A
3474	Q6KAQ7	ZZ-type zinc finger-containing protein 3
5610	Q6YND2	Zinc finger protein 653
3188	P58774	Tropomyosin beta chain
2564	P10287	Cadherin-3
3836	P24288	Branched-chain-amino-acid aminotransferase, cytosolic
5789	Q8R2R3	Alpha- and gamma-adaptin-binding protein p34
2414	Q9JK91	DNA mismatch repair protein Mlh1
5712	Q99LJ7	RCC1 and BTB domain-containing protein 2
4968	Q8C025	Cholinephosphotransferase 1
5241	A2AG06	Meiosis-specific coiled-coil domain-containing protein MEIOC

3141	Q3ULA2	F-box/WD repeat-containing protein 1A
3546	Q8VE19	GATOR complex protein MIOS
4015	Q7TSG2	RNA polymerase II subunit A C-terminal domain phosphatase
363	Q9WVM1	Rac GTPase-activating protein 1
4294	Q91X51	Golgi reassembly-stacking protein 1
3159	Q9D753	Exosome complex component RRP43
557	Q91XC0	LIM domain-containing protein ajuba
1406	P70324	T-box transcription factor TBX3
5503	Q3V4B5	COMM domain-containing protein 6
4182	Q8BUL5	Kelch-like protein 7
429	Q8CJ40	Rootletin
4979	Q7TPW1	Nexilin
697	Q9R0L6	Pericentriolar material 1 protein
5616	Q5XJY4	Presenilins-associated rhomboid-like protein, mitochondrial
3720	Q8K341	Alpha-tubulin N-acetyltransferase 1
2559	Q8R010	Aminoacyl tRNA synthase complex-interacting multifunctional pr
4661	Q61142	Spindlin-1
1775	Q924C1	Exportin-5
492	Q4FK66	Pre-mRNA-splicing factor 38A
1325	Q9CZH8	Coiled-coil domain-containing protein 77
544	Q8C0D9	Centrosomal protein of 68 kDa
5577	P97412	Lysosomal-trafficking regulator
5459	Q8C050	Ribosomal protein S6 kinase alpha-5
2748	Q9D898	Actin-related protein 2/3 complex subunit 5-like protein
1733	Q9D1C1	Ubiquitin-conjugating enzyme E2 C
4605	Q8BJL1	F-box only protein 30
118	Q9Z1X4	Interleukin enhancer-binding factor 3
5336	P59281	Rho GTPase-activating protein 39
3936	Q9R0E1	Multifunctional procollagen lysine hydroxylase and glycosyltrans
3336	Q8CFI5	Probable proline--tRNA ligase, mitochondrial
5030	P47740	Aldehyde dehydrogenase family 3 member A2
259	Q8CB62	Centrobin
3377	Q99MI1	ELKS/Rab6-interacting/CAST family member 1
5371	Q9CR76	Transmembrane protein 186
254	A2AG58	BCLAF1 and THRAP3 family member 3
2956	Q9D0T2	Dual specificity protein phosphatase 12
3998	P48025	Tyrosine-protein kinase SYK
3531	Q99LC9	Peroxisome assembly factor 2
4036	Q8CC88	von Willebrand factor A domain-containing protein 8
4147	Q9CPX6	Ubiquitin-like-conjugating enzyme ATG3

5715	Q9D289	Trafficking protein particle complex subunit 6B
4539	Q69ZJ7	RAB6A-GEF complex partner protein 1
2184	Q7TQG1	Pleckstrin homology domain-containing family A member 6
2700	Q8K2X3	CST complex subunit STN1
5948	G5E8P0	Gamma-tubulin complex component 6
5473	B0V2N1	Receptor-type tyrosine-protein phosphatase S
4478	Q8K1A6	Coiled-coil and C2 domain-containing protein 1A
41	P48725	Pericentrin
942	Q9QZL0	Receptor-interacting serine/threonine-protein kinase 3
807	Q8C854	Myelin expression factor 2
371	Q3UJV1	Coiled-coil domain-containing protein 61
5222	A3KGS3	Ral GTPase-activating protein subunit alpha-2
76	Q8K389	CDK5 regulatory subunit-associated protein 2
4068	P63034	Cytohesin-2
5475	Q8K0D7	Guided entry of tail-anchored proteins factor 1
2257	Q9WU79	Proline dehydrogenase 1, mitochondrial
900	Q9JIX0	Transcription and mRNA export factor ENY2
5921	Q9ESN9	C-Jun-amino-terminal kinase-interacting protein 3
159	O88322	Nidogen-2
3775	Q8BGS7	Choline/ethanolaminephosphotransferase 1
1461	Q3U1G5	Interferon-stimulated 20 kDa exonuclease-like 2
5036	Q7M757	Lys-63-specific deubiquitinase BRCC36-like
1330	Q8C5L7	RNA-binding protein 34
1723	P31230	Aminoacyl tRNA synthase complex-interacting multifunctional pr
2074	P56371	Ras-related protein Rab-4A
5292	Q80TZ9	Arginine-glutamic acid dipeptide repeats protein
5209	Q8BQ33	Treslin
4290	Q3U1J1	TCF3 fusion partner homolog
3151	Q8BYY4	Tetratricopeptide repeat protein 39B
77	B1AQJ2	Ubiquitin carboxyl-terminal hydrolase 36
1632	Q9DB96	Neuroguidin
2197	P06537	Glucocorticoid receptor
4986	Q3U0V2	Tumor necrosis factor receptor type 1-associated DEATH doma
2501	Q922Y1	UBX domain-containing protein 1
2190	Q9WTV7	E3 ubiquitin-protein ligase RLIM
2073	P21619	Lamin-B2
1262	Q923E4	NAD-dependent protein deacetylase sirtuin-1
3871	Q8BQX5	Transmembrane and coiled-coil domain-containing protein 6
3796	Q62417	Sorbin and SH3 domain-containing protein 1
2913	Q9R118	Serine protease HTRA1

3638	Q9R1P4	Proteasome subunit alpha type-1
1339	Q811P8	Rho GTPase-activating protein 32
2711	Q8VDS7	Centrosomal protein CEP57L1
273	Q9WVR4	Fragile X mental retardation syndrome-related protein 2
615	Q640L5	Coiled-coil domain-containing protein 18
3670	Q6AXC6	ATP-dependent DNA helicase DDX11
3929	Q6PGF7	Exocyst complex component 8
5382	Q9D2U5	N-alpha-acetyltransferase 38, NatC auxiliary subunit
238	Q5SYD0	Unconventional myosin-Id
3309	Q99JW4	LIM and senescent cell antigen-like-containing domain protein 1
1730	Q8VC65	Nurim
5492	Q6PGB8	Probable global transcription activator SNF2L1
2708	Q9D1E8	1-acyl-sn-glycerol-3-phosphate acyltransferase epsilon
3177	Q6ZQK5	Arf-GAP with coiled-coil, ANK repeat and PH domain-containing
1455	O35465	Peptidyl-prolyl cis-trans isomerase FKBP8
3879	Q9JKW0	ADP-ribosylation factor-like protein 6-interacting protein 1
1836	Q7TSG1	Centrosomal protein of 120 kDa
2976	Q80X32	UPF0461 protein C5orf24 homolog
3110	Q9Z179	SHC SH2 domain-binding protein 1
3906	E9Q8T2	PR domain zinc finger protein 15
4044	O70576	Cohesin subunit SA-3
5699	O88574	Histone deacetylase complex subunit SAP30
5032	Q921Q3	Chitobiosyldiphosphodolichol beta-mannosyltransferase
420	Q9CWX9	Probable ATP-dependent RNA helicase DDX47
5195	Q6P1D7	Structure-specific endonuclease subunit SLX4
309	Q3UHX0	Nucleolar protein 8
2689	O88907	E3 SUMO-protein ligase PIAS1
78	Q9ERG0	LIM domain and actin-binding protein 1
3119	Q9QXN0	Protein Shroom3
5158	Q9CQY5	Magnesium transporter protein 1
2899	Q80U38	Protein KHNYN
456	Q61292	Laminin subunit beta-2
2192	Q9CQ22	Ragulator complex protein LAMTOR1
2836	Q7TSH4	Centriolar coiled-coil protein of 110 kDa
5220	O35387	HCLS1-associated protein X-1
344	Q8CJF7	Protein ELYS
3138	Q3TCH7	Cullin-4A
5704	O35739	Krueppel-like factor 9
3195	P70188	Kinesin-associated protein 3
5819	P28322	ETS translocation variant 4

5819.1	P28322	ETS translocation variant 4
5819.2	P41164	ETS translocation variant 1
5819.3	Q9CXC9	ETS translocation variant 5
3248	Q8C170	Unconventional myosin-IXa
672	Q9D666	SUN domain-containing protein 1
1750	Q62388	Serine-protein kinase ATM
3893	Q5SSM3	Rho GTPase-activating protein 44
5532	Q9D924	Iron-sulfur cluster assembly 1 homolog, mitochondrial
2165	Q8K4J6	Myocardin-related transcription factor A
3471	Q8BKX6	Serine/threonine-protein kinase SMG1
5387	P97429	Annexin A4
372	Q9QXS1	Plectin
4500	Q5I1X5	RelA-associated inhibitor
2947	Q3UYG1	Coiled-coil domain-containing protein 160
991	Q9JLR1	Protein transport protein Sec61 subunit alpha isoform 2
3591	A2A9C3	KICSTOR complex protein SZT2
4244	P01901	H-2 class I histocompatibility antigen, K-B alpha chain
1705	Q76LS9	Ubiquitin carboxyl-terminal hydrolase MINDY-1
846	Q8CH02	SURP and G-patch domain-containing protein 1
4011	Q99NF3	Centrosomal protein of 41 kDa
447	P26231	Catenin alpha-1
2694	Q9QZS0	Collagen alpha-3(IV) chain
2335	P02463	Collagen alpha-1(IV) chain
1718	Q8BQM4	HEAT repeat-containing protein 3
1191	Q5U4D9	THO complex subunit 6 homolog
4581	Q9CPQ5	Centromere protein Q
1393	Q8BX02	KN motif and ankyrin repeat domain-containing protein 2
1813	Q9CXF4	TBC1 domain family member 15
4879	Q3UHQ6	Protein dopey-2
327	Q99KG3	RNA-binding protein 10
3167	Q9D1C8	Vacuolar protein sorting-associated protein 28 homolog
1771	Q9D3R3	Centrosomal protein of 72 kDa
312	A2AQ25	Sickle tail protein
4180	P11688	Integrin alpha-5
3693	Q8N7N5	DDB1- and CUL4-associated factor 8
2121	Q9JJ66	Cell division cycle protein 20 homolog
4298	Q7TMK6	Protein Hook homolog 2
3264	Q8K2M0	39S ribosomal protein L38, mitochondrial
3437	Q3TDD9	Protein phosphatase 1 regulatory subunit 21
2310	Q9Z1J3	Cysteine desulfurase, mitochondrial

658	Q04750	DNA topoisomerase 1
1873	Q8WTY4	Anamorsin
1628	P28271	Cytoplasmic aconitate hydratase
4032	Q8BVA5	Lipid droplet-associated hydrolase
4546	Q8CI12	Smoothelin-like protein 2
240	Q9CRB9	MICOS complex subunit Mic19
4030	Q8BTE5	Protein CEBPZOS
5362	Q8BGT7	Survival of motor neuron-related-splicing factor 30
4403	P47791	Glutathione reductase, mitochondrial
5373	Q9WVH4	Forkhead box protein O3
2656	Q9CQ56	Vesicle transport protein USE1
5670	O54967	Activated CDC42 kinase 1
3204	Q9WTQ8	Mitochondrial import inner membrane translocase subunit Tim2[illegible]
1598	Q80UV9	Transcription initiation factor TFIID subunit 1
5003	Q8R216	NAD-dependent protein lipoamidase sirtuin-4, mitochondrial
2653	Q9D706	RNA polymerase II-associated protein 3
3858	Q9Z2W1	Serine/threonine-protein kinase 25
689	Q8C0T5	Signal-induced proliferation-associated 1-like protein 1
793	Q925I1	ATPase family AAA domain-containing protein 3
5479	O54714	E3 SUMO-protein ligase PIAS3
5345	Q3TV65	MPN domain-containing protein
4825	Q9CXC3	Mitochondrial genome maintenance exonuclease 1
5240	Q921I2	Kelch domain-containing protein 4
4622	Q4KML4	Costars family protein ABRACL
2493	Q922R1	UPF0183 protein C16orf70 homolog
647	Q8VDQ9	Protein KRI1 homolog
3514	Q9WUF3	CASP8-associated protein 2
1826	Q9EQW7	Kinesin-like protein KIF13A
1545	P14869	60S acidic ribosomal protein P0
2287	Q3UPF5	Zinc finger CCCH-type antiviral protein 1
4695	Q9EQS3	c-Myc-binding protein
3229	Q8BKE6	Cytochrome P450 20A1
4668	Q62136	Tyrosine-protein phosphatase non-receptor type 21
5477	P49615	Cyclin-dependent-like kinase 5
355	Q61235	Beta-2-syntrophin
2917	O54826	Protein AF-10
5806	Q9JI90	E3 ubiquitin-protein ligase RNF14
731	Q6PIP5	NudC domain-containing protein 1
530	Q8BHD7	Polypyrimidine tract-binding protein 3
3843	Q99M04	Lipoyl synthase, mitochondrial

3770	Q7TSI1	Pleckstrin homology domain-containing family M member 1
1892	Q80U58	Pumilio homolog 2
868	P53996	Cellular nucleic acid-binding protein
4483	Q03249	Galactose-1-phosphate uridylyltransferase
3996	Q9ESK9	RB1-inducible coiled-coil protein 1
985	P55821	Stathmin-2
4101	A2RSY6	TRMT1-like protein
3580	Q99LC8	Translation initiation factor eIF-2B subunit alpha
3135	Q9EST3	Eukaryotic translation initiation factor 4E transporter
2984	Q9DD02	Protein Hikeshi
495	P29341	Polyadenylate-binding protein 1
5722	Q8VDI7	Ubiquitin-associated domain-containing protein 1
3801	Q8VD76	General transcription factor IIH subunit 3
3078	P62309	Small nuclear ribonucleoprotein G
1820	Q80Y19	Rho GTPase-activating protein 11A
2651	P39061	Collagen alpha-1(XVIII) chain
1778	Q811D2	Ankyrin repeat domain-containing protein 26
5911	Q3UE17	RNA-binding protein MEX3D
5067	Q9CWY3	N-lysine methyltransferase SETD6
956	Q8BG51	Mitochondrial Rho GTPase 1
4807	Q64669	NAD(P)H dehydrogenase [quinone] 1
3460	A2AT37	Regulator of nonsense transcripts 2
4707	Q8K2T4	Ubiquinol-cytochrome-c reductase complex assembly factor 3
4137	Q8BK72	28S ribosomal protein S27, mitochondrial
1578	Q8BJS4	SUN domain-containing protein 2
3574	Q99PJ2	Probable E3 ubiquitin-protein ligase TRIM8
1445	Q1PSW8	E3 ubiquitin-protein ligase TRIM71
3844	P11679	Keratin, type II cytoskeletal 8
2724	Q8R0H9	ADP-ribosylation factor-binding protein GGA1
5111	Q99ME2	WD repeat-containing protein 6
1087	Q9CPR8	Non-structural maintenance of chromosomes element 3 homolog
2457	Q80TE4	Signal-induced proliferation-associated 1-like protein 2
3885	B2RRD7	Peregrin
3217	Q8C0L8	Conserved oligomeric Golgi complex subunit 5
2881	Q8BP00	IQ calmodulin-binding motif-containing protein 1
3344	Q8CFD4	Sorting nexin-8
3661	A8C756	Thyroid adenoma-associated protein homolog
3176	Q03963	Interferon-induced, double-stranded RNA-activated protein kina
4475	Q8CC86	Nicotinate phosphoribosyltransferase
3639	P21107	Tropomyosin alpha-3 chain

3356	E9PXF8	Myotubularin-related protein 13
4144	Q8BGX2	Mitochondrial import inner membrane translocase subunit Tim29
1252	Q9D3W4	GPN-loop GTPase 3
2535	Q62036	Centrosomal protein of 131 kDa
4499	Q9CWV1	DNA helicase MCM8
4840	P62313	U6 snRNA-associated Sm-like protein LSm6
4630	Q8CCP0	Nuclear export mediator factor Nemf
3984	Q80TQ2	Ubiquitin carboxyl-terminal hydrolase CYLD
5493	P70268	Serine/threonine-protein kinase N1
5678	Q8R1N0	Zinc finger protein 830
102	Q05793	Basement membrane-specific heparan sulfate proteoglycan core
2485	Q9DBM1	G patch domain-containing protein 1
4898	Q9DC50	Peroxisomal carnitine O-octanoyltransferase
339	Q62074	Protein kinase C iota type
2308	Q99LI5	Zinc finger protein 281
2691	Q9DCJ5	NADH dehydrogenase [ubiquinone] 1 alpha subcomplex subunit 8
473	P20152	Vimentin
3165	Q91WN1	DnaJ homolog subfamily C member 9
5507	Q62470	Integrin alpha-3
2096	Q99MJ9	ATP-dependent RNA helicase DDX50
4002	Q3TAA7	Serine/threonine-protein kinase 11-interacting protein
1996	B2RXS4	Plexin-B2
2029	P97313	DNA-dependent protein kinase catalytic subunit
4819	Q6NXK5	RNA/RNP complex-1-interacting phosphatase
5048	O70228	Probable phospholipid-transporting ATPase IIA
5087	Q3UHI4	Protein TMED8
5461	Q3UDP0	WD repeat-containing protein 41
620	Q8BI22	Centrosomal protein of 128 kDa
4468	Q3U3T8	WD repeat-containing protein 62
4749	Q8BMK1	tRNA N(3)-methylcytidine methyltransferase METTL2
1580	Q78XF5	Oligosaccharyltransferase complex subunit OSTC
3896	P83940	Elongin-C
4584	E9Q784	Zinc finger CCCH domain-containing protein 13
3036	Q8VDD8	WASH complex subunit 1
1506	A2AHC3	Calmodulin-regulated spectrin-associated protein 1
5463	Q3TMX7	Sulfhydryl oxidase 2
5624	Q9CXJ4	Mitochondrial potassium channel ATP-binding subunit
5214	Q9D1R1	Complex I assembly factor TMEM126B, mitochondrial
230	O35326	Serine/arginine-rich splicing factor 5
3115	Q6P9J5	KN motif and ankyrin repeat domain-containing protein 4

33	Q9Z0U1	Tight junction protein ZO-2
5777	Q8C561	G-protein coupled receptor-associated protein LMBRD2
2155	Q9WU56	tRNA pseudouridine synthase A
2219	Q8K0V4	CCR4-NOT transcription complex subunit 3
1824	P58043	Sestrin-2
2247	Q9D4H4	Angiomotin-like protein 1
5655	Q3UGC7	Eukaryotic translation initiation factor 3 subunit J-A
5655.1	Q3UGC7	Eukaryotic translation initiation factor 3 subunit J-A
5655.2	Q66JS6	Eukaryotic translation initiation factor 3 subunit J-B
5875	Q3U213	Protein SERAC1
2537	Q8CHV6	Transcriptional adapter 2-alpha
4362	Q9Z160	Conserved oligomeric Golgi complex subunit 1
4648	O54931	A-kinase anchor protein 2
835	Q6EJB6	U3 small nucleolar RNA-associated protein 14 homolog B
1235	Q8CH09	SURP and G-patch domain-containing protein 2
1368	Q5SSZ5	Tensin-3
2015	Q5NCX5	Neuralized-like protein 4
1688	P30285	Cyclin-dependent kinase 4
1394	A3KGV1	Outer dense fiber protein 2
5843	Q9QY39	PDZ domain-containing protein 4
769	Q8VBZ3	Cleft lip and palate transmembrane protein 1 homolog
1812	Q8BWT1	3-ketoacyl-CoA thiolase, mitochondrial
2914	E9Q7E2	AT-rich interactive domain-containing protein 2
3572	Q9ER69	Pre-mRNA-splicing regulator WTAP
1686	Q8BH07	ADP-ribosylation factor-like protein 6-interacting protein 6
2786	Q8BHL8	Proteasome inhibitor PI31 subunit
5097	Q8BFQ8	Glutamine amidotransferase-like class 1 domain-containing pro
1362	Q9CYA6	Zinc finger CCHC domain-containing protein 8
3210	Q02395	Metal-response element-binding transcription factor 2
4954	P97473	RISC-loading complex subunit TARBP2
439	Q9DBY8	Nuclear valosin-containing protein-like
5140	Q8BP78	Protein FRA10AC1 homolog
2353	Q8BRF7	Sec1 family domain-containing protein 1
5228	Q9D495	Synaptonemal complex central element protein 1
4941	Q5SRY7	F-box/WD repeat-containing protein 11
389	Q80X50	Ubiquitin-associated protein 2-like
526	Q8BX17	Gem-associated protein 5
4711	Q9DAP7	Histone chaperone ASF1B
4972	Q99PQ2	E3 ubiquitin-protein ligase TRIM11
329	P59328	WD repeat and HMG-box DNA-binding protein 1

3663	Q9D7M1	Glucose-induced degradation protein 8 homolog
1963	O70566	Protein diaphanous homolog 2
1151	Q921F2	TAR DNA-binding protein 43
5786	Q01755	T-complex protein 11
4003	Q91WG4	Elongator complex protein 2
5725	Q9JIS8	Solute carrier family 12 member 4
4441	Q8BG79	CWF19-like protein 2
875	Q9WTY1	Programmed cell death protein 7
2712	Q3UQU0	Bromodomain-containing protein 9
798	P26039	Talin-1
3145	P51949	CDK-activating kinase assembly factor MAT1
2438	O88844	Isocitrate dehydrogenase [NADP] cytoplasmic
1448	Q7TNP2	Serine/threonine-protein phosphatase 2A 65 kDa regulatory sub
5242	O09118	Netrin-1
1157	P52825	Carnitine O-palmitoyltransferase 2, mitochondrial
4481	P00520	Tyrosine-protein kinase ABL1
3647	Q9DC23	DnaJ homolog subfamily C member 10
2116	P10853	Histone H2B type 1-F/J/L
2116.1	P10853	Histone H2B type 1-F/J/L
2116.2	P10854	Histone H2B type 1-M
2116.3	Q64475	Histone H2B type 1-B
2116.4	Q64478	Histone H2B type 1-H
2116.5	Q64525	Histone H2B type 2-B
2116.6	Q6ZWY9	Histone H2B type 1-C/E/G
2116.7	Q8CGP1	Histone H2B type 1-K
2116.8	Q8CGP2	Histone H2B type 1-P
5928	A2AWT3	Ataxin-7-like protein 3
5774	Q9D711	Pirin

Gene name	Molecular W	Identified Pe	Protein Grou	IgG	FLAG	F/IgG
-	12 kDa	4	0.99993721	3.4.E+04	4.8.E+08	1.4.E+04
Lyar	44 kDa	16	0.99999948	1.9.E+06	5.7.E+09	3.0.E+03
Smad7	46 kDa	1	0.99402668	2.5.E+01	6.6.E+04	2.6.E+03
Trappc5	21 kDa	1	0.99639743	9.5.E+02	6.5.E+05	6.8.E+02
Gjb3	31 kDa	3	0.99972095	2.6.E+03	1.4.E+06	5.3.E+02
Xpr1	82 kDa	2	0.9802233	9.5.E+02	4.8.E+05	5.1.E+02
Arel1	94 kDa	1	0.99084473	1.7.E+02	7.0.E+04	4.1.E+02
Lsm4	15 kDa	1	0.9997641	6.9.E+03	2.6.E+06	3.8.E+02
Ap5m1	54 kDa	2	0.9800363	1.4.E+03	5.1.E+05	3.7.E+02
Foxo4	54 kDa	2	0.9912904	1.8.E+03	6.3.E+05	3.5.E+02
Cdca3	29 kDa	2	0.99991022	1.4.E+03	4.3.E+05	3.0.E+02
Oxnad1	35 kDa	4	0.99943193	1.5.E+03	4.5.E+05	3.0.E+02
Bop1	83 kDa	17	0.99999951	1.3.E+06	3.7.E+08	2.9.E+02
D1Pas1	73 kDa	28	0.99457533	1.7.E+03	4.8.E+05	2.8.E+02
Ifitm3	15 kDa	1	0.99979095	2.4.E+03	6.8.E+05	2.8.E+02
Casc3	76 kDa	4	0.9999993	3.5.E+03	9.5.E+05	2.8.E+02
Znf691	32 kDa	2	0.989075	5.7.E+04	1.6.E+07	2.7.E+02
Lrrc1	59 kDa	4	0.99650865	1.5.E+03	4.0.E+05	2.7.E+02
Arhgef10	148 kDa	2	0.9765717	5.3.E+03	1.4.E+06	2.6.E+02
Srebf2	123 kDa	5	0.99928085	5.1.E+03	1.3.E+06	2.5.E+02
Rest	118 kDa	32	0.99999998	2.4.E+06	5.9.E+08	2.5.E+02
Exosc3	30 kDa	3	0.9980556	3.8.E+03	9.3.E+05	2.4.E+02
Gpatch4	47 kDa	6	0.99969279	9.2.E+03	2.2.E+06	2.4.E+02
Utp4	77 kDa	19	0.99999912	1.0.E+06	2.5.E+08	2.4.E+02
Ccdc12	19 kDa	5	0.99985621	1.2.E+05	2.8.E+07	2.4.E+02
Ptges2	43 kDa	2	0.99704459	3.9.E+03	9.3.E+05	2.4.E+02
Mphosph10	79 kDa	12	0.99999898	2.5.E+05	6.1.E+07	2.4.E+02
Rrp9	52 kDa	13	0.99999999	8.9.E+05	2.0.E+08	2.3.E+02
Cwc15	27 kDa	5	0.99992158	1.5.E+05	3.5.E+07	2.2.E+02
Serpina3k	47 kDa	3	0.9859338	6.5.E+03	1.4.E+06	2.2.E+02
Eif2ak3	125 kDa	7	0.9893445	6.3.E+03	1.3.E+06	2.1.E+02
Isy1	33 kDa	6	0.99999661	2.8.E+05	5.9.E+07	2.1.E+02
Pinx1	37 kDa	6	0.99995778	4.4.E+04	9.2.E+06	2.1.E+02
Dnttip2	84 kDa	15	0.99999996	7.3.E+05	1.5.E+08	2.1.E+02
Pmf1	23 kDa	4	0.99697153	8.2.E+03	1.6.E+06	2.0.E+02
Wdr3	106 kDa	32	0.99998627	2.1.E+06	4.0.E+08	2.0.E+02
Wdr43	75 kDa	17	0.99999983	2.2.E+06	4.3.E+08	1.9.E+02
Pwp2	103 kDa	20	1	9.4.E+05	1.8.E+08	1.9.E+02
Pum3	73 kDa	23	0.99999998	1.3.E+06	2.4.E+08	1.8.E+02

Ccser2	93 kDa	4	0.99849892	4.6.E+03	8.2.E+05	1.8.E+02
Krr1	44 kDa	11	0.99999216	8.4.E+05	1.5.E+08	1.7.E+02
Magoh	17 kDa	9	0.9999985	2.8.E+06	4.8.E+08	1.7.E+02
Magoh	17 kDa	9	0.9999985	2.8.E+06	4.8.E+08	1.7.E+02
Magohb	17 kDa	9	0.9999985	2.8.E+06	4.8.E+08	1.7.E+02
Nol11	81 kDa	18	0.99999998	1.7.E+06	2.9.E+08	1.7.E+02
Wdr46	69 kDa	18	0.99999992	1.2.E+06	2.1.E+08	1.7.E+02
Utp6	70 kDa	22	0.99999888	1.6.E+06	2.7.E+08	1.7.E+02
C1orf109	24 kDa	1	0.99921031	6.5.E+03	1.1.E+06	1.7.E+02
Dym	76 kDa	3	0.99705011	5.1.E+03	8.5.E+05	1.7.E+02
Srsf10	31 kDa	8	0.99999938	1.7.E+06	2.8.E+08	1.7.E+02
Rbm8a	20 kDa	5	0.99999716	1.5.E+06	2.5.E+08	1.6.E+02
Imp3	22 kDa	8	0.99999999	8.1.E+05	1.3.E+08	1.6.E+02
Nol10	80 kDa	23	0.99999941	6.6.E+05	1.1.E+08	1.6.E+02
Rrp1	55 kDa	7	0.99999767	3.5.E+05	5.5.E+07	1.6.E+02
Tmod3	40 kDa	13	0.99999737	3.5.E+06	5.6.E+08	1.6.E+02
Tmod2	40 kDa	6	0.9995414	2.3.E+05	3.7.E+07	1.6.E+02
Wdr12	47 kDa	9	0.99998498	1.2.E+06	1.8.E+08	1.5.E+02
Kansl2	54 kDa	1	0.99922717	1.6.E+03	2.5.E+05	1.5.E+02
Rcl1	41 kDa	10	0.99999503	7.7.E+05	1.2.E+08	1.5.E+02
Tmem161b	55 kDa	2	0.99673772	3.5.E+03	5.4.E+05	1.5.E+02
Poc1a	45 kDa	6	0.9999969	4.5.E+04	6.9.E+06	1.5.E+02
Ripk1	75 kDa	3	0.99645106	6.1.E+03	9.3.E+05	1.5.E+02
Slx4ip	46 kDa	3	0.99601264	3.4.E+03	5.2.E+05	1.5.E+02
Rrp15	31 kDa	5	0.99999863	2.1.E+05	3.1.E+07	1.5.E+02
Tbl3	88 kDa	31	0.99999996	2.7.E+06	4.0.E+08	1.5.E+02
Bud13	72 kDa	7	0.99996335	8.2.E+04	1.2.E+07	1.5.E+02
Lin28a	23 kDa	11	0.99999901	1.8.E+06	2.6.E+08	1.5.E+02
Wdr19	151 kDa	7	0.99973101	9.2.E+03	1.3.E+06	1.5.E+02
Ubl5	9 kDa	3	0.99937941	5.9.E+04	8.5.E+06	1.4.E+02
Daxx	81 kDa	1	0.99581411	5.7.E+03	8.1.E+05	1.4.E+02
Trim26	63 kDa	3	0.99753212	5.9.E+03	8.3.E+05	1.4.E+02
Tra2b	34 kDa	7	0.9999899	2.5.E+05	3.5.E+07	1.4.E+02
Tktl1	65 kDa	1	0.99893414	3.7.E+05	5.2.E+07	1.4.E+02
Imp4	34 kDa	9	0.99999871	4.7.E+05	6.4.E+07	1.4.E+02
Mettl17	52 kDa	3	0.99867939	6.9.E+03	9.3.E+05	1.4.E+02
Eif4a3	47 kDa	17	0.99999959	9.3.E+06	1.2.E+09	1.3.E+02
Usp28	119 kDa	13	0.99998872	4.1.E+04	5.3.E+06	1.3.E+02
Snu13	14 kDa	5	0.99997562	1.1.E+06	1.5.E+08	1.3.E+02
Hs1bp3	44 kDa	4	0.99984454	6.4.E+04	8.0.E+06	1.2.E+02

-	31 kDa	6	0.99962694	7.6.E+04	9.4.E+06	1.2.E+02
Stk38	54 kDa	14	0.99999946	7.1.E+05	8.8.E+07	1.2.E+02
Dedd	37 kDa	1	0.9828429	1.0.E+04	1.2.E+06	1.2.E+02
Clk2	60 kDa	10	0.99999916	1.8.E+05	2.2.E+07	1.2.E+02
Maged1	86 kDa	3	0.99963826	1.0.E+04	1.3.E+06	1.2.E+02
Tra2a	32 kDa	6	0.99905649	1.1.E+06	1.3.E+08	1.2.E+02
Sf3b5	10 kDa	3	0.99999991	6.1.E+04	7.4.E+06	1.2.E+02
Atp11a	136 kDa	4	0.99474182	2.2.E+03	2.7.E+05	1.2.E+02
Nip7	20 kDa	8	0.99999799	1.0.E+06	1.2.E+08	1.2.E+02
Mrpl37	48 kDa	3	0.99998477	9.5.E+03	1.1.E+06	1.2.E+02
Commd8	21 kDa	2	0.99997535	3.5.E+03	4.2.E+05	1.2.E+02
Rsl1d1	50 kDa	15	0.99999479	5.2.E+06	6.1.E+08	1.2.E+02
Bcas2	26 kDa	10	0.99999016	3.3.E+05	3.9.E+07	1.2.E+02
Nfx1	124 kDa	6	0.99993068	7.5.E+03	8.6.E+05	1.2.E+02
Snrpa1	28 kDa	10	0.99996144	1.7.E+06	1.9.E+08	1.1.E+02
Focad	199 kDa	6	0.99795143	6.8.E+03	7.8.E+05	1.1.E+02
Clk4	57 kDa	11	0.99998953	1.7.E+04	1.9.E+06	1.1.E+02
Mboat2	59 kDa	2	0.9841609	7.8.E+03	8.7.E+05	1.1.E+02
Czib	18 kDa	3	0.99072742	8.1.E+03	8.9.E+05	1.1.E+02
Wdr74	43 kDa	15	0.9999969	4.8.E+05	5.3.E+07	1.1.E+02
Cox10	49 kDa	3	0.998667	1.3.E+04	1.4.E+06	1.1.E+02
Srsf1	28 kDa	10	0.99995978	5.1.E+06	5.5.E+08	1.1.E+02
Nmnat1	32 kDa	2	0.99411607	5.2.E+03	5.6.E+05	1.1.E+02
Saal1	53 kDa	2	0.99825719	6.0.E+03	6.4.E+05	1.1.E+02
Shkbp1	76 kDa	18	0.99991683	1.2.E+05	1.3.E+07	1.1.E+02
Cinp	24 kDa	3	0.99802503	4.6.E+03	4.9.E+05	1.1.E+02
Zcchc3	44 kDa	5	0.99983139	2.9.E+04	3.1.E+06	1.1.E+02
Fbxo5	48 kDa	2	0.99999117	4.7.E+03	5.0.E+05	1.1.E+02
Rrp7a	32 kDa	11	0.99999922	4.0.E+05	4.2.E+07	1.0.E+02
Cenpk	32 kDa	2	0.99993927	5.3.E+03	5.5.E+05	1.0.E+02
Cdc40	65 kDa	23	0.99999698	1.5.E+06	1.5.E+08	1.0.E+02
Utp18	61 kDa	19	0.99999684	2.2.E+06	2.3.E+08	1.0.E+02
Spata6	56 kDa	2	0.99339012	6.1.E+03	6.3.E+05	1.0.E+02
Cwc22	105 kDa	22	0.99998607	1.0.E+06	1.0.E+08	1.0.E+02
Myo1c	122 kDa	40	0.9999991	1.4.E+07	1.4.E+09	1.0.E+02
Srrm2	295 kDa	55	0.99999999	2.1.E+07	2.1.E+09	1.0.E+02
Ppp1r9b	90 kDa	7	0.9985655	4.1.E+04	4.1.E+06	9.9.E+01
Myo5a	216 kDa	82	0.99999999	1.0.E+07	1.0.E+09	9.8.E+01
Card19	21 kDa	2	0.99605745	1.4.E+04	1.4.E+06	9.7.E+01
Utp15	59 kDa	15	0.99999716	2.2.E+06	2.1.E+08	9.6.E+01

Ppie	33 kDa	9	0.99990202	8.2.E+05	7.8.E+07	9.5.E+01
Nop2	87 kDa	17	0.99999838	3.2.E+06	3.1.E+08	9.4.E+01
Wfs1	101 kDa	2	0.99841727	4.8.E+03	4.4.E+05	9.3.E+01
Mpp5	77 kDa	4	0.99741864	1.2.E+04	1.1.E+06	9.2.E+01
Wdr75	94 kDa	30	0.99999994	4.2.E+06	3.9.E+08	9.2.E+01
Smu1	58 kDa	17	0.99999908	1.6.E+06	1.4.E+08	9.2.E+01
Tsen15	19 kDa	1	0.99688414	4.8.E+03	4.4.E+05	9.2.E+01
Bud31	17 kDa	8	0.99996584	6.4.E+05	5.8.E+07	9.1.E+01
Rpf2	35 kDa	14	1	2.7.E+06	2.4.E+08	9.0.E+01
Tmem120a	41 kDa	4	0.99970493	2.8.E+04	2.5.E+06	9.0.E+01
Cwc27	54 kDa	5	0.99998163	2.9.E+04	2.6.E+06	8.9.E+01
Utp14a	87 kDa	24	0.99999995	1.8.E+06	1.5.E+08	8.8.E+01
Bola1	14 kDa	4	0.99999958	1.7.E+04	1.5.E+06	8.7.E+01
Cnn2	33 kDa	3	0.99933661	4.0.E+03	3.4.E+05	8.5.E+01
Sparc	34 kDa	3	0.99788258	3.0.E+04	2.6.E+06	8.5.E+01
Snrpf	10 kDa	2	0.9970042	4.6.E+04	3.9.E+06	8.5.E+01
Pes1	68 kDa	17	0.999984	3.0.E+06	2.5.E+08	8.4.E+01
Tle5	22 kDa	2	0.99253955	1.8.E+04	1.5.E+06	8.2.E+01
Sf3b6	15 kDa	4	0.99999996	1.1.E+06	8.6.E+07	8.2.E+01
Kif18a	101 kDa	4	0.99732976	2.3.E+04	1.9.E+06	8.2.E+01
Gpt2	58 kDa	4	0.99856123	2.2.E+04	1.8.E+06	8.1.E+01
Slc30a9	63 kDa	1	0.99875171	5.1.E+03	4.2.E+05	8.1.E+01
Mfap1a	52 kDa	7	0.99998058	4.2.E+05	3.4.E+07	8.1.E+01
Mfap1a	52 kDa	7	0.99998058	4.2.E+05	3.4.E+07	8.1.E+01
Mfap1b	52 kDa	7	0.99998058	4.2.E+05	3.4.E+07	8.1.E+01
Wdr83	34 kDa	4	0.99889449	8.3.E+03	6.7.E+05	8.0.E+01
Mak16	35 kDa	7	0.99999948	7.3.E+05	5.9.E+07	8.0.E+01
Ccdc130	44 kDa	9	0.99999908	1.5.E+05	1.2.E+07	7.9.E+01
Fancm	226 kDa	3	0.99769219	4.4.E+03	3.5.E+05	7.9.E+01
Traf4	54 kDa	23	0.99999975	2.2.E+06	1.7.E+08	7.9.E+01
Nfe2l2	67 kDa	4	0.99592462	2.0.E+04	1.6.E+06	7.9.E+01
Hgh1	43 kDa	3	0.99897491	2.6.E+04	2.1.E+06	7.8.E+01
Pdcd11	208 kDa	57	0.99999957	8.4.E+06	6.6.E+08	7.8.E+01
Tmem245	97 kDa	2	0.99796455	9.0.E+03	7.0.E+05	7.8.E+01
Snip1	44 kDa	9	0.99998684	1.0.E+05	7.9.E+06	7.7.E+01
Crnkl1	83 kDa	26	0.99993226	2.9.E+06	2.2.E+08	7.7.E+01
Aqr	170 kDa	47	0.99999933	2.6.E+06	2.0.E+08	7.7.E+01
Znf335	146 kDa	4	0.9896176	1.4.E+04	1.0.E+06	7.7.E+01
Cmss1	32 kDa	8	0.99998514	2.7.E+05	2.0.E+07	7.7.E+01
Znf746	70 kDa	1	0.99171315	1.2.E+04	9.6.E+05	7.7.E+01

Zfand3	25 kDa	1	0.99140585	3.2.E+03	2.4.E+05	7.7.E+01
Egln1	43 kDa	7	0.99629279	3.2.E+04	2.4.E+06	7.6.E+01
Snw1	61 kDa	16	0.99999926	1.6.E+06	1.2.E+08	7.6.E+01
Cystm1	11 kDa	1	0.9850159	3.3.E+03	2.5.E+05	7.6.E+01
Sik1	85 kDa	3	0.99947411	4.3.E+03	3.2.E+05	7.6.E+01
Srsf9	26 kDa	12	0.99987624	8.8.E+05	6.7.E+07	7.5.E+01
Gtpbp4	74 kDa	28	0.99999959	7.9.E+06	5.9.E+08	7.4.E+01
Mrpl11	21 kDa	5	0.99894295	1.9.E+05	1.4.E+07	7.3.E+01
Hdx	77 kDa	10	0.9998261	1.3.E+05	9.7.E+06	7.3.E+01
Prpf19	55 kDa	11	1	3.1.E+06	2.2.E+08	7.3.E+01
Myh10	229 kDa	97	0.99999998	3.7.E+07	2.7.E+09	7.3.E+01
Ddx56	61 kDa	12	0.9999988	3.0.E+06	2.2.E+08	7.2.E+01
Cgnl1	148 kDa	52	0.99999999	2.5.E+06	1.8.E+08	7.2.E+01
Lire1	41 kDa	6	0.99980293	2.2.E+05	1.6.E+07	7.2.E+01
Prpf8	274 kDa	77	0.99999999	1.2.E+07	8.6.E+08	7.2.E+01
Eftud2	109 kDa	35	0.99999984	7.8.E+06	5.6.E+08	7.2.E+01
Pnn	82 kDa	21	0.9999987	2.0.E+06	1.4.E+08	7.2.E+01
Stk11	49 kDa	4	0.99817618	8.6.E+03	6.2.E+05	7.1.E+01
Bclaf1	106 kDa	28	1	1.4.E+07	9.7.E+08	7.1.E+01
Hnrnpc	34 kDa	8	0.9998546	3.0.E+07	2.1.E+09	7.0.E+01
Myo1b	129 kDa	50	0.99999994	2.9.E+07	2.1.E+09	7.0.E+01
Dkc1	57 kDa	17	1	1.5.E+06	1.0.E+08	7.0.E+01
Rbmxl1	42 kDa	8	0.99999958	3.6.E+06	2.5.E+08	6.9.E+01
Brix1	41 kDa	10	0.99999999	2.4.E+06	1.7.E+08	6.9.E+01
Rbbp8	101 kDa	2	0.99441512	7.5.E+03	5.2.E+05	6.9.E+01
Vmp1	46 kDa	1	0.99199263	3.8.E+03	2.6.E+05	6.9.E+01
Myo3b	148 kDa	1	0.99848976	3.1.E+06	2.1.E+08	6.9.E+01
Zrsr2	55 kDa	5	0.99999613	1.5.E+04	1.0.E+06	6.8.E+01
Gpn1	42 kDa	2	0.99970136	1.9.E+04	1.3.E+06	6.8.E+01
Nop58	60 kDa	23	0.99999824	1.0.E+07	7.0.E+08	6.8.E+01
Sf3b3	136 kDa	37	0.99999869	5.7.E+06	3.8.E+08	6.8.E+01
Ubxn4	56 kDa	1	0.99743929	4.9.E+03	3.3.E+05	6.7.E+01
Snrnp40	39 kDa	10	0.9999937	2.6.E+06	1.8.E+08	6.7.E+01
Usp42	146 kDa	12	0.99999867	7.2.E+04	4.8.E+06	6.7.E+01
Ppp4r3b	94 kDa	4	0.99175797	5.2.E+03	3.5.E+05	6.7.E+01
Znf451	120 kDa	10	0.99999489	7.6.E+04	5.0.E+06	6.7.E+01
Ing5	28 kDa	2	0.99980292	1.8.E+04	1.2.E+06	6.7.E+01
Zbtb8os	20 kDa	1	0.99851398	1.8.E+04	1.2.E+06	6.6.E+01
Gpatch11	31 kDa	1	0.99496996	4.9.E+03	3.2.E+05	6.6.E+01
Rbm22	47 kDa	13	0.99994247	1.4.E+06	9.1.E+07	6.6.E+01

Adam9	92 kDa	7	0.99971318	3.4.E+04	2.3.E+06	6.6.E+01
Ripor1	132 kDa	3	0.9878817	1.3.E+04	8.5.E+05	6.6.E+01
Snrnp200	245 kDa	83	0.99999996	1.8.E+07	1.2.E+09	6.6.E+01
Tnks2	127 kDa	3	0.9795099	1.3.E+04	8.5.E+05	6.6.E+01
Srfbp1	49 kDa	7	0.9999688	1.9.E+05	1.3.E+07	6.5.E+01
Nop56	64 kDa	26	0.99999755	8.7.E+06	5.7.E+08	6.5.E+01
Ecsit	50 kDa	4	0.99975587	4.5.E+04	2.9.E+06	6.5.E+01
Flcn	64 kDa	5	0.99775589	1.4.E+04	9.0.E+05	6.5.E+01
Ice1	242 kDa	49	0.99999971	2.5.E+06	1.6.E+08	6.5.E+01
Cdk8	53 kDa	3	0.99532156	9.6.E+03	6.2.E+05	6.4.E+01
Ninl	158 kDa	4	0.99976772	2.0.E+04	1.3.E+06	6.4.E+01
Rasip1	104 kDa	1	0.99151322	6.7.E+02	4.3.E+04	6.4.E+01
Hsbp1	9 kDa	2	0.99747662	3.2.E+05	2.1.E+07	6.3.E+01
Ik	66 kDa	11	0.99999882	1.1.E+06	7.1.E+07	6.3.E+01
Snrpb2	25 kDa	5	0.99999558	1.3.E+06	8.3.E+07	6.3.E+01
Cln8	33 kDa	2	0.99918867	2.6.E+04	1.6.E+06	6.3.E+01
Btbd1	53 kDa	3	0.99750469	1.5.E+04	9.6.E+05	6.2.E+01
Med6	28 kDa	3	0.99496591	1.7.E+04	1.1.E+06	6.2.E+01
Noc4l	59 kDa	11	0.99999983	5.8.E+05	3.6.E+07	6.2.E+01
Pex13	45 kDa	2	0.9991948	5.6.E+03	3.5.E+05	6.2.E+01
Irf6	53 kDa	1	0.9895786	2.7.E+03	1.7.E+05	6.2.E+01
Med28	20 kDa	2	0.99944609	7.4.E+03	4.6.E+05	6.2.E+01
Sf3b1	146 kDa	41	0.99999995	5.5.E+06	3.4.E+08	6.2.E+01
Pgm3	59 kDa	2	0.99980614	6.8.E+03	4.2.E+05	6.2.E+01
Ssna1	14 kDa	2	0.99976043	3.8.E+04	2.3.E+06	6.2.E+01
Nkrf	78 kDa	19	0.9998495	4.3.E+05	2.7.E+07	6.2.E+01
N4bp3	60 kDa	7	0.99999736	1.2.E+05	7.2.E+06	6.2.E+01
Yipf5	28 kDa	2	0.99995618	3.5.E+04	2.1.E+06	6.1.E+01
Plrg1	57 kDa	17	0.99999998	2.8.E+05	1.7.E+07	6.1.E+01
Myl7	19 kDa	1	0.9830442	1.5.E+05	9.5.E+06	6.1.E+01
Diablo	27 kDa	2	0.99333899	1.0.E+04	6.3.E+05	6.1.E+01
Tmem101	29 kDa	1	0.9998075	1.0.E+04	6.1.E+05	6.1.E+01
Dnajc21	62 kDa	5	0.9969573	3.4.E+04	2.1.E+06	6.1.E+01
Ddx10	101 kDa	27	0.99999314	2.1.E+06	1.3.E+08	6.0.E+01
Amd2	38 kDa	2	0.9913528	1.2.E+04	7.3.E+05	6.0.E+01
Amd2	38 kDa	2	0.9913528	1.2.E+04	7.3.E+05	6.0.E+01
Amd1	38 kDa	2	0.9913528	1.2.E+04	7.3.E+05	6.0.E+01
Myo1e	127 kDa	32	0.99999839	4.0.E+06	2.4.E+08	6.0.E+01
Tmem177	34 kDa	2	0.99081411	1.6.E+04	9.7.E+05	6.0.E+01
Son	266 kDa	39	0.99999982	5.2.E+06	3.1.E+08	6.0.E+01

Sf3b4	44 kDa	4	0.99999186	1.7.E+04	1.0.E+06	6.0.E+01
Rbm17	45 kDa	8	0.99998606	1.5.E+05	8.9.E+06	6.0.E+01
Wbp4	42 kDa	3	0.9979138	2.0.E+04	1.2.E+06	6.0.E+01
Usf2	37 kDa	2	0.996652	2.0.E+04	1.2.E+06	6.0.E+01
Zkscan3	63 kDa	7	0.9998731	3.3.E+04	1.9.E+06	5.9.E+01
Myl12b	20 kDa	9	0.99999996	1.5.E+07	8.8.E+08	5.9.E+01
Pard6b	41 kDa	2	0.99750786	2.9.E+04	1.7.E+06	5.9.E+01
Abt1	31 kDa	10	0.99998655	1.5.E+06	8.7.E+07	5.9.E+01
Arhgap23	162 kDa	39	0.99998477	7.6.E+05	4.4.E+07	5.9.E+01
Bbs4	58 kDa	1	0.99809222	5.1.E+03	3.0.E+05	5.9.E+01
Yipf3	38 kDa	1	0.9889182	3.5.E+03	2.1.E+05	5.8.E+01
Akt1s1	27 kDa	1	0.9856327	7.0.E+03	4.1.E+05	5.8.E+01
Arid1b	238 kDa	5	0.99355608	1.2.E+04	6.8.E+05	5.8.E+01
Capza1	33 kDa	7	0.99985507	1.1.E+06	6.5.E+07	5.8.E+01
Ovca2	24 kDa	2	0.9976967	9.6.E+03	5.5.E+05	5.8.E+01
Slc25a28	39 kDa	3	0.9989565	2.2.E+04	1.2.E+06	5.7.E+01
Capzb	31 kDa	13	0.99998376	7.6.E+06	4.4.E+08	5.7.E+01
Rbm19	106 kDa	24	0.99999978	9.7.E+05	5.5.E+07	5.7.E+01
Dnajc19	12 kDa	1	0.99991914	9.9.E+03	5.6.E+05	5.7.E+01
Exosc1	21 kDa	2	0.99601242	1.6.E+04	9.1.E+05	5.7.E+01
Eif6	27 kDa	5	0.99999955	3.3.E+06	1.9.E+08	5.7.E+01
Scamp4	25 kDa	1	0.99715546	3.1.E+04	1.8.E+06	5.7.E+01
Washc2	145 kDa	8	0.99996555	5.7.E+04	3.2.E+06	5.6.E+01
Fbxo38	134 kDa	3	0.99390001	1.0.E+04	5.7.E+05	5.6.E+01
Pex16	39 kDa	2	0.99921292	4.5.E+04	2.5.E+06	5.6.E+01
Bag2	23 kDa	3	0.99796924	1.0.E+04	5.6.E+05	5.6.E+01
Cttnbp2nl	70 kDa	2	0.9796758	8.1.E+03	4.5.E+05	5.6.E+01
Ctdsp2	31 kDa	1	0.99183266	1.2.E+04	6.7.E+05	5.6.E+01
Thnsl1	83 kDa	2	0.99551352	1.0.E+04	5.8.E+05	5.6.E+01
Parp9	97 kDa	4	0.99816457	8.8.E+03	4.9.E+05	5.6.E+01
Cspp1	138 kDa	3	0.99900225	7.7.E+03	4.3.E+05	5.6.E+01
Nsun4	43 kDa	6	0.9995017	8.4.E+04	4.7.E+06	5.6.E+01
Sf3a3	59 kDa	12	0.99999759	1.3.E+06	7.1.E+07	5.6.E+01
Srp9	10 kDa	1	0.99972976	3.8.E+04	2.1.E+06	5.6.E+01
Amdhd2	44 kDa	1	0.99202023	7.0.E+03	3.9.E+05	5.5.E+01
Esf1	98 kDa	22	0.99996142	1.0.E+06	5.7.E+07	5.5.E+01
Exosc4	26 kDa	5	0.99929362	4.4.E+04	2.4.E+06	5.5.E+01
Plekhg5	119 kDa	2	0.99002331	1.0.E+04	5.5.E+05	5.5.E+01
Pno1	27 kDa	7	0.99999992	3.6.E+05	2.0.E+07	5.5.E+01
Ccdc9	61 kDa	4	0.99820346	9.9.E+04	5.4.E+06	5.5.E+01

Myl6	17 kDa	7	0.9999919	4.0.E+07	2.2.E+09	5.5.E+01
Six4	82 kDa	2	0.9945606	1.6.E+04	8.6.E+05	5.4.E+01
Exosc5	25 kDa	2	0.99908987	2.2.E+04	1.2.E+06	5.4.E+01
Cdca7l	50 kDa	4	0.9995074	1.0.E+05	5.7.E+06	5.4.E+01
Slc43a1	63 kDa	1	0.99029566	9.5.E+03	5.2.E+05	5.4.E+01
Med18	24 kDa	1	0.9929938	1.3.E+04	7.3.E+05	5.4.E+01
Erlin1	39 kDa	3	0.99982874	5.7.E+03	3.1.E+05	5.4.E+01
Usp38	116 kDa	5	0.99909959	2.3.E+04	1.3.E+06	5.4.E+01
Akip1	24 kDa	3	0.9997723	4.2.E+04	2.2.E+06	5.4.E+01
Mns1	60 kDa	2	0.99170756	9.3.E+03	5.0.E+05	5.4.E+01
Glyr1	60 kDa	14	0.99999995	3.1.E+06	1.6.E+08	5.3.E+01
Cog4	89 kDa	10	0.99995094	1.1.E+05	5.6.E+06	5.3.E+01
-	26 kDa	1	0.99403252	6.8.E+03	3.6.E+05	5.3.E+01
Rchy1	30 kDa	5	0.99982515	2.0.E+04	1.1.E+06	5.3.E+01
Actbl2	42 kDa	12	0.99834436	1.5.E+08	7.7.E+09	5.3.E+01
Srsf2	25 kDa	2	0.99999953	1.7.E+05	9.1.E+06	5.3.E+01
Ppil1	18 kDa	3	0.99999951	1.6.E+06	8.2.E+07	5.3.E+01
Gar1	23 kDa	5	0.99981109	3.3.E+06	1.7.E+08	5.2.E+01
Znf414	32 kDa	2	0.99999347	1.7.E+04	9.1.E+05	5.2.E+01
Zmpste24	55 kDa	2	0.976813	3.0.E+03	1.6.E+05	5.2.E+01
Pgm2	69 kDa	4	0.99913249	3.5.E+04	1.8.E+06	5.2.E+01
Jcad	145 kDa	27	0.99999986	6.0.E+05	3.1.E+07	5.2.E+01
Gpkow	54 kDa	3	0.99457258	3.8.E+04	2.0.E+06	5.2.E+01
Tjap1	59 kDa	2	0.99948182	4.1.E+03	2.2.E+05	5.2.E+01
Cdc5l	92 kDa	28	0.999999	2.6.E+06	1.3.E+08	5.2.E+01
Nhp2	17 kDa	4	0.99999016	3.2.E+05	1.7.E+07	5.2.E+01
Mrto4	28 kDa	15	0.99999999	3.5.E+06	1.8.E+08	5.1.E+01
Pgam5	32 kDa	10	0.99999681	5.9.E+05	3.0.E+07	5.1.E+01
Srsf6	39 kDa	7	0.99960093	1.4.E+06	6.9.E+07	5.1.E+01
Becn1	52 kDa	3	0.99405186	2.1.E+04	1.1.E+06	5.1.E+01
Bbc3	21 kDa	1	0.99890601	3.6.E+03	1.8.E+05	5.1.E+01
Myh9	226 kDa	100	0.99999998	1.4.E+08	7.3.E+09	5.1.E+01
Arpc1b	41 kDa	12	0.99999991	3.5.E+05	1.8.E+07	5.1.E+01
Commd10	23 kDa	4	0.99966845	3.7.E+04	1.9.E+06	5.1.E+01
Syf2	29 kDa	9	0.9996787	6.7.E+05	3.4.E+07	5.1.E+01
Capza2	33 kDa	11	0.9999964	4.3.E+06	2.2.E+08	5.0.E+01
Yrdc	29 kDa	2	0.99715431	2.4.E+04	1.2.E+06	5.0.E+01
Tmcc1	72 kDa	2	0.99759662	1.3.E+04	6.7.E+05	5.0.E+01
Slc7a6os	35 kDa	5	0.99993672	3.7.E+04	1.9.E+06	5.0.E+01
N6amt1	23 kDa	2	0.99988483	1.0.E+04	5.0.E+05	5.0.E+01

Acin1	151 kDa	16	0.99999529	7.0.E+05	3.5.E+07	5.0.E+01
Uncx	54 kDa	1	0.99687976	4.2.E+04	2.1.E+06	5.0.E+01
Uncx	54 kDa	1	0.99687976	4.2.E+04	2.1.E+06	5.0.E+01
Otp	34 kDa	1	0.99687976	4.2.E+04	2.1.E+06	5.0.E+01
Arx	58 kDa	1	0.99687976	4.2.E+04	2.1.E+06	5.0.E+01
Alx4	43 kDa	1	0.99687976	4.2.E+04	2.1.E+06	5.0.E+01
Rax	36 kDa	1	0.99687976	4.2.E+04	2.1.E+06	5.0.E+01
Phox2b	32 kDa	1	0.99687976	4.2.E+04	2.1.E+06	5.0.E+01
Alx3	37 kDa	1	0.99687976	4.2.E+04	2.1.E+06	5.0.E+01
Lhx3	44 kDa	1	0.99687976	4.2.E+04	2.1.E+06	5.0.E+01
Lhx4	43 kDa	1	0.99687976	4.2.E+04	2.1.E+06	5.0.E+01
Prrx1	27 kDa	1	0.99687976	4.2.E+04	2.1.E+06	5.0.E+01
Shox2	35 kDa	1	0.99687976	4.2.E+04	2.1.E+06	5.0.E+01
Prrx2	26 kDa	1	0.99687976	4.2.E+04	2.1.E+06	5.0.E+01
Phox2a	29 kDa	1	0.99687976	4.2.E+04	2.1.E+06	5.0.E+01
Drgx	29 kDa	1	0.99687976	4.2.E+04	2.1.E+06	5.0.E+01
Alx1	37 kDa	1	0.99687976	4.2.E+04	2.1.E+06	5.0.E+01
Nop10	8 kDa	3	0.99999983	1.8.E+05	9.2.E+06	5.0.E+01
Plekha7	127 kDa	33	0.99999998	4.7.E+06	2.3.E+08	5.0.E+01
Xab2	100 kDa	27	0.99999952	2.5.E+06	1.2.E+08	5.0.E+01
Spr	28 kDa	3	0.99945634	2.6.E+04	1.3.E+06	4.9.E+01
Ptges	17 kDa	2	0.99919657	1.5.E+04	7.6.E+05	4.9.E+01
Ppm1j	56 kDa	4	0.99965494	2.9.E+04	1.4.E+06	4.9.E+01
Leng1	30 kDa	4	0.99993197	6.1.E+04	3.0.E+06	4.9.E+01
Phf5a	12 kDa	2	0.99985572	1.0.E+06	5.1.E+07	4.9.E+01
Cep44	43 kDa	6	0.99937671	5.6.E+04	2.8.E+06	4.9.E+01
Rgl2	84 kDa	1	0.99460864	6.2.E+03	3.1.E+05	4.9.E+01
Spdl1	70 kDa	1	0.99926918	5.0.E+03	2.4.E+05	4.9.E+01
Zrsr1	51 kDa	6	0.99954921	3.8.E+04	1.9.E+06	4.9.E+01
Cep89	90 kDa	12	0.99902969	1.4.E+05	6.9.E+06	4.9.E+01
Srsf7	31 kDa	8	0.99999941	9.0.E+06	4.4.E+08	4.9.E+01
Desi1	18 kDa	1	0.99652743	1.3.E+04	6.5.E+05	4.9.E+01
Prmt6	42 kDa	2	0.99900846	1.1.E+04	5.4.E+05	4.9.E+01
Sap18	18 kDa	8	0.99999327	1.7.E+05	8.5.E+06	4.9.E+01
Ess2	53 kDa	14	0.99997677	5.6.E+05	2.7.E+07	4.9.E+01
Myl6b	23 kDa	7	0.99982067	2.7.E+05	1.3.E+07	4.9.E+01
Dhx9	149 kDa	38	0.9999998	2.2.E+07	1.1.E+09	4.8.E+01
Asap1	127 kDa	5	0.99842973	1.3.E+04	6.1.E+05	4.8.E+01
FRRS1	66 kDa	2	0.9817298	2.7.E+04	1.3.E+06	4.8.E+01
Hgsnat	73 kDa	2	0.99926442	1.8.E+04	8.4.E+05	4.8.E+01

Ptpn3	104 kDa	5	0.99293665	2.6.E+04	1.3.E+06	4.8.E+01
Dcaf13	51 kDa	13	0.99998465	3.4.E+06	1.6.E+08	4.8.E+01
Syne4	42 kDa	4	0.99992841	9.5.E+04	4.5.E+06	4.7.E+01
Scd1	41 kDa	1	0.9833449	2.2.E+04	1.1.E+06	4.7.E+01
Nol6	129 kDa	22	0.99999827	1.0.E+06	4.7.E+07	4.7.E+01
Myh11	227 kDa	20	0.99320376	3.1.E+06	1.5.E+08	4.7.E+01
Slc25a40	38 kDa	3	0.99921639	3.7.E+04	1.7.E+06	4.7.E+01
Jak1	133 kDa	6	0.99647184	4.6.E+04	2.1.E+06	4.7.E+01
Dvl3	78 kDa	5	0.9995324	1.9.E+04	8.7.E+05	4.7.E+01
Prkag2	63 kDa	4	0.99519419	1.1.E+04	5.0.E+05	4.6.E+01
Tchp	61 kDa	4	0.99631814	3.6.E+04	1.7.E+06	4.6.E+01
Hmga1	12 kDa	2	0.99997252	2.9.E+06	1.3.E+08	4.6.E+01
Tamm41	38 kDa	4	0.99929525	2.3.E+05	1.1.E+07	4.6.E+01
Cog2	82 kDa	6	0.99984698	3.7.E+04	1.7.E+06	4.6.E+01
Wdr77	37 kDa	4	0.99996894	2.4.E+05	1.1.E+07	4.6.E+01
Actb	42 kDa	24	0.99999999	2.9.E+08	1.3.E+10	4.6.E+01
Actb	42 kDa	24	0.99999999	2.9.E+08	1.3.E+10	4.6.E+01
Actg1	42 kDa	24	0.99999999	2.9.E+08	1.3.E+10	4.6.E+01
Acacb	276 kDa	8	0.99548808	3.4.E+04	1.6.E+06	4.6.E+01
Ubr2	199 kDa	11	0.999524	1.4.E+05	6.4.E+06	4.6.E+01
Smyd2	50 kDa	3	0.99917072	2.3.E+04	1.0.E+06	4.6.E+01
Exosc2	33 kDa	4	0.99671988	8.5.E+04	3.9.E+06	4.5.E+01
Traf7	66 kDa	3	0.99591414	1.9.E+04	8.6.E+05	4.5.E+01
Ppp2r3d	56 kDa	6	0.99955917	1.2.E+05	5.6.E+06	4.5.E+01
Tut1	95 kDa	3	0.99999482	2.2.E+04	9.8.E+05	4.5.E+01
Micu1	54 kDa	1	0.99831683	6.6.E+03	2.9.E+05	4.5.E+01
Def6	73 kDa	12	0.99871114	4.6.E+05	2.0.E+07	4.5.E+01
Sart1	91 kDa	16	0.99999999	2.5.E+05	1.1.E+07	4.5.E+01
Noc2l	85 kDa	12	0.99991676	8.5.E+05	3.8.E+07	4.5.E+01
Adpgk	54 kDa	4	0.99922011	5.0.E+04	2.2.E+06	4.4.E+01
Kifc3	93 kDa	9	0.99791783	9.0.E+04	4.0.E+06	4.4.E+01
Dzip1l	88 kDa	5	0.99998392	4.8.E+04	2.1.E+06	4.4.E+01
Nfkb2	97 kDa	9	0.99975383	4.5.E+04	2.0.E+06	4.4.E+01
Dhx15	91 kDa	24	0.99999997	5.2.E+06	2.3.E+08	4.4.E+01
Pak1ip1	42 kDa	7	0.99998989	4.3.E+05	1.9.E+07	4.4.E+01
Ubl7	40 kDa	2	0.9939977	1.9.E+04	8.3.E+05	4.4.E+01
Pycr3	29 kDa	4	0.99786019	3.3.E+04	1.5.E+06	4.4.E+01
Topors	117 kDa	3	0.99687475	4.8.E+04	2.1.E+06	4.4.E+01
Paat	49 kDa	1	0.9970962	1.6.E+04	7.1.E+05	4.4.E+01
Depdc5	180 kDa	4	0.99753118	1.6.E+04	7.1.E+05	4.4.E+01

Irak3	68 kDa	3	0.99232469	2.5.E+04	1.1.E+06	4.4.E+01
Spart	73 kDa	5	0.99954564	3.8.E+04	1.7.E+06	4.4.E+01
Nat10	115 kDa	37	0.99999999	7.5.E+06	3.3.E+08	4.3.E+01
Tkfc	60 kDa	8	0.99985983	9.4.E+04	4.1.E+06	4.3.E+01
Zwint	29 kDa	4	0.99995967	8.3.E+04	3.6.E+06	4.3.E+01
Cdc42bpg	172 kDa	9	0.99946375	7.9.E+04	3.4.E+06	4.3.E+01
Cetn2	20 kDa	4	0.99978231	3.1.E+04	1.3.E+06	4.3.E+01
Myh14	229 kDa	50	0.99999492	8.9.E+06	3.9.E+08	4.3.E+01
Tm7sf3	63 kDa	4	0.99981845	1.1.E+05	4.6.E+06	4.3.E+01
Rab4b	24 kDa	5	0.99684597	3.8.E+04	1.6.E+06	4.3.E+01
Ino80c	20 kDa	4	0.99897633	4.7.E+04	2.0.E+06	4.3.E+01
Mysm1	93 kDa	5	0.99903282	2.5.E+04	1.1.E+06	4.3.E+01
Mtfr2	41 kDa	4	0.99984882	2.6.E+04	1.1.E+06	4.3.E+01
Mcrs1	52 kDa	6	0.99988845	8.0.E+04	3.4.E+06	4.3.E+01
Aatf	59 kDa	14	0.99998337	2.5.E+06	1.1.E+08	4.3.E+01
Tbc1d13	46 kDa	8	0.99859338	8.1.E+04	3.5.E+06	4.3.E+01
Adar	130 kDa	5	0.9847364	1.9.E+04	7.9.E+05	4.3.E+01
Lclat1	44 kDa	3	0.99597346	1.1.E+04	4.8.E+05	4.2.E+01
Rnf114	26 kDa	5	0.99987488	7.8.E+04	3.3.E+06	4.2.E+01
Prkab1	30 kDa	6	0.99983973	2.8.E+04	1.2.E+06	4.2.E+01
Tada1	37 kDa	2	0.9850248	6.8.E+03	2.9.E+05	4.2.E+01
Recql5	108 kDa	3	0.99689008	1.3.E+04	5.4.E+05	4.2.E+01
Pdzd11	16 kDa	4	0.99976291	9.7.E+05	4.1.E+07	4.2.E+01
Mkrn2	47 kDa	3	0.99519274	6.2.E+04	2.6.E+06	4.2.E+01
Ddx27	86 kDa	22	0.99998822	2.7.E+06	1.1.E+08	4.2.E+01
Esrp1	76 kDa	3	0.99962361	1.3.E+04	5.4.E+05	4.2.E+01
Fancl	43 kDa	1	0.9971337	1.1.E+04	4.6.E+05	4.2.E+01
Ecd	72 kDa	6	0.99958376	3.9.E+04	1.6.E+06	4.2.E+01
Fbxo15	49 kDa	3	0.99350375	2.8.E+04	1.1.E+06	4.2.E+01
Snrpb	24 kDa	5	0.99973701	3.9.E+05	1.6.E+07	4.1.E+01
Plekhh1	151 kDa	6	0.99883525	2.7.E+04	1.1.E+06	4.1.E+01
Egfl7	30 kDa	3	0.99986495	4.2.E+04	1.7.E+06	4.1.E+01
Ankrd13a	67 kDa	9	0.99993649	1.8.E+05	7.2.E+06	4.1.E+01
Poc5	61 kDa	15	0.99999916	1.1.E+06	4.4.E+07	4.1.E+01
Kctd3	89 kDa	14	0.9999772	2.1.E+05	8.8.E+06	4.1.E+01
Calcoco1	77 kDa	9	0.99943279	5.5.E+04	2.2.E+06	4.1.E+01
Mapk6	82 kDa	8	0.99966228	6.8.E+04	2.8.E+06	4.1.E+01
Snrpd2	14 kDa	6	0.99991036	3.8.E+06	1.6.E+08	4.1.E+01
Cchcr1	87 kDa	14	0.99993492	2.0.E+05	8.2.E+06	4.1.E+01
Kirrel1	87 kDa	20	0.9999999	2.9.E+06	1.2.E+08	4.1.E+01

Cep83	82 kDa	10	0.99982963	1.6.E+05	6.6.E+06	4.1.E+01
Pus10	60 kDa	5	0.99985794	3.0.E+04	1.2.E+06	4.0.E+01
Armc6	51 kDa	4	0.99975018	5.9.E+04	2.4.E+06	4.0.E+01
Tpst1	42 kDa	3	0.99822944	2.0.E+04	7.9.E+05	4.0.E+01
Akap17b	111 kDa	12	0.9999355	4.3.E+05	1.7.E+07	4.0.E+01
Malsu1	26 kDa	1	0.99963287	1.3.E+04	5.3.E+05	4.0.E+01
Cic	258 kDa	2	0.982834	1.2.E+04	4.8.E+05	4.0.E+01
Clk3	74 kDa	12	0.99999973	5.8.E+05	2.3.E+07	4.0.E+01
Nipsnap2	33 kDa	2	0.9953893	5.2.E+03	2.1.E+05	4.0.E+01
Plk4	104 kDa	3	0.99981572	3.2.E+04	1.3.E+06	4.0.E+01
Fdps	41 kDa	4	0.99901308	2.1.E+05	8.3.E+06	4.0.E+01
Brd7	74 kDa	15	0.99992013	1.3.E+05	5.3.E+06	4.0.E+01
Gemin2	30 kDa	4	0.99999988	8.2.E+04	3.2.E+06	4.0.E+01
Ythdc2	161 kDa	16	0.99961621	1.7.E+05	6.9.E+06	4.0.E+01
Emg1	27 kDa	10	0.99997557	6.6.E+05	2.6.E+07	3.9.E+01
Notch1	271 kDa	2	0.9810314	1.8.E+04	7.1.E+05	3.9.E+01
Pomt1	85 kDa	3	0.99770288	1.7.E+04	6.5.E+05	3.9.E+01
Parp12	80 kDa	4	0.99953809	2.8.E+04	1.1.E+06	3.9.E+01
Med4	30 kDa	4	0.99995316	3.8.E+04	1.5.E+06	3.9.E+01
Stk38l	54 kDa	12	0.99999954	3.7.E+05	1.4.E+07	3.9.E+01
-	35 kDa	2	0.9840805	7.6.E+03	3.0.E+05	3.9.E+01
Agpat1	32 kDa	3	0.9805976	3.3.E+05	1.3.E+07	3.9.E+01
Ap4e1	125 kDa	3	0.9854431	1.1.E+04	4.1.E+05	3.9.E+01
Trnt1	50 kDa	4	0.99772657	6.5.E+04	2.5.E+06	3.9.E+01
Hmga2	12 kDa	1	0.99948728	3.7.E+04	1.4.E+06	3.9.E+01
Surf1	35 kDa	1	0.99907895	1.7.E+04	6.5.E+05	3.9.E+01
Bod1l	327 kDa	10	0.9996639	4.8.E+04	1.8.E+06	3.9.E+01
Phf14	99 kDa	7	0.99975244	6.3.E+04	2.4.E+06	3.9.E+01
Gli2	165 kDa	7	0.99999215	4.0.E+04	1.6.E+06	3.9.E+01
Crabp1	16 kDa	4	0.99968384	6.7.E+04	2.6.E+06	3.8.E+01
Fam3c	25 kDa	2	0.99642185	1.5.E+04	5.8.E+05	3.8.E+01
Bcl3	47 kDa	2	0.99340507	1.8.E+04	7.0.E+05	3.8.E+01
Smyd3	49 kDa	4	0.99965952	3.5.E+04	1.3.E+06	3.8.E+01
Hibadh	35 kDa	2	0.99994007	1.1.E+04	4.2.E+05	3.8.E+01
Rpp40	42 kDa	2	0.99138633	7.6.E+03	2.9.E+05	3.8.E+01
Kiaa1328	70 kDa	7	0.99691356	7.4.E+04	2.8.E+06	3.8.E+01
Ears2	58 kDa	7	0.99998551	3.0.E+04	1.1.E+06	3.8.E+01
Rnpc3	58 kDa	7	0.99690796	1.3.E+05	4.9.E+06	3.8.E+01
Mms22l	141 kDa	9	0.99946229	1.5.E+05	5.7.E+06	3.8.E+01
Rpl10a	25 kDa	10	0.99999987	8.4.E+06	3.2.E+08	3.8.E+01

Rfk	17 kDa	2	0.99754615	3.8.E+04	1.4.E+06	3.8.E+01
Cebpz	120 kDa	30	0.99999999	4.2.E+06	1.6.E+08	3.7.E+01
Phf10	56 kDa	5	0.99988926	7.1.E+04	2.7.E+06	3.7.E+01
Rnf181	19 kDa	2	0.99936809	6.8.E+03	2.5.E+05	3.7.E+01
Cdca5	29 kDa	1	0.9804434	8.7.E+03	3.2.E+05	3.7.E+01
Pkp4	132 kDa	39	0.99999989	5.8.E+06	2.2.E+08	3.7.E+01
Rnf40	114 kDa	9	0.99832784	1.4.E+05	5.3.E+06	3.7.E+01
Mau2	70 kDa	5	0.99659602	1.1.E+04	4.2.E+05	3.7.E+01
Polr3f	36 kDa	1	0.99777979	8.9.E+03	3.3.E+05	3.7.E+01
Gramd1a	81 kDa	2	0.9892646	8.7.E+03	3.2.E+05	3.7.E+01
Agpat2	31 kDa	2	0.99642159	1.9.E+05	7.1.E+06	3.7.E+01
Myg1	43 kDa	5	0.99880929	3.5.E+04	1.3.E+06	3.7.E+01
Dzip3	138 kDa	4	0.99116855	2.1.E+04	7.7.E+05	3.7.E+01
Ten1	18 kDa	1	0.99299278	1.7.E+04	6.4.E+05	3.7.E+01
Nbeal2	302 kDa	4	0.99481979	2.9.E+04	1.1.E+06	3.7.E+01
Unc119	27 kDa	3	0.99928428	1.5.E+04	5.4.E+05	3.7.E+01
Zc3hc1	55 kDa	5	0.99974151	4.9.E+04	1.8.E+06	3.6.E+01
Qtrt1	44 kDa	4	0.99933929	1.1.E+05	3.9.E+06	3.6.E+01
Synrg	140 kDa	3	0.99634811	8.4.E+03	3.1.E+05	3.6.E+01
Ppp2r2d	52 kDa	3	0.99996238	3.0.E+04	1.1.E+06	3.6.E+01
Ube2j1	35 kDa	2	0.99874774	6.2.E+03	2.3.E+05	3.6.E+01
Cdyl	65 kDa	4	0.99839451	3.3.E+04	1.2.E+06	3.6.E+01
Mrps11	20 kDa	1	0.99945436	1.0.E+04	3.7.E+05	3.6.E+01
Polr2j	13 kDa	2	0.9998381	7.1.E+04	2.6.E+06	3.6.E+01
Zdhhc5	78 kDa	2	0.99115045	7.2.E+04	2.6.E+06	3.6.E+01
Elovl5	35 kDa	1	0.99792272	7.7.E+03	2.8.E+05	3.6.E+01
Misp	72 kDa	4	0.9909806	1.3.E+04	4.7.E+05	3.6.E+01
Ubr1	200 kDa	6	0.99998412	3.1.E+04	1.1.E+06	3.6.E+01
Gspt1	69 kDa	11	0.99998962	1.2.E+05	4.3.E+06	3.6.E+01
Exosc9	49 kDa	5	0.99954242	9.6.E+04	3.4.E+06	3.6.E+01
Gprasp2	93 kDa	3	0.99906746	1.3.E+04	4.6.E+05	3.6.E+01
Tgm2	77 kDa	12	0.99985675	9.4.E+05	3.4.E+07	3.6.E+01
Myo1a	119 kDa	7	0.9779186	9.9.E+04	3.5.E+06	3.6.E+01
Ikbkb	87 kDa	3	0.99759927	2.0.E+04	7.3.E+05	3.6.E+01
Ppox	51 kDa	2	0.99930096	4.6.E+04	1.7.E+06	3.6.E+01
Ube3b	123 kDa	9	0.99930087	6.5.E+04	2.3.E+06	3.6.E+01
Knl1	179 kDa	8	0.99946987	7.5.E+04	2.7.E+06	3.6.E+01
Pla2g6	90 kDa	8	0.9997344	1.4.E+05	5.0.E+06	3.6.E+01
Etv3	57 kDa	6	0.99942324	4.6.E+04	1.6.E+06	3.6.E+01
Ca13	30 kDa	1	0.9775178	1.4.E+04	4.9.E+05	3.6.E+01

Tbccd1	63 kDa	12	0.99979724	3.6.E+05	1.3.E+07	3.5.E+01
Tacc1	84 kDa	3	0.99747794	6.7.E+04	2.4.E+06	3.5.E+01
Dcp1a	65 kDa	6	0.99967859	5.0.E+04	1.8.E+06	3.5.E+01
Tmem230	13 kDa	1	0.9829426	2.9.E+04	1.0.E+06	3.5.E+01
Rpf1	40 kDa	9	0.99999913	6.3.E+05	2.2.E+07	3.5.E+01
Slc25a15	33 kDa	3	0.99991016	6.5.E+04	2.3.E+06	3.5.E+01
Marchf7	77 kDa	3	0.99923745	1.6.E+04	5.7.E+05	3.5.E+01
Pard3	149 kDa	21	0.99999761	7.8.E+05	2.7.E+07	3.5.E+01
Fam83h	131 kDa	19	0.99988286	2.8.E+05	9.9.E+06	3.5.E+01
Lsr	66 kDa	10	0.99996653	6.1.E+05	2.1.E+07	3.5.E+01
Camk1d	43 kDa	2	0.99600684	3.5.E+03	1.2.E+05	3.5.E+01
N4bp1	99 kDa	5	0.9995375	4.6.E+04	1.6.E+06	3.5.E+01
Pik3cb	122 kDa	4	0.99914598	3.6.E+04	1.3.E+06	3.5.E+01
Coa7	26 kDa	3	0.99429998	3.5.E+04	1.2.E+06	3.5.E+01
Cep85	85 kDa	9	0.99991528	8.4.E+04	2.9.E+06	3.5.E+01
Prkra	34 kDa	3	0.99997249	4.4.E+04	1.5.E+06	3.5.E+01
Zswim3	79 kDa	4	0.99539793	1.2.E+05	4.1.E+06	3.5.E+01
Chmp2a	25 kDa	1	0.99509999	3.8.E+04	1.3.E+06	3.5.E+01
Snrpe	11 kDa	4	0.99996391	5.7.E+05	2.0.E+07	3.5.E+01
Ppan	53 kDa	13	0.99999988	2.7.E+06	9.4.E+07	3.5.E+01
Drosha	159 kDa	11	0.99969649	2.1.E+05	7.3.E+06	3.5.E+01
Slc35e1	44 kDa	4	0.99955657	3.7.E+04	1.3.E+06	3.5.E+01
Fig4	103 kDa	5	0.99892367	4.5.E+04	1.6.E+06	3.5.E+01
Tbc1d22a	59 kDa	4	0.9943966	2.6.E+04	8.9.E+05	3.4.E+01
Coro2b	55 kDa	6	0.99999586	1.0.E+05	3.5.E+06	3.4.E+01
Radil	120 kDa	4	0.99732258	4.0.E+04	1.4.E+06	3.4.E+01
Gigyf2	149 kDa	15	0.99999676	6.4.E+05	2.2.E+07	3.4.E+01
Arfgap1	45 kDa	2	0.99840437	2.5.E+04	8.7.E+05	3.4.E+01
Mrps7	28 kDa	5	0.99991638	1.4.E+05	4.7.E+06	3.4.E+01
Tdg	47 kDa	2	0.99729245	3.0.E+04	1.0.E+06	3.4.E+01
Ccdc138	78 kDa	3	0.99763675	1.2.E+04	4.2.E+05	3.4.E+01
Tyk2	133 kDa	5	0.99889895	2.7.E+04	9.2.E+05	3.4.E+01
Nfu1	29 kDa	3	0.99997456	2.3.E+05	7.7.E+06	3.4.E+01
Trappc9	128 kDa	4	0.99009852	1.7.E+04	5.8.E+05	3.4.E+01
Fads1	52 kDa	4	0.99999761	2.7.E+05	9.1.E+06	3.4.E+01
Mov10	114 kDa	31	0.9999999	7.1.E+06	2.4.E+08	3.4.E+01
Prkx	40 kDa	2	0.99929344	1.7.E+05	5.7.E+06	3.4.E+01
Rab22a	22 kDa	4	0.9994811	2.5.E+04	8.3.E+05	3.4.E+01
Pphln1	44 kDa	6	0.99936972	6.2.E+04	2.1.E+06	3.4.E+01
Atg5	32 kDa	4	0.99562893	4.6.E+04	1.5.E+06	3.4.E+01

Alg13	129 kDa	7	0.99995941	1.6.E+05	5.5.E+06	3.4.E+01
Gaa	106 kDa	3	0.99841454	2.8.E+04	9.5.E+05	3.4.E+01
Rbm5	92 kDa	10	0.99970273	5.2.E+05	1.7.E+07	3.4.E+01
Ift74	69 kDa	3	0.9850948	2.1.E+04	6.9.E+05	3.3.E+01
Chchd6	30 kDa	7	0.99999578	1.9.E+05	6.3.E+06	3.3.E+01
Sdccag8	83 kDa	8	0.99990595	7.4.E+04	2.5.E+06	3.3.E+01
Paox	55 kDa	6	0.99985029	8.7.E+04	2.9.E+06	3.3.E+01
Hmg20a	40 kDa	3	0.99997308	8.1.E+04	2.7.E+06	3.3.E+01
Thoc1	75 kDa	16	0.9999454	1.3.E+06	4.4.E+07	3.3.E+01
Cep55	54 kDa	23	0.99998698	8.1.E+06	2.7.E+08	3.3.E+01
Stxbp2	66 kDa	12	0.99999884	2.7.E+05	9.0.E+06	3.3.E+01
Nae1	60 kDa	5	0.99900777	6.7.E+04	2.2.E+06	3.3.E+01
Msi1	39 kDa	5	0.99967926	2.9.E+05	9.8.E+06	3.3.E+01
Hsdl2	54 kDa	3	0.9908764	2.0.E+04	6.6.E+05	3.3.E+01
Mrps25	20 kDa	2	0.99061599	1.1.E+05	3.8.E+06	3.3.E+01
Tango6	119 kDa	5	0.99949617	9.0.E+04	3.0.E+06	3.3.E+01
Tesc	25 kDa	1	0.9894659	6.7.E+03	2.2.E+05	3.3.E+01
Scaf1	134 kDa	7	0.99986298	1.2.E+05	3.8.E+06	3.3.E+01
Wtip	42 kDa	3	0.99864573	2.7.E+04	9.0.E+05	3.3.E+01
Wwp1	105 kDa	6	0.99630176	6.0.E+04	2.0.E+06	3.3.E+01
Nr4a1	65 kDa	2	0.9803742	7.1.E+03	2.3.E+05	3.3.E+01
Fnbp4	111 kDa	2	0.99462053	2.8.E+04	9.1.E+05	3.3.E+01
Smtn	100 kDa	2	0.99353837	5.7.E+03	1.9.E+05	3.3.E+01
Ndufaf2	20 kDa	3	0.99965162	1.4.E+05	4.4.E+06	3.3.E+01
Taf10	22 kDa	1	0.99956648	2.1.E+03	6.8.E+04	3.3.E+01
Znf326	65 kDa	16	0.99999921	1.9.E+06	6.3.E+07	3.3.E+01
Mpdz	219 kDa	4	0.99703397	2.6.E+04	8.4.E+05	3.3.E+01
Ccdc6	53 kDa	6	0.99853614	8.6.E+04	2.8.E+06	3.3.E+01
Mrpl53	13 kDa	3	0.99940036	5.2.E+04	1.7.E+06	3.2.E+01
Cert1	71 kDa	7	0.99477274	8.0.E+04	2.6.E+06	3.2.E+01
Wrap73	52 kDa	5	0.99994065	8.4.E+04	2.7.E+06	3.2.E+01
Pxk	65 kDa	4	0.99979612	6.0.E+04	1.9.E+06	3.2.E+01
Vkorc1	18 kDa	1	0.99914968	6.8.E+04	2.2.E+06	3.2.E+01
Rex1bd	19 kDa	2	0.9991957	1.9.E+04	6.1.E+05	3.2.E+01
Col4a2	167 kDa	7	0.99998541	2.3.E+06	7.3.E+07	3.2.E+01
Snrpd3	14 kDa	3	0.99997114	1.4.E+06	4.4.E+07	3.2.E+01
Diexf	89 kDa	22	0.99999799	6.0.E+05	1.9.E+07	3.2.E+01
Dnttip1	37 kDa	2	0.99920032	3.6.E+04	1.2.E+06	3.2.E+01
Mrps33	12 kDa	1	0.99582308	1.5.E+04	4.7.E+05	3.2.E+01
Vps37a	44 kDa	4	0.99761155	8.0.E+04	2.6.E+06	3.2.E+01

Otud7b	92 kDa	3	0.99957316	1.7.E+04	5.5.E+05	3.2.E+01
Clmn	117 kDa	13	0.99989953	3.2.E+05	1.0.E+07	3.2.E+01
Zdhhc18	41 kDa	3	0.99963008	2.6.E+04	8.4.E+05	3.2.E+01
Myo5b	211 kDa	30	0.99993252	5.1.E+05	1.6.E+07	3.2.E+01
Rbfox3	41 kDa	2	0.99969128	4.0.E+05	1.3.E+07	3.2.E+01
Rbfox3	41 kDa	2	0.99969128	4.0.E+05	1.3.E+07	3.2.E+01
Rbfox2	47 kDa	2	0.99969128	4.0.E+05	1.3.E+07	3.2.E+01
Rbfox1	43 kDa	2	0.99969128	4.0.E+05	1.3.E+07	3.2.E+01
Myo1g	117 kDa	27	0.99999422	1.3.E+06	4.2.E+07	3.2.E+01
Ptprk	164 kDa	6	0.99954456	1.4.E+05	4.4.E+06	3.2.E+01
Dhx34	129 kDa	8	0.99915036	3.9.E+04	1.2.E+06	3.2.E+01
Eif4b	69 kDa	3	0.99995922	2.6.E+05	8.1.E+06	3.2.E+01
DSCC1	46 kDa	4	0.99895831	5.5.E+04	1.8.E+06	3.2.E+01
Nefh	117 kDa	7	0.99762702	2.2.E+04	7.0.E+05	3.2.E+01
Hpdl	40 kDa	1	0.99579103	1.3.E+04	4.1.E+05	3.2.E+01
Mrpl40	24 kDa	4	0.9971431	1.2.E+05	3.9.E+06	3.2.E+01
Nr2c2ap	16 kDa	1	0.99805614	3.2.E+04	1.0.E+06	3.2.E+01
Ciao2a	18 kDa	2	0.9870063	2.0.E+04	6.2.E+05	3.2.E+01
-	38 kDa	1	0.99348183	2.3.E+04	7.4.E+05	3.2.E+01
Taf4b	90 kDa	2	0.99368942	1.2.E+05	3.9.E+06	3.1.E+01
Nek7	35 kDa	4	0.99821502	5.3.E+04	1.7.E+06	3.1.E+01
Ctnnb1	85 kDa	21	0.99999729	5.2.E+06	1.6.E+08	3.1.E+01
Srpk2	77 kDa	10	0.99902184	8.9.E+04	2.8.E+06	3.1.E+01
Phlda3	14 kDa	2	0.9888653	1.4.E+04	4.4.E+05	3.1.E+01
Keap1	70 kDa	7	0.99934285	7.2.E+04	2.3.E+06	3.1.E+01
Miip	43 kDa	4	0.99851653	4.8.E+04	1.5.E+06	3.1.E+01
Spry2	35 kDa	1	0.99918423	7.0.E+04	2.2.E+06	3.1.E+01
Pigk	45 kDa	4	0.99900958	1.3.E+05	4.0.E+06	3.1.E+01
Myo6	146 kDa	19	0.99998805	6.4.E+05	2.0.E+07	3.1.E+01
Cenpx	9 kDa	1	0.9790972	1.3.E+04	4.1.E+05	3.1.E+01
Znf513	58 kDa	2	0.99973753	2.3.E+04	7.2.E+05	3.1.E+01
Snx16	39 kDa	1	0.99683366	7.3.E+03	2.3.E+05	3.1.E+01
Tdrd3	82 kDa	7	0.99812968	9.0.E+04	2.8.E+06	3.1.E+01
Fancg	69 kDa	4	0.99428582	3.8.E+04	1.2.E+06	3.1.E+01
Armh3	79 kDa	1	0.99511755	1.4.E+04	4.3.E+05	3.1.E+01
Fbxw8	68 kDa	6	0.99960256	7.1.E+04	2.2.E+06	3.1.E+01
Tdrp	20 kDa	4	0.99946301	1.9.E+04	5.8.E+05	3.1.E+01
Dbnl	49 kDa	10	0.99993865	5.7.E+05	1.8.E+07	3.1.E+01
Sf3a1	89 kDa	22	0.9999997	8.2.E+06	2.5.E+08	3.1.E+01
Kcnn2	92 kDa	1	0.99941497	1.2.E+04	3.7.E+05	3.1.E+01

Strip1	96 kDa	3	0.99561533	5.1.E+04	1.6.E+06	3.1.E+01
Irak1bp1	29 kDa	7	0.99987178	7.9.E+04	2.4.E+06	3.1.E+01
Nkap	47 kDa	2	0.99805764	2.0.E+04	6.2.E+05	3.1.E+01
Cherp	106 kDa	12	0.99996254	9.0.E+05	2.7.E+07	3.1.E+01
Otud6b	34 kDa	4	0.9997859	8.4.E+04	2.6.E+06	3.1.E+01
Hars2	57 kDa	5	0.99792428	4.7.E+04	1.4.E+06	3.1.E+01
Marchf6	102 kDa	1	0.99172597	6.1.E+03	1.9.E+05	3.0.E+01
Haus7	41 kDa	4	0.9861971	5.4.E+04	1.7.E+06	3.0.E+01
Cep135	133 kDa	31	0.99998624	1.3.E+06	3.9.E+07	3.0.E+01
Sec16a	254 kDa	9	0.99992456	1.4.E+05	4.2.E+06	3.0.E+01
Bfar	53 kDa	1	0.99358529	1.0.E+04	3.0.E+05	3.0.E+01
Map3k7	64 kDa	9	0.99927056	1.2.E+05	3.7.E+06	3.0.E+01
Prr12	212 kDa	4	0.99976337	6.2.E+04	1.9.E+06	3.0.E+01
Rhbdf2	93 kDa	2	0.99406752	4.5.E+04	1.4.E+06	3.0.E+01
Ppp4r1	106 kDa	11	0.99994762	1.1.E+05	3.4.E+06	3.0.E+01
Rabl2	26 kDa	5	0.99947923	7.0.E+04	2.1.E+06	3.0.E+01
Tpm1	33 kDa	10	0.99967694	2.9.E+06	8.7.E+07	3.0.E+01
Pde12	68 kDa	4	0.99921054	1.1.E+05	3.5.E+06	3.0.E+01
Ccnf	87 kDa	2	0.99287899	7.0.E+03	2.1.E+05	3.0.E+01
Kif14	186 kDa	54	0.99999977	1.2.E+07	3.5.E+08	3.0.E+01
Ciao3	53 kDa	1	0.9777001	1.8.E+04	5.4.E+05	3.0.E+01
Znf511	26 kDa	3	0.9769198	4.5.E+04	1.4.E+06	3.0.E+01
Specc1	118 kDa	5	0.99954826	4.2.E+04	1.3.E+06	3.0.E+01
Zbtb1	82 kDa	8	0.99997829	9.4.E+04	2.8.E+06	3.0.E+01
Dhodh	43 kDa	5	0.99986203	5.9.E+04	1.8.E+06	3.0.E+01
Slc25a10	32 kDa	7	0.99988456	2.4.E+05	7.2.E+06	3.0.E+01
Nprl3	64 kDa	3	0.99936336	1.9.E+04	5.7.E+05	3.0.E+01
Pex10	37 kDa	4	0.9885047	5.3.E+04	1.6.E+06	3.0.E+01
Slmap	97 kDa	8	0.9998023	6.0.E+04	1.8.E+06	3.0.E+01
Dus1l	54 kDa	2	0.99053691	2.8.E+03	8.4.E+04	3.0.E+01
Trp53inp1	27 kDa	1	0.99818668	1.8.E+04	5.5.E+05	3.0.E+01
Stard3nl	27 kDa	1	0.99794375	2.1.E+04	6.4.E+05	3.0.E+01
Wrap53	58 kDa	2	0.99562754	1.4.E+04	4.1.E+05	3.0.E+01
Patl1	87 kDa	6	0.99922657	8.0.E+04	2.4.E+06	3.0.E+01
Esco1	95 kDa	8	0.99767768	5.0.E+04	1.5.E+06	3.0.E+01
Iqsec1	108 kDa	11	0.998583	1.8.E+05	5.5.E+06	3.0.E+01
Ighmbp2	109 kDa	3	0.99280774	2.1.E+04	6.1.E+05	3.0.E+01
Brd2	88 kDa	6	0.99988193	7.5.E+04	2.2.E+06	3.0.E+01
Fbxo21	72 kDa	2	0.9879585	1.4.E+04	4.3.E+05	3.0.E+01
Lamtor5	10 kDa	2	0.99586037	2.1.E+04	6.3.E+05	3.0.E+01

Slc25a19	36 kDa	1	0.9822494	1.3.E+04	3.9.E+05	3.0.E+01
Sar1b	22 kDa	2	0.99945363	1.3.E+05	3.9.E+06	3.0.E+01
Poglut2	58 kDa	4	0.99673079	4.1.E+04	1.2.E+06	3.0.E+01
Traf6	60 kDa	4	0.99937584	3.6.E+04	1.1.E+06	3.0.E+01
Cdkal1	65 kDa	10	0.99987613	4.4.E+05	1.3.E+07	3.0.E+01
Ccpg1	86 kDa	1	0.99152099	6.3.E+03	1.9.E+05	3.0.E+01
Loxl2	87 kDa	3	0.99999218	7.4.E+03	2.2.E+05	3.0.E+01
Gpatch8	165 kDa	10	0.99979587	1.8.E+05	5.4.E+06	3.0.E+01
Fkbp15	133 kDa	6	0.99998919	1.1.E+05	3.3.E+06	3.0.E+01
Golph3	34 kDa	3	0.9984556	1.1.E+04	3.2.E+05	2.9.E+01
Grasp	42 kDa	2	0.99609319	7.3.E+03	2.1.E+05	2.9.E+01
Pnp	32 kDa	3	0.99817082	6.2.E+04	1.8.E+06	2.9.E+01
Sh3gl1	42 kDa	6	0.9992543	9.8.E+04	2.9.E+06	2.9.E+01
Gpat4	52 kDa	5	0.99833111	3.9.E+04	1.1.E+06	2.9.E+01
Erbin	157 kDa	9	0.99990339	1.4.E+05	4.0.E+06	2.9.E+01
Top3a	112 kDa	5	0.99917352	3.8.E+04	1.1.E+06	2.9.E+01
Ctu2	56 kDa	5	0.99992556	4.2.E+04	1.2.E+06	2.9.E+01
Cep250	277 kDa	64	0.99999768	3.9.E+06	1.1.E+08	2.9.E+01
Rnf7	13 kDa	1	0.99163405	2.9.E+04	8.5.E+05	2.9.E+01
Rnf25	51 kDa	3	0.99054136	8.3.E+04	2.4.E+06	2.9.E+01
Gramd4	72 kDa	2	0.99482528	1.0.E+04	2.9.E+05	2.9.E+01
Nek9	107 kDa	8	0.99948067	1.4.E+05	4.0.E+06	2.9.E+01
Matr3	95 kDa	29	0.99999994	7.8.E+06	2.3.E+08	2.9.E+01
Mpp7	66 kDa	5	0.99940514	6.0.E+04	1.7.E+06	2.9.E+01
Gcc2	194 kDa	7	0.9773335	3.3.E+04	9.6.E+05	2.9.E+01
Afap1	81 kDa	3	0.99809925	4.1.E+04	1.2.E+06	2.9.E+01
Adam23	92 kDa	1	0.9962595	5.7.E+04	1.6.E+06	2.9.E+01
Tex14	163 kDa	31	0.99999962	4.9.E+06	1.4.E+08	2.9.E+01
Ptdss2	55 kDa	2	0.99971641	1.9.E+05	5.5.E+06	2.9.E+01
Raly	33 kDa	6	0.99999276	2.1.E+07	6.1.E+08	2.9.E+01
Rab31	21 kDa	6	0.99730393	5.8.E+04	1.7.E+06	2.9.E+01
Taf2	127 kDa	5	0.99742224	6.0.E+04	1.7.E+06	2.9.E+01
Hnrnpul2	85 kDa	24	0.99999957	6.9.E+06	2.0.E+08	2.9.E+01
Man1b1	75 kDa	6	0.9995736	4.2.E+04	1.2.E+06	2.9.E+01
Brcc3	33 kDa	3	0.99964715	3.3.E+04	9.6.E+05	2.9.E+01
Unk	88 kDa	3	0.9781887	4.0.E+04	1.2.E+06	2.9.E+01
Tsc1	129 kDa	3	0.99775861	1.9.E+04	5.5.E+05	2.9.E+01
Gtf2h5	8 kDa	1	0.99152981	2.6.E+04	7.5.E+05	2.8.E+01
Mcmbp	73 kDa	9	0.99998937	2.1.E+05	5.8.E+06	2.8.E+01
Slc25a17	34 kDa	6	0.99987627	1.9.E+05	5.4.E+06	2.8.E+01

Cdc25c	50 kDa	1	0.9855371	1.5.E+04	4.3.E+05	2.8.E+01
Uck1	31 kDa	1	0.9790621	3.1.E+04	8.7.E+05	2.8.E+01
Apaf1	141 kDa	11	0.99997321	2.3.E+05	6.4.E+06	2.8.E+01
Loxl1	67 kDa	1	0.9790477	4.6.E+03	1.3.E+05	2.8.E+01
Mcl1	35 kDa	4	0.99999875	1.1.E+05	3.2.E+06	2.8.E+01
Dhx8	143 kDa	29	0.99998945	1.4.E+06	4.1.E+07	2.8.E+01
Larp7	65 kDa	14	0.99999973	9.7.E+05	2.7.E+07	2.8.E+01
L1td1	88 kDa	35	0.99999999	4.1.E+07	1.2.E+09	2.8.E+01
Nid1	137 kDa	18	0.99999939	1.8.E+06	5.1.E+07	2.8.E+01
Zswim8	197 kDa	2	0.9989186	1.3.E+04	3.7.E+05	2.8.E+01
U2surp	118 kDa	20	0.99999667	2.1.E+06	5.8.E+07	2.8.E+01
Zcchc7	63 kDa	2	0.99540453	4.7.E+04	1.3.E+06	2.8.E+01
Exo1	92 kDa	1	0.99459092	1.4.E+04	3.9.E+05	2.8.E+01
Srsf3	19 kDa	4	0.99946109	7.5.E+06	2.1.E+08	2.8.E+01
Bbs2	80 kDa	3	0.99952609	1.1.E+04	3.2.E+05	2.8.E+01
Tpm4	28 kDa	6	0.99871428	3.4.E+05	9.6.E+06	2.8.E+01
Ybx1	36 kDa	4	0.99990542	4.0.E+04	1.1.E+06	2.8.E+01
Tnip1	73 kDa	6	0.99161208	6.3.E+04	1.8.E+06	2.8.E+01
Zfp36l1	36 kDa	2	0.99689246	1.6.E+05	4.6.E+06	2.8.E+01
Klhl26	67 kDa	4	0.99851539	2.1.E+04	5.8.E+05	2.8.E+01
Dync2h1	492 kDa	5	0.9834279	7.4.E+04	2.1.E+06	2.8.E+01
Mrm3	47 kDa	4	0.9891597	6.0.E+04	1.7.E+06	2.8.E+01
Slc30a6	51 kDa	2	0.9896457	3.7.E+04	1.0.E+06	2.8.E+01
Prkcd	78 kDa	2	0.99953416	2.4.E+04	6.7.E+05	2.8.E+01
Maea	45 kDa	8	0.99931965	2.0.E+05	5.6.E+06	2.8.E+01
Degs1	38 kDa	3	0.99999881	2.8.E+05	7.8.E+06	2.8.E+01
Kntc1	250 kDa	31	0.99999201	1.1.E+06	3.1.E+07	2.8.E+01
Tjp1	195 kDa	50	1	8.3.E+06	2.3.E+08	2.8.E+01
Ppfibp2	99 kDa	4	0.99941793	1.3.E+04	3.7.E+05	2.8.E+01
Cdh1	98 kDa	12	0.99990268	2.3.E+06	6.3.E+07	2.8.E+01
Pprc1	175 kDa	5	0.99956866	3.6.E+04	9.8.E+05	2.8.E+01
Mbtd1	71 kDa	4	0.99812081	2.3.E+04	6.4.E+05	2.8.E+01
Dmwd	70 kDa	2	0.9846151	1.0.E+04	2.8.E+05	2.8.E+01
Usp4	108 kDa	18	0.99998873	5.7.E+05	1.6.E+07	2.8.E+01
Itprid2	137 kDa	10	0.9995573	3.7.E+05	1.0.E+07	2.8.E+01
Srxn1	14 kDa	2	0.99385301	2.6.E+04	7.2.E+05	2.7.E+01
Taf6	73 kDa	13	0.9998672	2.8.E+05	7.6.E+06	2.7.E+01
Dhx33	78 kDa	15	0.9997113	1.5.E+06	4.1.E+07	2.7.E+01
Ptpmt1	22 kDa	3	0.99968398	8.0.E+04	2.2.E+06	2.7.E+01
Dnmbp	177 kDa	7	0.99572237	7.9.E+04	2.2.E+06	2.7.E+01

Cep350	346 kDa	47	1	1.5.E+06	4.2.E+07	2.7.E+01
Polr3g	26 kDa	2	0.9882991	3.9.E+03	1.1.E+05	2.7.E+01
Fdxr	54 kDa	6	0.99822228	1.3.E+05	3.5.E+06	2.7.E+01
Lypla1	25 kDa	2	0.99011453	3.8.E+04	1.0.E+06	2.7.E+01
Fgd6	155 kDa	5	0.9955019	3.8.E+04	1.0.E+06	2.7.E+01
Msi2	37 kDa	2	0.99321438	1.5.E+05	4.2.E+06	2.7.E+01
Exoc6	93 kDa	9	0.99868111	1.9.E+05	5.1.E+06	2.7.E+01
Fn1	273 kDa	61	0.99999999	6.7.E+07	1.8.E+09	2.7.E+01
Naa16	101 kDa	9	0.9999787	3.3.E+04	9.1.E+05	2.7.E+01
Rabgef1	57 kDa	6	0.9992818	4.6.E+04	1.3.E+06	2.7.E+01
Cep57	57 kDa	12	0.99999906	9.8.E+05	2.6.E+07	2.7.E+01
Rap2c	21 kDa	2	0.99993713	8.4.E+04	2.3.E+06	2.7.E+01
Lyplal1	26 kDa	2	0.99896511	1.1.E+04	3.0.E+05	2.7.E+01
Als2	183 kDa	8	0.99954407	1.6.E+05	4.2.E+06	2.7.E+01
As3mt	42 kDa	3	0.99384504	6.5.E+04	1.8.E+06	2.7.E+01
Lama5	404 kDa	49	0.99999984	3.1.E+06	8.5.E+07	2.7.E+01
Ccdc115	20 kDa	1	0.9889052	6.4.E+03	1.7.E+05	2.7.E+01
Eif2ak4	186 kDa	8	0.99938335	5.7.E+04	1.5.E+06	2.7.E+01
Haus8	42 kDa	5	0.99987144	2.0.E+05	5.4.E+06	2.7.E+01
Peg10	110 kDa	8	0.99945554	2.5.E+05	6.8.E+06	2.7.E+01
Rnf10	88 kDa	7	0.9993198	1.2.E+05	3.3.E+06	2.7.E+01
Ak4	25 kDa	6	0.99998635	7.5.E+05	2.0.E+07	2.7.E+01
Actc1	42 kDa	19	0.99999091	1.0.E+08	2.8.E+09	2.7.E+01
Actc1	42 kDa	19	0.99999091	1.0.E+08	2.8.E+09	2.7.E+01
Acta1	42 kDa	19	0.99999091	1.0.E+08	2.8.E+09	2.7.E+01
Macroh2a2	40 kDa	11	0.99966502	3.2.E+05	8.6.E+06	2.7.E+01
Zgrf1	206 kDa	1	0.99620154	3.3.E+03	8.9.E+04	2.7.E+01
Mtx2	30 kDa	6	0.99999225	3.7.E+05	1.0.E+07	2.7.E+01
Pi4k2a	54 kDa	13	0.99992305	3.8.E+05	1.0.E+07	2.7.E+01
Spata2	58 kDa	3	0.9862429	4.9.E+04	1.3.E+06	2.7.E+01
Ofd1	117 kDa	22	0.99996739	9.3.E+05	2.5.E+07	2.7.E+01
Obi1	80 kDa	7	0.99792595	1.0.E+05	2.7.E+06	2.7.E+01
-	22 kDa	2	0.99363717	6.6.E+04	1.8.E+06	2.7.E+01
Lpcat3	56 kDa	2	0.99572223	1.7.E+05	4.4.E+06	2.7.E+01
Kif15	160 kDa	31	0.99999599	7.7.E+05	2.0.E+07	2.7.E+01
Tnk1	73 kDa	1	0.99991567	4.7.E+03	1.3.E+05	2.7.E+01
Proser1	92 kDa	4	0.99440643	7.1.E+04	1.9.E+06	2.7.E+01
Kif23	109 kDa	37	0.99999986	2.8.E+07	7.3.E+08	2.7.E+01
Cdyl2	56 kDa	2	0.99465688	1.6.E+04	4.2.E+05	2.7.E+01
Hook1	84 kDa	9	0.99990081	2.1.E+05	5.5.E+06	2.7.E+01

Tfam	28 kDa	4	0.99218251	1.1.E+05	2.8.E+06	2.7.E+01
Cetn3	20 kDa	6	0.99956248	7.6.E+05	2.0.E+07	2.7.E+01
-	26 kDa	3	0.99816321	9.1.E+04	2.4.E+06	2.7.E+01
Akap8l	71 kDa	6	0.9996875	1.5.E+05	3.9.E+06	2.7.E+01
Stk3	57 kDa	6	0.99657551	7.7.E+04	2.0.E+06	2.7.E+01
Pycr1	32 kDa	9	0.99977715	1.0.E+05	2.7.E+06	2.7.E+01
Rnf17	186 kDa	12	0.99948673	4.5.E+05	1.2.E+07	2.6.E+01
Actr1b	42 kDa	7	0.99956363	5.2.E+03	1.4.E+05	2.6.E+01
Cdk5rap1	66 kDa	6	0.99963121	1.1.E+05	2.9.E+06	2.6.E+01
Cyc1	35 kDa	3	0.99997937	2.8.E+05	7.4.E+06	2.6.E+01
Gprasp1	152 kDa	16	0.99999924	3.6.E+05	9.5.E+06	2.6.E+01
Arih1	64 kDa	2	0.99924792	2.8.E+04	7.4.E+05	2.6.E+01
Morf4l2	32 kDa	5	0.99064234	3.4.E+04	8.9.E+05	2.6.E+01
Lsm14b	42 kDa	1	0.99894431	5.8.E+04	1.5.E+06	2.6.E+01
Znf592	138 kDa	2	0.9893427	1.0.E+04	2.7.E+05	2.6.E+01
Spag5	130 kDa	13	0.99950399	2.7.E+05	7.0.E+06	2.6.E+01
Ppp6r3	95 kDa	14	0.99996717	4.1.E+05	1.1.E+07	2.6.E+01
Gtpbp6	56 kDa	4	0.9995376	6.0.E+04	1.6.E+06	2.6.E+01
Usp6nl	94 kDa	2	0.99374752	4.9.E+04	1.3.E+06	2.6.E+01
Dcaf1	169 kDa	7	0.99475288	8.4.E+04	2.2.E+06	2.6.E+01
Tex11	110 kDa	9	0.99107981	9.4.E+04	2.4.E+06	2.6.E+01
Cenpo	34 kDa	5	0.99805457	6.3.E+04	1.6.E+06	2.6.E+01
Aup1	46 kDa	4	0.99770174	9.4.E+04	2.5.E+06	2.6.E+01
Nudcd3	41 kDa	4	0.99822418	5.9.E+04	1.5.E+06	2.6.E+01
Zbtb24	79 kDa	17	0.99999989	1.4.E+06	3.6.E+07	2.6.E+01
Top2b	182 kDa	49	0.99999538	3.5.E+06	9.0.E+07	2.6.E+01
Ttc27	96 kDa	11	0.99988658	4.6.E+05	1.2.E+07	2.6.E+01
Lamc1	177 kDa	28	0.9999976	2.9.E+06	7.4.E+07	2.6.E+01
Cep152	197 kDa	51	0.99999894	4.0.E+06	1.0.E+08	2.6.E+01
Fanca	161 kDa	17	0.99918076	2.9.E+05	7.5.E+06	2.6.E+01
Ibtk	150 kDa	3	0.9939003	2.3.E+04	5.8.E+05	2.6.E+01
Fastkd5	87 kDa	3	0.99971267	5.6.E+04	1.4.E+06	2.6.E+01
Pomt2	92 kDa	3	0.99298824	3.8.E+04	9.8.E+05	2.6.E+01
Sars2	58 kDa	7	0.9999895	1.4.E+05	3.5.E+06	2.6.E+01
S100pbp	44 kDa	7	0.99644648	2.8.E+05	7.3.E+06	2.6.E+01
Fundc2	17 kDa	2	0.99953139	1.5.E+05	3.8.E+06	2.6.E+01
Med20	23 kDa	2	0.99458851	3.4.E+04	8.8.E+05	2.6.E+01
Ttf1	98 kDa	10	0.99999784	1.7.E+05	4.4.E+06	2.6.E+01
Htatip2	27 kDa	5	0.99937255	8.9.E+04	2.3.E+06	2.6.E+01
Timm50	40 kDa	7	0.99999054	2.0.E+06	5.2.E+07	2.6.E+01

Ska3	45 kDa	5	0.99943876	1.6.E+05	4.1.E+06	2.6.E+01
Dhx32	84 kDa	7	0.99980037	1.3.E+05	3.3.E+06	2.6.E+01
Mrpl50	18 kDa	3	0.99870115	1.2.E+05	3.2.E+06	2.6.E+01
Sf3a2	50 kDa	8	0.99999957	2.0.E+06	5.1.E+07	2.6.E+01
Zw10	88 kDa	11	0.99925726	2.7.E+05	6.8.E+06	2.6.E+01
Trpm7	212 kDa	12	0.99976098	1.1.E+05	2.8.E+06	2.6.E+01
Cpt1a	88 kDa	10	0.99999917	5.2.E+05	1.3.E+07	2.5.E+01
Ccdc88c	227 kDa	63	0.99997964	2.5.E+06	6.3.E+07	2.5.E+01
Snap29	30 kDa	3	0.99278883	5.9.E+04	1.5.E+06	2.5.E+01
Appl2	74 kDa	7	0.9972285	9.7.E+04	2.5.E+06	2.5.E+01
Spout1	43 kDa	6	0.99988375	1.9.E+05	4.7.E+06	2.5.E+01
Klc2	67 kDa	10	0.99710785	1.8.E+05	4.6.E+06	2.5.E+01
Snx3	19 kDa	4	0.99942597	4.0.E+05	1.0.E+07	2.5.E+01
Vps37b	31 kDa	3	0.99915923	1.0.E+04	2.5.E+05	2.5.E+01
Mvk	42 kDa	4	0.99893136	6.0.E+04	1.5.E+06	2.5.E+01
Rps6ka3	84 kDa	12	0.9995625	8.1.E+04	2.0.E+06	2.5.E+01
Emc2	35 kDa	2	0.99920571	2.7.E+04	6.8.E+05	2.5.E+01
Kpna4	58 kDa	4	0.9999604	5.8.E+04	1.5.E+06	2.5.E+01
Slc4a7	117 kDa	8	0.99952849	2.0.E+05	5.1.E+06	2.5.E+01
Kin	45 kDa	4	0.99816058	1.4.E+05	3.4.E+06	2.5.E+01
Rad51c	41 kDa	4	0.9997555	8.0.E+04	2.0.E+06	2.5.E+01
Smim20	8 kDa	1	0.9814211	3.9.E+04	9.7.E+05	2.5.E+01
Rps28	8 kDa	3	0.99992668	2.6.E+06	6.5.E+07	2.5.E+01
Baz2a	210 kDa	33	0.99999755	4.8.E+06	1.2.E+08	2.5.E+01
Ergic2	42 kDa	1	0.99129735	1.2.E+04	2.9.E+05	2.5.E+01
Ncor2	270 kDa	12	0.99895425	1.3.E+05	3.2.E+06	2.5.E+01
Otud3	45 kDa	1	0.99742715	4.4.E+03	1.1.E+05	2.5.E+01
Znf426	63 kDa	4	0.99939723	1.5.E+04	3.8.E+05	2.5.E+01
Phldb1	150 kDa	7	0.99586012	3.7.E+04	9.2.E+05	2.5.E+01
Rph3a	75 kDa	1	0.99987269	8.3.E+04	2.1.E+06	2.5.E+01
Zzz3	102 kDa	3	0.99941084	8.5.E+04	2.1.E+06	2.5.E+01
Znf653	68 kDa	2	0.9882105	7.9.E+03	2.0.E+05	2.5.E+01
Tpm2	33 kDa	8	0.99959752	2.6.E+05	6.5.E+06	2.5.E+01
Cdh3	91 kDa	8	0.99983915	2.3.E+05	5.8.E+06	2.5.E+01
Bcat1	43 kDa	3	0.99904646	1.5.E+05	3.7.E+06	2.5.E+01
Aagab	35 kDa	1	0.9834053	1.1.E+04	2.7.E+05	2.5.E+01
Mlh1	85 kDa	14	0.99987129	4.7.E+05	1.2.E+07	2.5.E+01
Rcbtb2	60 kDa	3	0.9858018	3.2.E+04	8.0.E+05	2.5.E+01
Chpt1	45 kDa	1	0.99567966	5.1.E+04	1.3.E+06	2.5.E+01
Meioc	109 kDa	5	0.99343139	2.9.E+04	7.1.E+05	2.5.E+01

Btrc	69 kDa	12	0.9996238	2.0.E+05	5.0.E+06	2.5.E+01
Mios	98 kDa	8	0.99934662	2.1.E+05	5.2.E+06	2.5.E+01
Ctdp1	105 kDa	6	0.99880258	1.3.E+05	3.2.E+06	2.5.E+01
Racgap1	70 kDa	21	0.99999959	1.2.E+07	3.0.E+08	2.5.E+01
Gorasp1	47 kDa	1	0.99825059	1.2.E+04	2.9.E+05	2.5.E+01
Exosc8	30 kDa	5	0.99961357	2.1.E+05	5.1.E+06	2.5.E+01
Ajuba	58 kDa	10	0.99999887	9.4.E+05	2.3.E+07	2.5.E+01
Tbx3	79 kDa	10	0.99998201	4.2.E+05	1.0.E+07	2.5.E+01
Commd6	10 kDa	2	0.99024924	5.5.E+04	1.4.E+06	2.5.E+01
Klhl7	66 kDa	8	0.99849233	1.2.E+05	2.9.E+06	2.5.E+01
Crocc	227 kDa	67	0.99999943	3.4.E+06	8.4.E+07	2.5.E+01
Nexn	72 kDa	4	0.99560661	5.8.E+04	1.4.E+06	2.5.E+01
Pcm1	229 kDa	36	0.99999799	2.8.E+06	6.9.E+07	2.5.E+01
Parl	42 kDa	4	0.9881177	2.2.E+05	5.4.E+06	2.5.E+01
Atat1	47 kDa	3	0.99918271	7.6.E+03	1.9.E+05	2.5.E+01
Aimp2	35 kDa	5	0.99983989	9.4.E+04	2.3.E+06	2.5.E+01
Spin1	30 kDa	5	0.99723546	9.4.E+04	2.3.E+06	2.5.E+01
Xpo5	137 kDa	22	0.99996056	1.7.E+06	4.0.E+07	2.4.E+01
Prpf38a	37 kDa	6	0.99999921	3.2.E+06	7.9.E+07	2.4.E+01
Ccdc77	58 kDa	15	0.99998484	1.6.E+06	4.0.E+07	2.4.E+01
Cep68	79 kDa	12	0.99999894	6.5.E+05	1.6.E+07	2.4.E+01
Lyst	425 kDa	4	0.9889973	1.0.E+04	2.5.E+05	2.4.E+01
Rps6ka5	97 kDa	4	0.99097558	1.7.E+04	4.2.E+05	2.4.E+01
Arpc5l	17 kDa	3	0.99978657	9.0.E+05	2.2.E+07	2.4.E+01
Ube2c	20 kDa	6	0.99996297	7.2.E+05	1.8.E+07	2.4.E+01
Fbxo30	83 kDa	8	0.99743009	1.5.E+05	3.7.E+06	2.4.E+01
Ilf3	96 kDa	30	0.99999998	8.8.E+06	2.1.E+08	2.4.E+01
Arhgap39	125 kDa	5	0.99246346	5.2.E+04	1.3.E+06	2.4.E+01
Plod3	85 kDa	5	0.99893784	1.1.E+05	2.7.E+06	2.4.E+01
Pars2	54 kDa	5	0.99951354	7.0.E+04	1.7.E+06	2.4.E+01
Aldh3a2	54 kDa	4	0.99527753	5.2.E+04	1.3.E+06	2.4.E+01
Cntrob	99 kDa	29	0.99999983	5.1.E+06	1.2.E+08	2.4.E+01
Erc1	128 kDa	13	0.99947949	1.7.E+05	4.0.E+06	2.4.E+01
Tmem186	25 kDa	1	0.9920414	1.4.E+04	3.3.E+05	2.4.E+01
Bclaf3	90 kDa	31	0.99999983	8.1.E+06	2.0.E+08	2.4.E+01
Dusp12	37 kDa	3	0.99971083	5.3.E+04	1.3.E+06	2.4.E+01
Syk	71 kDa	4	0.99883541	3.6.E+04	8.7.E+05	2.4.E+01
Pex6	105 kDa	15	0.99936314	3.7.E+05	8.9.E+06	2.4.E+01
Vwa8	213 kDa	7	0.99876878	1.2.E+05	2.9.E+06	2.4.E+01
Atg3	36 kDa	4	0.998557	8.4.E+04	2.0.E+06	2.4.E+01

Trappc6b	18 kDa	1	0.9856546	2.1.E+04	5.0.E+05	2.4.E+01
Ric1	159 kDa	5	0.99758592	5.9.E+04	1.4.E+06	2.4.E+01
Plekha6	131 kDa	11	0.99991484	2.9.E+05	6.9.E+06	2.4.E+01
Stn1	43 kDa	4	0.99980274	7.0.E+04	1.7.E+06	2.4.E+01
Tubgcp6	197 kDa	2	0.9784525	3.6.E+04	8.6.E+05	2.4.E+01
Ptprs	212 kDa	5	0.9907256	1.7.E+04	4.0.E+05	2.4.E+01
Cc2d1a	104 kDa	6	0.99777822	8.5.E+04	2.0.E+06	2.4.E+01
Pcnt	329 kDa	108	1	1.8.E+07	4.3.E+08	2.4.E+01
Ripk3	53 kDa	9	0.99999524	4.2.E+05	9.9.E+06	2.4.E+01
Myef2	63 kDa	18	0.99999695	4.5.E+06	1.1.E+08	2.4.E+01
Ccdc61	57 kDa	8	0.99999958	9.4.E+05	2.2.E+07	2.4.E+01
Ralgapa2	210 kDa	7	0.99370237	5.0.E+04	1.2.E+06	2.4.E+01
Cdk5rap2	206 kDa	53	0.99999999	4.7.E+06	1.1.E+08	2.4.E+01
Cyth2	47 kDa	5	0.99870533	1.3.E+05	3.2.E+06	2.4.E+01
Get1	20 kDa	1	0.99071867	1.5.E+04	3.6.E+05	2.4.E+01
Prodh	68 kDa	7	0.99990181	2.0.E+05	4.8.E+06	2.4.E+01
Eny2	12 kDa	1	0.99999584	5.4.E+04	1.3.E+06	2.4.E+01
Mapk8ip3	148 kDa	3	0.9794358	7.0.E+03	1.7.E+05	2.4.E+01
Nid2	154 kDa	29	0.99999995	8.9.E+06	2.1.E+08	2.4.E+01
Cept1	46 kDa	2	0.9991215	1.3.E+06	3.0.E+07	2.4.E+01
Isg20l2	41 kDa	12	0.99997993	2.7.E+06	6.3.E+07	2.4.E+01
C6.1al	33 kDa	1	0.99521823	1.3.E+05	3.0.E+06	2.4.E+01
Rbm34	49 kDa	15	0.99998473	3.5.E+06	8.2.E+07	2.4.E+01
Aimp1	34 kDa	8	0.99996364	7.4.E+05	1.8.E+07	2.4.E+01
Rab4a	24 kDa	5	0.99992898	7.9.E+04	1.9.E+06	2.4.E+01
Rere	172 kDa	3	0.99285483	6.3.E+04	1.5.E+06	2.3.E+01
Ticrr	208 kDa	7	0.99385499	1.3.E+05	3.0.E+06	2.3.E+01
Tfpt	29 kDa	1	0.99825725	2.0.E+04	4.7.E+05	2.3.E+01
Ttc39b	70 kDa	4	0.99962038	7.9.E+04	1.9.E+06	2.3.E+01
Usp36	120 kDa	17	0.99999999	7.8.E+05	1.8.E+07	2.3.E+01
Ngdn	36 kDa	8	0.99996976	2.5.E+06	5.9.E+07	2.3.E+01
Nr3c1	86 kDa	5	0.99991273	7.5.E+04	1.8.E+06	2.3.E+01
Tradd	35 kDa	4	0.99556935	1.2.E+05	2.7.E+06	2.3.E+01
Ubxn1	34 kDa	5	0.9998507	9.3.E+05	2.2.E+07	2.3.E+01
Rlim	66 kDa	7	0.99991347	1.6.E+05	3.6.E+06	2.3.E+01
Lmnb2	67 kDa	26	0.99992905	3.6.E+06	8.3.E+07	2.3.E+01
Sirt1	80 kDa	9	0.99998658	1.9.E+05	4.4.E+06	2.3.E+01
Tmco6	55 kDa	4	0.99901493	1.1.E+05	2.5.E+06	2.3.E+01
Sorbs1	143 kDa	6	0.99909365	1.1.E+05	2.6.E+06	2.3.E+01
Htra1	51 kDa	6	0.99972515	2.3.E+05	5.2.E+06	2.3.E+01

Psma1	30 kDa	3	0.99925982	1.2.E+05	2.9.E+06	2.3.E+01
Arhgap32	230 kDa	25	0.99998451	8.7.E+05	2.0.E+07	2.3.E+01
Cep57l1	47 kDa	7	0.99979828	2.9.E+05	6.8.E+06	2.3.E+01
Fxr2	74 kDa	11	0.9999998	1.1.E+06	2.5.E+07	2.3.E+01
Ccdc18	170 kDa	46	0.99999858	3.0.E+06	6.9.E+07	2.3.E+01
Ddx11	102 kDa	10	0.99922507	1.6.E+05	3.6.E+06	2.3.E+01
Exoc8	81 kDa	6	0.99894862	1.3.E+05	2.9.E+06	2.3.E+01
Naa38	13 kDa	3	0.99195694	5.4.E+04	1.3.E+06	2.3.E+01
Myo1d	116 kDa	39	0.99999986	6.5.E+06	1.5.E+08	2.3.E+01
Lims1	37 kDa	7	0.99953541	2.0.E+05	4.6.E+06	2.3.E+01
Nrm	29 kDa	3	0.99996318	4.6.E+05	1.1.E+07	2.3.E+01
Smarca1	122 kDa	14	0.99044572	6.7.E+04	1.6.E+06	2.3.E+01
Agpat5	42 kDa	3	0.99979977	8.1.E+04	1.9.E+06	2.3.E+01
Acap2	87 kDa	6	0.99960295	1.2.E+05	2.8.E+06	2.3.E+01
Fkbp8	44 kDa	5	0.99998023	1.7.E+05	3.9.E+06	2.3.E+01
Arl6ip1	23 kDa	2	0.99900656	1.1.E+05	2.6.E+06	2.3.E+01
Cep120	113 kDa	25	0.99995568	1.5.E+06	3.4.E+07	2.3.E+01
-	20 kDa	2	0.99970051	3.8.E+04	8.8.E+05	2.3.E+01
Shcbp1	76 kDa	12	0.99964391	4.5.E+05	1.0.E+07	2.3.E+01
Prdm15	133 kDa	11	0.99897023	2.4.E+05	5.5.E+06	2.3.E+01
Stag3	141 kDa	8	0.99875544	1.9.E+05	4.4.E+06	2.3.E+01
Sap30	23 kDa	1	0.9860412	1.1.E+04	2.4.E+05	2.3.E+01
Alg1	54 kDa	4	0.99526912	8.2.E+04	1.9.E+06	2.3.E+01
Ddx47	51 kDa	16	0.99999945	4.7.E+06	1.1.E+08	2.3.E+01
Slx4	172 kDa	7	0.99398456	7.1.E+04	1.6.E+06	2.3.E+01
Nol8	129 kDa	20	0.99999974	1.4.E+06	3.1.E+07	2.3.E+01
Pias1	72 kDa	4	0.99980568	5.2.E+04	1.2.E+06	2.3.E+01
Lima1	84 kDa	24	0.99999999	7.6.E+06	1.7.E+08	2.3.E+01
Shroom3	215 kDa	9	0.99963709	1.0.E+05	2.4.E+06	2.3.E+01
Magt1	38 kDa	3	0.99435012	1.1.E+05	2.4.E+06	2.3.E+01
Khnyn	75 kDa	4	0.9997321	5.5.E+04	1.3.E+06	2.3.E+01
Lamb2	197 kDa	38	0.99999934	2.6.E+06	6.0.E+07	2.3.E+01
Lamtor1	18 kDa	4	0.99991326	3.4.E+05	7.7.E+06	2.3.E+01
Ccp110	111 kDa	15	0.99975775	3.3.E+05	7.6.E+06	2.3.E+01
Hax1	32 kDa	2	0.99375824	8.3.E+04	1.9.E+06	2.3.E+01
Ahctf1	248 kDa	58	0.99999966	7.8.E+06	1.8.E+08	2.3.E+01
Cul4a	88 kDa	14	0.99962455	9.6.E+04	2.2.E+06	2.3.E+01
Klf9	27 kDa	4	0.985959	2.8.E+04	6.4.E+05	2.3.E+01
Kifap3	91 kDa	7	0.99959439	1.6.E+05	3.7.E+06	2.3.E+01
Etv4	54 kDa	1	0.9827749	4.1.E+04	9.4.E+05	2.3.E+01

Etv4	54 kDa	1	0.9827749	4.1.E+04	9.4.E+05	2.3.E+01
Etv1	55 kDa	1	0.9827749	4.1.E+04	9.4.E+05	2.3.E+01
Etv5	58 kDa	1	0.9827749	4.1.E+04	9.4.E+05	2.3.E+01
Myo9a	292 kDa	12	0.99956856	8.9.E+04	2.0.E+06	2.3.E+01
Sun1	102 kDa	27	0.99999821	5.5.E+06	1.2.E+08	2.3.E+01
Atm	349 kDa	25	0.99996213	6.1.E+05	1.4.E+07	2.3.E+01
Arhgap44	89 kDa	4	0.99898631	2.8.E+04	6.3.E+05	2.3.E+01
Isca1	14 kDa	2	0.9896277	5.0.E+04	1.1.E+06	2.3.E+01
Mrtfa	103 kDa	4	0.99991723	1.6.E+05	3.7.E+06	2.3.E+01
Smg1	410 kDa	15	0.9994118	1.3.E+05	3.0.E+06	2.3.E+01
Anxa4	36 kDa	2	0.99187188	7.7.E+03	1.7.E+05	2.3.E+01
Plec	534 kDa	179	0.99999958	5.1.E+07	1.2.E+09	2.2.E+01
Ppp1r13l	89 kDa	3	0.99772476	6.3.E+04	1.4.E+06	2.2.E+01
Ccdc160	37 kDa	6	0.99971245	5.0.E+05	1.1.E+07	2.2.E+01
Sec61a2	52 kDa	7	0.99999439	6.4.E+05	1.4.E+07	2.2.E+01
Szt2	378 kDa	6	0.99931117	8.7.E+05	1.9.E+07	2.2.E+01
H2-K1	41 kDa	4	0.9983706	6.7.E+04	1.5.E+06	2.2.E+01
Mindy1	51 kDa	3	0.99996494	9.4.E+04	2.1.E+06	2.2.E+01
Sugp1	73 kDa	17	0.99999659	1.3.E+06	3.0.E+07	2.2.E+01
Cep41	41 kDa	2	0.99880844	1.9.E+04	4.3.E+05	2.2.E+01
Ctnna1	100 kDa	31	0.99999936	9.9.E+06	2.2.E+08	2.2.E+01
Col4a3	162 kDa	1	0.99980308	1.5.E+05	3.3.E+06	2.2.E+01
Col4a1	161 kDa	4	0.99988576	3.5.E+05	7.7.E+06	2.2.E+01
Heatr3	74 kDa	6	0.99996384	3.8.E+05	8.5.E+06	2.2.E+01
Thoc6	37 kDa	9	0.99998919	1.2.E+06	2.8.E+07	2.2.E+01
Cenpq	31 kDa	2	0.99749959	5.6.E+04	1.3.E+06	2.2.E+01
Kank2	90 kDa	16	0.99998235	1.6.E+06	3.6.E+07	2.2.E+01
Tbc1d15	77 kDa	13	0.99995772	7.3.E+05	1.6.E+07	2.2.E+01
Dop1b	257 kDa	7	0.9961496	6.4.E+04	1.4.E+06	2.2.E+01
Rbm10	103 kDa	13	0.9999997	1.5.E+06	3.2.E+07	2.2.E+01
Vps28	25 kDa	4	0.99961041	1.5.E+05	3.3.E+06	2.2.E+01
Cep72	72 kDa	18	0.99996084	1.8.E+06	3.9.E+07	2.2.E+01
Skt	213 kDa	62	0.99999973	1.3.E+07	2.8.E+08	2.2.E+01
Itga5	115 kDa	2	0.99849605	3.0.E+04	6.7.E+05	2.2.E+01
Dcaf8	66 kDa	4	0.99920622	1.5.E+05	3.4.E+06	2.2.E+01
Cdc20	55 kDa	5	0.9999225	2.0.E+05	4.5.E+06	2.2.E+01
Hook2	83 kDa	10	0.99823414	2.9.E+05	6.4.E+06	2.2.E+01
Mrpl38	45 kDa	5	0.99955871	1.8.E+05	4.1.E+06	2.2.E+01
Ppp1r21	88 kDa	3	0.99944113	3.3.E+04	7.2.E+05	2.2.E+01
Nfs1	51 kDa	6	0.99989136	1.2.E+05	2.8.E+06	2.2.E+01

Top1	91 kDa	26	0.99999833	1.3.E+07	2.9.E+08	2.2.E+01
Ciapin1	33 kDa	4	0.99995144	1.8.E+05	4.0.E+06	2.2.E+01
Aco1	98 kDa	10	0.99997004	4.1.E+05	9.1.E+06	2.2.E+01
Ldah	37 kDa	1	0.99877615	5.4.E+04	1.2.E+06	2.2.E+01
Smtnl2	50 kDa	4	0.99757516	1.8.E+05	4.0.E+06	2.2.E+01
Chchd3	26 kDa	8	0.99999986	1.6.E+06	3.6.E+07	2.2.E+01
Cebpzos	9 kDa	2	0.99877943	5.6.E+04	1.2.E+06	2.2.E+01
Smndc1	27 kDa	2	0.99215806	1.3.E+05	2.9.E+06	2.2.E+01
Gsr	54 kDa	1	0.99797642	6.7.E+03	1.5.E+05	2.2.E+01
Foxo3	71 kDa	1	0.9920403	8.2.E+03	1.8.E+05	2.2.E+01
Use1	31 kDa	6	0.99981606	2.2.E+05	4.8.E+06	2.2.E+01
Tnk2	117 kDa	4	0.9867799	7.9.E+04	1.7.E+06	2.2.E+01
Timm23	22 kDa	3	0.99958757	2.3.E+05	5.1.E+06	2.2.E+01
Taf1	214 kDa	23	0.99997211	5.5.E+05	1.2.E+07	2.2.E+01
Sirt4	38 kDa	2	0.9954677	2.8.E+04	6.2.E+05	2.2.E+01
Rpap3	74 kDa	6	0.99981795	1.5.E+05	3.4.E+06	2.2.E+01
Stk25	48 kDa	4	0.99902804	2.3.E+04	5.1.E+05	2.2.E+01
Sipa1l1	197 kDa	35	0.99999801	1.8.E+06	3.9.E+07	2.2.E+01
Atad3	67 kDa	19	0.99999707	3.7.E+06	8.1.E+07	2.2.E+01
Pias3	68 kDa	4	0.99066124	3.2.E+04	7.0.E+05	2.2.E+01
Mpnd	53 kDa	2	0.99238148	7.4.E+04	1.6.E+06	2.2.E+01
Mgme1	38 kDa	3	0.99645382	7.7.E+04	1.7.E+06	2.2.E+01
Klhdc4	65 kDa	2	0.99346046	1.7.E+04	3.8.E+05	2.2.E+01
Abracl	9 kDa	1	0.99737969	5.1.E+04	1.1.E+06	2.2.E+01
-	47 kDa	4	0.99985167	1.3.E+05	2.9.E+06	2.2.E+01
Kri1	82 kDa	7	0.99999839	2.1.E+05	4.7.E+06	2.2.E+01
Casp8ap2	219 kDa	6	0.99937414	5.2.E+04	1.1.E+06	2.2.E+01
Kif13a	196 kDa	3	0.99995677	5.0.E+04	1.1.E+06	2.2.E+01
Rplp0	34 kDa	8	0.99997459	1.4.E+07	2.9.E+08	2.2.E+01
Zc3hav1	107 kDa	10	0.99989638	5.3.E+05	1.2.E+07	2.2.E+01
Mycbp	12 kDa	2	0.99707909	4.0.E+04	8.7.E+05	2.2.E+01
Cyp20a1	52 kDa	2	0.99957483	4.0.E+04	8.6.E+05	2.2.E+01
Ptpn21	133 kDa	7	0.99717923	6.4.E+04	1.4.E+06	2.2.E+01
Cdk5	33 kDa	1	0.99069339	6.7.E+03	1.4.E+05	2.2.E+01
Sntb2	56 kDa	15	0.99999961	1.4.E+06	3.0.E+07	2.2.E+01
Mllt10	113 kDa	8	0.99972274	1.1.E+05	2.5.E+06	2.2.E+01
Rnf14	55 kDa	2	0.9829764	6.2.E+04	1.3.E+06	2.2.E+01
Nudcd1	67 kDa	9	0.9999977	2.2.E+05	4.8.E+06	2.2.E+01
Ptbp3	57 kDa	6	0.99999904	9.4.E+05	2.0.E+07	2.2.E+01
Lias	42 kDa	2	0.99904153	1.6.E+05	3.4.E+06	2.2.E+01

Plekhm1	119 kDa	7	0.99912877	8.8.E+04	1.9.E+06	2.2.E+01
Pum2	114 kDa	12	0.99994867	3.7.E+05	8.0.E+06	2.2.E+01
Cnbp	20 kDa	3	0.99999631	4.0.E+04	8.7.E+05	2.2.E+01
Galt	43 kDa	1	0.9977572	2.1.E+04	4.4.E+05	2.2.E+01
Rb1cc1	182 kDa	7	0.99884198	7.6.E+04	1.6.E+06	2.2.E+01
Stmn2	21 kDa	5	0.99999449	1.6.E+05	3.5.E+06	2.2.E+01
Trmt1l	81 kDa	8	0.99864936	3.5.E+05	7.5.E+06	2.2.E+01
Eif2b1	34 kDa	6	0.99932142	2.2.E+05	4.8.E+06	2.2.E+01
Eif4enif1	108 kDa	13	0.99962715	4.2.E+05	9.1.E+06	2.2.E+01
Hikeshi	22 kDa	4	0.99969555	1.3.E+05	2.9.E+06	2.2.E+01
Pabpc1	71 kDa	22	0.99999919	2.1.E+07	4.6.E+08	2.1.E+01
Ubac1	46 kDa	3	0.9854828	6.0.E+04	1.3.E+06	2.1.E+01
Gtf2h3	34 kDa	3	0.9990856	1.6.E+05	3.5.E+06	2.1.E+01
Snrpg	8 kDa	2	0.99965899	5.5.E+05	1.2.E+07	2.1.E+01
Arhgap11a	108 kDa	7	0.99995694	1.3.E+05	2.8.E+06	2.1.E+01
Col18a1	182 kDa	7	0.9998184	1.8.E+06	3.8.E+07	2.1.E+01
Ankrd26	181 kDa	33	0.99996026	8.3.E+05	1.8.E+07	2.1.E+01
Mex3d	65 kDa	1	0.9796976	2.3.E+04	5.0.E+05	2.1.E+01
Setd6	53 kDa	4	0.99505542	3.4.E+04	7.4.E+05	2.1.E+01
Rhot1	72 kDa	8	0.99999503	9.1.E+05	2.0.E+07	2.1.E+01
Nqo1	31 kDa	3	0.99655368	1.3.E+05	2.8.E+06	2.1.E+01
Upf2	148 kDa	13	0.99942047	4.0.E+05	8.6.E+06	2.1.E+01
Uqcc3	10 kDa	2	0.99702765	8.2.E+04	1.8.E+06	2.1.E+01
Mrps27	48 kDa	7	0.99857168	2.2.E+05	4.7.E+06	2.1.E+01
Sun2	82 kDa	17	0.99997311	6.7.E+05	1.4.E+07	2.1.E+01
Trim8	62 kDa	4	0.99932369	9.6.E+04	2.0.E+06	2.1.E+01
Trim71	92 kDa	20	0.99998062	3.0.E+06	6.5.E+07	2.1.E+01
Krt8	55 kDa	21	0.99904145	6.3.E+05	1.3.E+07	2.1.E+01
Gga1	70 kDa	3	0.99979402	1.1.E+05	2.4.E+06	2.1.E+01
Wdr6	122 kDa	9	0.99471998	2.7.E+05	5.8.E+06	2.1.E+01
Nsmce3	31 kDa	6	0.99999195	2.2.E+05	4.6.E+06	2.1.E+01
Sipa1l2	189 kDa	27	0.99986004	8.6.E+05	1.8.E+07	2.1.E+01
Brpf1	137 kDa	7	0.99899795	9.0.E+04	1.9.E+06	2.1.E+01
Cog5	91 kDa	12	0.99958166	4.6.E+05	9.7.E+06	2.1.E+01
Iqcb1	69 kDa	8	0.99974133	2.8.E+05	5.9.E+06	2.1.E+01
Snx8	52 kDa	4	0.99950359	2.6.E+05	5.5.E+06	2.1.E+01
Thada	217 kDa	13	0.99923359	3.0.E+05	6.3.E+06	2.1.E+01
Eif2ak2	58 kDa	7	0.99960306	1.3.E+05	2.8.E+06	2.1.E+01
Naprt	58 kDa	3	0.99778466	4.0.E+04	8.4.E+05	2.1.E+01
Tpm3	33 kDa	7	0.99925961	8.5.E+06	1.8.E+08	2.1.E+01

Sbf2	210 kDa	5	0.99949061	2.9.E+04	6.2.E+05	2.1.E+01
Timm29	29 kDa	2	0.99856202	2.2.E+04	4.6.E+05	2.1.E+01
Gpn3	33 kDa	3	0.99998708	9.3.E+04	2.0.E+06	2.1.E+01
Cep131	120 kDa	30	0.99984377	1.2.E+06	2.6.E+07	2.1.E+01
Mcm8	92 kDa	11	0.99772555	2.0.E+05	4.2.E+06	2.1.E+01
Lsm6	9 kDa	1	0.9963612	4.4.E+04	9.3.E+05	2.1.E+01
Nemf	121 kDa	10	0.99736448	4.6.E+05	9.7.E+06	2.1.E+01
Cyld	107 kDa	4	0.99887064	6.5.E+04	1.4.E+06	2.1.E+01
Pkn1	104 kDa	5	0.99040052	7.0.E+04	1.5.E+06	2.1.E+01
Znf830	41 kDa	1	0.9865171	3.3.E+03	6.9.E+04	2.1.E+01
Hspg2	398 kDa	72	0.99999998	2.8.E+07	5.9.E+08	2.1.E+01
Gpatch1	103 kDa	11	0.99985354	5.6.E+05	1.2.E+07	2.1.E+01
Crot	70 kDa	4	0.99601323	3.3.E+04	6.9.E+05	2.1.E+01
Prkci	68 kDa	8	0.99999967	2.6.E+05	5.5.E+06	2.1.E+01
Znf281	97 kDa	12	0.99989156	1.2.E+06	2.5.E+07	2.1.E+01
Ndufa8	20 kDa	3	0.99980467	1.0.E+05	2.1.E+06	2.1.E+01
Vim	54 kDa	28	0.99999927	2.3.E+07	4.9.E+08	2.1.E+01
Dnajc9	30 kDa	7	0.9996118	6.0.E+05	1.3.E+07	2.1.E+01
Itga3	117 kDa	2	0.99018858	4.9.E+04	1.0.E+06	2.1.E+01
Ddx50	82 kDa	14	0.9999254	4.2.E+05	8.6.E+06	2.1.E+01
Stk11ip	118 kDa	8	0.9988319	1.4.E+05	2.9.E+06	2.1.E+01
Plxnb2	206 kDa	11	0.99993716	1.9.E+05	4.0.E+06	2.1.E+01
Prkdc	471 kDa	36	0.99993448	9.0.E+05	1.9.E+07	2.1.E+01
Dusp11	38 kDa	3	0.99647275	6.4.E+04	1.3.E+06	2.1.E+01
Atp9a	119 kDa	5	0.99516449	1.0.E+05	2.1.E+06	2.1.E+01
Tmed8	36 kDa	1	0.99491521	7.2.E+03	1.5.E+05	2.1.E+01
Wdr41	52 kDa	1	0.99094503	9.0.E+03	1.9.E+05	2.1.E+01
Cep128	129 kDa	42	0.99999856	1.6.E+06	3.4.E+07	2.1.E+01
Wdr62	167 kDa	9	0.99779736	1.2.E+05	2.4.E+06	2.1.E+01
Mettl2	44 kDa	4	0.99687103	7.8.E+04	1.6.E+06	2.1.E+01
Ostc	17 kDa	2	0.9999731	2.3.E+05	4.9.E+06	2.1.E+01
Eloc	12 kDa	4	0.99898264	2.5.E+05	5.2.E+06	2.1.E+01
Zc3h13	204 kDa	8	0.99749419	1.8.E+05	3.8.E+06	2.1.E+01
Washc1	52 kDa	5	0.99967455	1.7.E+05	3.6.E+06	2.1.E+01
Camsap1	176 kDa	11	0.99997748	1.8.E+05	3.7.E+06	2.1.E+01
Qsox2	78 kDa	3	0.99091623	3.8.E+04	7.9.E+05	2.1.E+01
Abcb8	78 kDa	1	0.9879468	1.4.E+04	3.0.E+05	2.1.E+01
Tmem126b	25 kDa	2	0.99383383	1.2.E+05	2.4.E+06	2.1.E+01
Srsf5	31 kDa	4	0.99999987	6.1.E+05	1.3.E+07	2.1.E+01
Kank4	110 kDa	2	0.99963915	1.5.E+04	3.1.E+05	2.1.E+01

Tjp2	131 kDa	30	1	5.9.E+06	1.2.E+08	2.1.E+01
Lmbrd2	81 kDa	2	0.9838926	1.5.E+04	3.0.E+05	2.1.E+01
Pus1	48 kDa	9	0.99991833	2.5.E+05	5.1.E+06	2.1.E+01
Cnot3	82 kDa	5	0.99990903	2.5.E+05	5.0.E+06	2.1.E+01
Sesn2	54 kDa	4	0.99995686	6.4.E+04	1.3.E+06	2.0.E+01
Amotl1	108 kDa	8	0.99990316	2.8.E+05	5.7.E+06	2.0.E+01
Eif3j1	29 kDa	3	0.9871177	2.9.E+04	5.9.E+05	2.0.E+01
Eif3j1	29 kDa	3	0.9871177	2.9.E+04	5.9.E+05	2.0.E+01
Eif3j2	29 kDa	3	0.9871177	2.9.E+04	5.9.E+05	2.0.E+01
Serac1	74 kDa	5	0.9808956	1.8.E+05	3.7.E+06	2.0.E+01
Tada2a	51 kDa	7	0.99984347	6.8.E+04	1.4.E+06	2.0.E+01
Cog1	109 kDa	7	0.99810515	1.1.E+05	2.1.E+06	2.0.E+01
Akap2	99 kDa	2	0.9973006	1.9.E+04	3.9.E+05	2.0.E+01
Utp14b	86 kDa	17	0.99999668	1.6.E+06	3.2.E+07	2.0.E+01
Sugp2	118 kDa	15	0.99998777	5.6.E+05	1.1.E+07	2.0.E+01
Tns3	156 kDa	16	0.99998357	7.9.E+05	1.6.E+07	2.0.E+01
Neurl4	168 kDa	15	0.99993575	3.3.E+05	6.8.E+06	2.0.E+01
Cdk4	34 kDa	9	0.99996576	5.6.E+05	1.1.E+07	2.0.E+01
Odf2	96 kDa	24	0.99998233	2.2.E+06	4.5.E+07	2.0.E+01
Pdzd4	87 kDa	3	0.9818831	3.2.E+04	6.4.E+05	2.0.E+01
Clptm1	75 kDa	5	0.99999734	2.8.E+05	5.7.E+06	2.0.E+01
Acaa2	42 kDa	6	0.99995773	4.5.E+05	9.1.E+06	2.0.E+01
Arid2	196 kDa	15	0.99972501	6.8.E+05	1.4.E+07	2.0.E+01
Wtap	44 kDa	6	0.99933151	1.6.E+05	3.3.E+06	2.0.E+01
Arl6ip6	25 kDa	2	0.99996595	1.0.E+05	2.1.E+06	2.0.E+01
Psmf1	30 kDa	2	0.99977236	1.4.E+05	2.9.E+06	2.0.E+01
Gatd1	23 kDa	3	0.99482315	7.0.E+04	1.4.E+06	2.0.E+01
Zcchc8	78 kDa	6	0.99998384	1.4.E+05	2.8.E+06	2.0.E+01
Mtf2	67 kDa	8	0.99958411	1.7.E+05	3.5.E+06	2.0.E+01
Tarbp2	39 kDa	5	0.99579057	1.2.E+05	2.5.E+06	2.0.E+01
Nvl	94 kDa	32	0.9999994	6.4.E+06	1.3.E+08	2.0.E+01
Fra10ac1	37 kDa	6	0.99450815	9.6.E+04	1.9.E+06	2.0.E+01
Scfd1	72 kDa	9	0.99988232	3.4.E+05	6.8.E+06	2.0.E+01
Syce1	38 kDa	4	0.99362036	4.3.E+04	8.6.E+05	2.0.E+01
Fbxw11	62 kDa	11	0.99585725	3.8.E+05	7.8.E+06	2.0.E+01
Ubap2l	117 kDa	11	0.99999955	6.4.E+05	1.3.E+07	2.0.E+01
Gemin5	167 kDa	41	0.99999906	3.9.E+06	7.9.E+07	2.0.E+01
Asf1b	22 kDa	2	0.99701738	1.0.E+05	2.1.E+06	2.0.E+01
Trim11	53 kDa	2	0.99565354	9.8.E+03	2.0.E+05	2.0.E+01
Wdhd1	124 kDa	13	0.99999969	3.9.E+05	7.9.E+06	2.0.E+01

Gid8	27 kDa	3	0.99923171	1.2.E+05	2.3.E+06	2.0.E+01
Diaph2	125 kDa	15	0.99994075	2.5.E+05	5.1.E+06	2.0.E+01
Tardbp	45 kDa	10	0.99999007	1.4.E+07	2.9.E+08	2.0.E+01
Tcp11	62 kDa	2	0.9835654	3.0.E+04	6.1.E+05	2.0.E+01
Elp2	93 kDa	8	0.99883173	2.9.E+05	5.9.E+06	2.0.E+01
Slc12a4	121 kDa	4	0.985432	9.0.E+04	1.8.E+06	2.0.E+01
Cwf19l2	103 kDa	11	0.99787345	2.1.E+05	4.3.E+06	2.0.E+01
Pdcd7	54 kDa	8	0.99999627	1.5.E+05	3.0.E+06	2.0.E+01
Brd9	67 kDa	5	0.999798	1.4.E+05	2.9.E+06	2.0.E+01
Tln1	270 kDa	53	0.99999705	4.8.E+06	9.7.E+07	2.0.E+01
Mnat1	36 kDa	7	0.99962276	2.2.E+05	4.4.E+06	2.0.E+01
Idh1	47 kDa	7	0.99986441	5.0.E+05	1.0.E+07	2.0.E+01
Ppp2r1b	66 kDa	16	0.99998052	7.5.E+05	1.5.E+07	2.0.E+01
Ntn1	68 kDa	4	0.99342203	2.7.E+04	5.4.E+05	2.0.E+01
Cpt2	74 kDa	9	0.99998992	3.2.E+05	6.4.E+06	2.0.E+01
Abl1	123 kDa	9	0.99776215	4.4.E+04	8.9.E+05	2.0.E+01
Dnajc10	91 kDa	8	0.99924492	3.3.E+05	6.7.E+06	2.0.E+01
H2bc7	14 kDa	4	0.99992325	2.6.E+07	5.3.E+08	2.0.E+01
H2bc7	14 kDa	4	0.99992325	2.6.E+07	5.3.E+08	2.0.E+01
H2bc14	14 kDa	4	0.99992325	2.6.E+07	5.3.E+08	2.0.E+01
H2bc3	14 kDa	4	0.99992325	2.6.E+07	5.3.E+08	2.0.E+01
H2bc9	14 kDa	4	0.99992325	2.6.E+07	5.3.E+08	2.0.E+01
Hist2h2bb	14 kDa	4	0.99992325	2.6.E+07	5.3.E+08	2.0.E+01
H2bc4	14 kDa	4	0.99992325	2.6.E+07	5.3.E+08	2.0.E+01
H2bc12	14 kDa	4	0.99992325	2.6.E+07	5.3.E+08	2.0.E+01
Hist1h2bp	14 kDa	4	0.99992325	2.6.E+07	5.3.E+08	2.0.E+01
Atxn7l3	39 kDa	1	0.9791356	1.3.E+04	2.6.E+05	2.0.E+01
Pir	32 kDa	1	0.9840003	2.4.E+04	4.8.E+05	2.0.E+01
