# Supplementary material for: Decreased expression of Ly-1 antibody reactive clone (Lyar) triggers enhanced adipogenesis of bone marrow mesenchymal stromal cells in aged bone marrow
Source: PLoS One. 2026 May 27;21(5):e0349780. doi: 10.1371/journal.pone.0349780 (PMC13215539; doi:10.1371/journal.pone.0349780)
Supplement: Attachment — Submitted filename: Responce letter.docx [file pone.0349780.s010.docx]

We appreciate the time and effort devoted to the review of our manuscript despite your busy schedule. As detailed below, we have carefully addressed each of your comments to the fullest extent possible. We would be grateful for the opportunity to have our revised manuscript evaluated again.

**Q1. Additional Editor Comments:**
Please include data demonstrating FGF2–PI3K–Akt1 signaling activity in Figure 4. Without showing this signaling cascade, it is premature to draw conclusions about its functional involvement.

A1. Your important comment regarding the direct involvement of AKT in regulating Lyar is greatly appreciated. A similar point was also raised by Reviewer 2. In accordance with these comments, we performed AKT1 knockdown and confirmed that Lyar expression decreases as a result. These new experimental data have been added to the revised manuscript as Figures 4E and 4F.

 **Reviewer 1 comments:**

Q1. Incomplete Mechanistic Elucidation of the Lyar–Brd2 Axis. While the interaction between Lyar and Brd2 is demonstrated, the precise molecular mechanism by which this complex suppresses adipogenesis is not defined.

A1. We appreciate this important comment. To address your question, we generated Lyar-overexpressing MSCs and examined the effects of Brd2 knockdown on differentiation. In this experiment, Lyar-overexpressing MSCs were cultured in adipogenic differentiation medium for 48 hours and then analyzed by RT-PCR.

As a result, Brd2 suppression increased the expression of early adipogenic markers such as PPARγ and C/EBPα. Although the inhibitory effect of Lyar on adipogenic differentiation is strong and Brd2 suppression alone was not sufficient to induce full adipogenic differentiation, these findings indicate that Lyar-mediated suppression of adipogenesis can be at least partially rescued by reducing Brd2 expression.

Q2. Ambiguous Functional Interplay Between Tak1 and Lyar. The model posits that Lyar determines the functional output of Tak1 signaling, but direct evidence is limited. The biochemical consequence of the Lyar–Tak1 interaction on Tak1's kinase activity or its downstream non-canonical pathways (e.g., p38/JNK activation) is not investigated, leaving the proposed "switch" mechanism speculative.

A2. We appreciate this important comment. As you pointed out, our study does not clarify how the TAK1–Lyar interaction influences TAK1 activity or other downstream pathways that may be involved. However, Lyar has been reported to participate in cell proliferation and the maintenance of stemness, and in our experiments we demonstrated that TAK1 and Lyar interact, and that suppression of Lyar reduces cellular self-renewal even under conditions of TAK1 overexpression.

That said, as you noted, proposing that Lyar functions as a switch regulating TAK1 activity would be speculative. Therefore, in the revised manuscript we have explicitly added this point as a limitation and clarified that the precise relationship between TAK1 and Lyar remains to be elucidated.

Q3. Lack of Rescue Experiments. The most functionally compelling evidence—demonstrating that forced expression of Lyar in aged BMMSCs can rescue the hyper-adipogenic phenotype—is absent. Such an experiment is critical to substantiate the claim that Lyar downregulation is a causative factor in age-enhanced adipogenesis.

A3. We have already demonstrated that forced expression of Lyar suppresses adipogenic differentiation. Unfortunately, we were unable to perform gene introduction into aged cells followed by drug selection in mouse MSCs. As is well recognized, mouse BM-MSCs are highly vulnerable to stress, and MSCs derived from aged mice in particular have limited proliferative capacity; therefore, our experiments could only be conducted using MSCs from young to adult mice.

As you appropriately noted, it would be important to examine whether senescent phenotypes can be reversed and to more directly demonstrate involvement in age-related marrow adiposity. Ideally, this would require the use of conditional transgenic models. However, generation of transgenic mice was beyond the scope of the present study. We have therefore added this point as a limitation in the revised manuscript.

Q4. Inadequate Discussion of the Broader Field and Study Limitations. The discussion does not sufficiently contextualize the findings within the existing literature on TGF-β signaling in stem cell aging. Furthermore, the limitations of the study, particularly the sole reliance on murine models and the preliminary nature of the mechanistic insights, should be explicitly acknowledged.

A4. We appreciate this comment. The present study focused on TGFβ–TAK1 signaling and Lyar, which is thought to act downstream and to play an important role in the switch between self-renewal and differentiation. As you pointed out, the original wording was indeed unclear; therefore, we have revised the manuscript to improve clarity and added the necessary explanations. In addition, we have carefully incorporated and clearly stated the limitations raised by two reviewers.

**Reviewer 2 comments:**

Q1. Overall, there is no description of the protocols, equipment, or detectors used. Furthermore, when protocols are not described, the references provided are insufficient to enable replication　(1-5)

A1. We appreciate your careful review. We have revised the manuscript in accordance with your comments.

Q2. The manuscript does not clearly describe the number of biological replicates used in each experiment, nor does it consistently report the statistical methods applied. To ensure transparency and reproducibility, I recommend adding a dedicated “Statistical Analysis” section at the end of the Methods. This section should specify the number of biological replicates for each assay, the statistical tests used, any adjustments for multiple comparisons, and the software employed for data analysis.

A2. In accordance with your suggestion, we have described the statistical analyses in an independent section. We have also specified the software used for the analyses in that section.

Q3. Lyar is a Tak1-interacting protein that modulates the proliferation and differentiation of BMMSCs 7. We previously demonstrated that Tak1 plays a pivotal role in regulating the proliferation and differentiation of BMMSCs.
Please cite the literature.

A3. We sincerely apologize for the oversight. The necessary text has now been appropriately cited.

Q4. we generated a stable embryonic stem (ES) cell line expressing HA-tagged Tak1 to ensure experimental reproducibility and minimize stress-related artifacts associated with transient transfection.
A description or citation is required regarding the creation of stable embryonic stem (ES) cell lines expressing Tak1 tagged with HA in M&M.

A4. We sincerely apologize for the omission of important information in the previous version of the manuscript. In the revised manuscript, we have included a description of the method used to generate HA-tagged TAK1-overexpressing ES cells.

Q5. Focusing on molecules with known roles in stem cell proliferation and differentiation, we identified Lyar as a candidate protein previously reported to be involved in cell proliferation (Figure 2A)
The authors state that they “focused on molecules with known roles in stem cell proliferation and differentiation, identifying Lyar as a candidate from among them.” However, the 146 proteins listed in the Supplementary file appear to include other nuclear factors similarly reported to be involved in stem cell properties or differentiation control. Providing more specific reasons for prioritizing Lyar for analysis—such as the strength of enrichment, prior reports in MSCs/ESCs, or specific literature context—would clarify the rationale for this candidate selection.
Lyar expression is downregulated in BMMSCs from aged mice and is regulated by Akt signaling hypothesized that the decreased expression of Lyar in aged BMMSCs may be attributed to impaired signaling through the FGF2–PI3K–Akt1 pathway
Although it has been suggested that FGF2–PI3K–Akt1 may positively regulate Lyar expression, the current data remain at the correlation level, and additional verification is needed to conclude a direct mechanistic link.
For example, confirming the response of Lyar expression to forced expression of Akt1 or siAkt1 would likely strengthen the argument.

A5. We greatly appreciate this important comment. As you pointed out, the list of proteins shown in the Supplemental file includes other molecules that could potentially be involved in self-renewal and differentiation. Our study was originally initiated to clarify how TAK1 regulates MSC self-renewal. From this perspective, we narrowed our focus to molecules that function in the nucleus as transcription factors or related accessory proteins and that could be directly linked to cellular self-renewal. Among these candidates, Lyar was selected because it is stably expressed at the protein level in MSCs and was reproducibly detected in TAK1 immunoprecipitation experiments.

As a detailed description of this selection process would be overly lengthy for the manuscript, we instead added a concise explanation stating that, among these molecules, we focused on Lyar because it has already been reported to be deeply involved in stem cell self-renewal.

Q10. The dual and context-dependent roles of Tak1 require clearer explanation
The Discussion highlights that Tak1 supports stem cell self-renewal but has also been reported to promote adipogenesis. In contrast, the current study shows that Tak1 overexpression suppresses adipogenic differentiation (Fig. 3A). The relationship between these findings and previously reported functions is not fully reconciled. A more explicit interpretation of how the current results fit into the broader context of Tak1 biology would improve clarity.

A10. We greatly appreciate this important comment. What we intended to emphasize is that whether TAK1 activation leads cells toward proliferation or adipogenic differentiation is highly dependent on the cellular context, and that the presence of Lyar plays a key role in determining this outcome. We believe that Figure 3 illustrates this point.

The present findings underscore the functional importance of TAK1 biology. Beyond its well-established roles in cell proliferation and inflammation, our results suggest that, through interactions with downstream factors, TAK1 may also influence aging-related changes and differentiation.

Q11. Interpretation of Lyar knockdown effects appears overstated
In Fig. 2E, both Runx2 and Pparγ are upregulated 48 hours after Lyar knockdown. The Discussion interprets this as evidence that Lyar suppression promotes lineage commitment. However, simultaneous upregulation of early osteogenic and adipogenic markers at such an early time point may instead reflect loss of stemness or a general differentiation-prone state, rather than commitment toward a specific lineage. Although the manuscript includes functional adipogenesis assays for Lyar overexpression (Fig. 2H), no functional readouts are provided for the knockdown condition. Without later-stage adipogenic markers or lipid accumulation data under knockdown, commitment cannot be conclusively inferred.

A11. We appreciate this important comment. In our experimental system, stable suppression of Lyar using shRNA or gene editing markedly inhibited BM-MSC proliferation, which prevented us from establishing Lyar-deficient cells. Consequently, it was not feasible to maintain Lyar knockdown cells in adipogenic differentiation culture for two weeks, and we were therefore unable to obtain data on late-stage adipogenesis or lipid accumulation as you suggested. We have added this point as a limitation in the revised manuscript.

Q12. The claim that Lyar regulates “the balance between self-renewal and adipogenic commitment” is not fully supported
Fig. 3A demonstrates that Lyar knockdown abolishes the anti-adipogenic effect of Tak1 overexpression. While this suggests involvement in adipogenic regulation, the study does not present data assessing self-renewal or stemness under these conditions.
Therefore, concluding that Lyar governs the “balance” between self-renewal and adipogenic commitment overreaches the available evidence.

A12. We appreciate this important comment. In response to your suggestion, we isolated BM-MSCs from adult mice, established cells with stable TAK1 overexpression, and performed Lyar knockdown. The results were consistent with our previous findings and confirmed that suppression of Lyar reduces BM-MSC self-renewal and proliferation. These data are presented as a Supplemental Figure.

Q13. Functional significance of the Lyar–Brd2 interaction is not demonstrated
The proteomics analysis and co-immunoprecipitation (Fig. 3C) convincingly show a physical interaction between Lyar and Brd2. However, no functional experiments (e.g., Brd2 knockdown, pharmacologic inhibition, or interaction-disruption assays) are provided to demonstrate that this interaction mediates the observed anti-adipogenic effect. Thus, statements implying that Lyar suppresses adipogenesis through Brd2 should be toned down or framed as a hypothesis rather than a demonstrated mechanism.

A13. We appreciate this important comment. A similar concern was also raised by Reviewer 1. To address your question, we generated Lyar-overexpressing MSCs and examined the impact of Brd2 knockdown on differentiation. In this experiment, Lyar-overexpressing MSCs were cultured in adipogenic differentiation medium for 48 hours and subsequently analyzed by RT-PCR.

As a result, suppression of Brd2 increased the expression of early adipogenic markers such as PPARγ and C/EBPα. Although the inhibitory effect of Lyar on adipogenic differentiation is strong and Brd2 suppression alone was not sufficient to induce full adipogenic differentiation, these findings indicate that Lyar-mediated suppression of adipogenesis can be at least partially rescued by reducing Brd2 expression.

Q14. Identification of upstream regulators of Lyar expression remains inconclusive
The proteomic analysis reveals increased senescence-associated markers and decreased Akt1/Hmgb2 levels in aged BMMSCs. However, these results describe the cellular state of aged BMMSCs, rather than identifying direct upstream regulators of Lyar.
The proposed involvement of the FGF2–PI3K–Akt1 pathway is intriguing, establishing causality requires additional experiments (e.g., Akt1 knockdown, constitutively active Akt rescue).

A14. We appreciate this important comment. As you pointed out, our study does not clarify how the TAK1–Lyar interaction affects TAK1 activity or other downstream pathways that may be involved. However, Lyar has already been reported to participate in cell proliferation and the maintenance of stemness, and in our experiments we demonstrated that TAK1 and Lyar interact, and that suppression of Lyar reduces cellular self-renewal even under conditions of TAK1 overexpression.

At the same time, as you noted, proposing that Lyar functions as a switch for TAK1 activity would be speculative. Therefore, in the revised manuscript we have explicitly acknowledged this as a limitation and clarified that the precise relationship between TAK1 and Lyar remains to be elucidated.

Q15. The mention of “aging-associated hypermethylation” lacks supporting evidence
The Discussion suggests that reduced Lyar expression in aged tissues may be caused by aging-associated promoter hypermethylation. However, no methylation analysis was performed in this study.
This statement should be rephrased as a potential hypothesis rather than an inferred mechanism.

A15. We sincerely apologize for the oversight. In accordance with your comment, we have removed the statements regarding age-related methylation.

Q16. Several conclusions rely on wording that is stronger than the supporting data
Terms such as “regulates,” “governs,” “represses,” and “critical modulator” appear in contexts where the data support only correlation or partial mechanistic involvement. Adjusting these to more cautious terms (e.g., “suggests,” “may contribute to,” or “is consistent with”) would improve scientific rigor.

A16. We appreciate this important comment. In accordance with your suggestion, we have revised the manuscript.

Q17. Missing methodological details in figure legends
Several figure legends do not provide essential experimental information. For example, Figures 2E and 2G do not specify the normalization gene used for qPCR, and the conditions for adipogenic induction in Figure 3A are not fully described. Including these details directly in each legend would improve clarity and reproducibility.

A17. We appreciate this important comment. In accordance with your suggestion, we have revised the manuscript.

Q18. Lack of quantitative data for image-based results
Some panels present only representative images without quantitative analysis, particularly the Oil Red O staining in Figures 2H and 3A and the Western blot in Figure 4B. Providing quantitative assessments—such as ORO absorbance, lipid droplet area, or densitometric analysis—would strengthen the conclusions derived from these figures. Additionally, the Oil Red O staining method should be added to the Materials and Methods section.

A18.　 We appreciate this comment. In our MSC experiments, suppression of Lyar resulted in growth arrest and increased cell death during differentiation, which made it difficult to obtain reliable quantitative data using Oil Red O staining. Therefore, we conducted the same experiments in 3T3-L1 cells and have added the quantitative Oil Red O data as supplemental data.

Q19. Absence of scale bars in microscopy images
Several microscopy images appear to lack scale bars, making it difficult to interpret morphological differences across conditions. Adding scale bars to all image panels would enhance interpretability.

A19. In accordance with your comment, we have added scale bars.

Q20. Insufficient annotation and clarity in Supplementary file 2
The proteomic dataset in Supplementary file 2 does not clearly indicate the thresholds used for differential protein expression, and key proteins highlighted in the main text (e.g., Akt1, Hmgb2, H2AX, Notch2) are not visually emphasized. Improving annotation—such as marking relevant proteins or providing summary plots—would help readers better understand how the proteomic results support the conclusions.

A20. We appreciate this important comment. We have highlighted Akt1, Hmgb2, H2AX, and Notch2, and have specified the threshold (defined as a change of ≥2-fold) in the figure legend.
