# Supplementary material for: Decreased expression of Ly-1 antibody reactive clone (Lyar) triggers enhanced adipogenesis of bone marrow mesenchymal stromal cells in aged bone marrow
Source: PLoS One. 2026 May 27;21(5):e0349780. doi: 10.1371/journal.pone.0349780 (PMC13215539; doi:10.1371/journal.pone.0349780)
Supplement: Attachment — Submitted filename: Responce Rev2.docx [file pone.0349780.s011.docx]

We would like to express our sincere gratitude for your careful and thorough re-evaluation of our manuscript.
We greatly appreciate your insightful and constructive comments, all of which we consider to be well-founded and highly valuable.
We have carefully addressed each of the points raised by Reviewer 2, as detailed below.

**Q1–6.** We have revised all statements that were overly definitive, potentially overestimated, or based on limited data. In particular, the relevant sections in the Discussion have been comprehensively revised. As numerous modifications were made, we would be grateful if you could refer to the revised text highlighted in red.

**Q7.** We have addressed this point by adding a new section describing the limitations of the study.

**Q8.** We have revised the figure legends accordingly. In addition, the adipogenic differentiation conditions have now been clearly described in the Materials and Methods section.

**Q9.** We have added the following statement to the relevant section of the Discussion:
Although the extent to which these mechanisms operate in primary BMMSCs remains to be determined, similar effects were observed in 3T3-L1 cells (Supplementary Figure 2). However, additional quantification in 3T3-L1 cells does not fully compensate for the limited quantitative support in primary BMMSCs, and therefore conclusions based on representative images should be interpreted with caution.

**Q10.** We have revised the Supplemental file to improve clarity and facilitate the reader’s understanding, including the addition of highlights.

**Q11.** We sincerely apologize for this omission and have now added the appropriate citation.
